# Estimating the causal effects of multiple intermittent treatments with application to COVID-19

**Published:** 2021-09-27

**Authors:** Liangyuan Hu, Fan Li, Jiayi Ji, Himanshu Joshi, Erick Scott

**Affiliations:** 1 Department of Biostatistics and Epidemiology, Rutgers University, Piscataway, NJ 08854, U.S.A.; 2 Department of Biostatistics, Yale School of Public Health, New Haven, Connecticut 06510, U.S.A.; 3 Department of Population Health Science and Policy, Icahn School of Medicine at Mount Sinai, New York, New York 10029, U.S.A.; 4 Karius Inc., Redwood City, CA 94065, U.S.A.

**Keywords:** Causal inference, Observational study, Stabilized inverse probability weights, Time-varying treatments, Recurrent events, Machine learning

## Abstract

To draw real-world evidence about the comparative effectiveness of multiple time-varying treatment regimens on patient survival, we develop a joint marginal structural proportional hazards model and novel weighting schemes in continuous time to account for time-varying confounding and censoring. Our methods formulate complex longitudinal treatments with multiple “start/stop” switches as the recurrent events with discontinuous intervals of treatment eligibility. We derive the weights in continuous time to handle a complex longitudinal dataset on its own terms, without the need to discretize or artificially align the measurement times. We further propose using machine learning models designed for censored survival data with time-varying covariates and the kernel function estimator of the baseline intensity to efficiently estimate the continuous-time weights. Our simulations demonstrate that the proposed methods provide better bias reduction and nominal coverage probability when analyzing observational longitudinal survival data with irregularly spaced time intervals, compared to conventional methods that require aligned measurement time points. We apply the proposed methods to a large-scale COVID-19 dataset to estimate the causal effects of several COVID-19 treatment strategies on in-hospital mortality or ICU admission, and provide new insights relative to findings from randomized trials.

## 1. Introduction

The COVID-19 pandemic has been a rapidly evolving crisis challenging global health and economies. Public health experts believe that this pandemic has no true precedent in modern times. While multiple COVID-19 vaccines have been developed across the globe, no consensus has been reached on optimal clinical management of COVID-19 (Yousefi et al., 2020). The lack of evidence for effective treatment options warrants further investigation into the causal effects of multiple COVID-19 treatment strategies currently implemented in clinics. Although randomized controlled trials (RCTs) are considered as the gold standard for evaluating the efficacy of COVID-19 therapies, they are enormously expensive and time consuming, especially in a time of crisis. Stringent inclusion and exclusion criteria also limit the generalizability of RCTs to frailer populations at higher risk for severe morbidity and mortality. To overcome these challenges, we study the causal effects of COVID-19 treatment strategies on patient survival by leveraging the continuously growing observational data collected at the Mount Sinai Health System—New York City’s largest academic medical system. We focus on four commonly used medication classes that are of most clinical interest: (i) remdesivir; (ii) dexamethasone; (iii) anti-inflammatory medications other than corticosteroids; and (iv) corticosteroids other than dexamethasone.

**Fig. 1.**
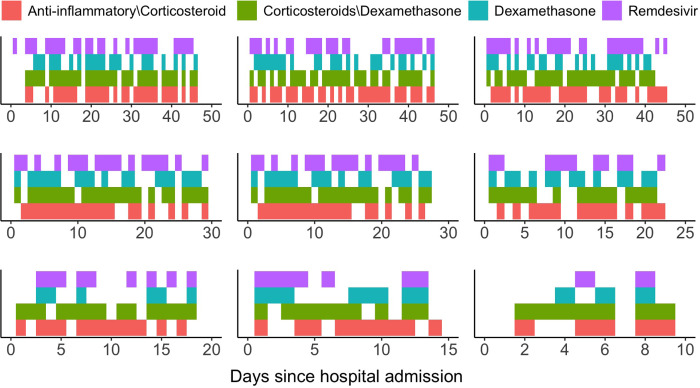
Treatment processes for nine randomly selected patients visualized by heat maps. Colors indicate remaining on treatment. Lack of color corresponds to being switched off treatment.

The complex nature of COVID-19 treatments, owing to differential physician preferences and variability of treatment choices attributable to evolving clinical guidelines, poses three major challenges for statistical analysis of observational data that cannot be easily addressed by existing longitudinal causal inference methods. First, treatment is not randomly allocated and the treatment status over time may depend upon the evolving patient- and disease-specific covariates. Second, the measurement time points during the follow-up are irregularly spaced. Third, there is more than one treatment under consideration. Patients can be simultaneously prescribed to various treatment combinations, or can be switched to a different treatment. Figure 1 illustrates the observed treatment trajectories for nine randomly selected patients during their hospital stays.

While previous work has shown that a continuous-time marginal structural model is effective in addressing time-varying confounding and provides consistent causal effect estimators (Johnson and Tsiatis, 2005; Saarela and Liu, 2016; Hu et al., 2018; Hu and Hogan, 2019; Ryalen et al., 2020), the development has been restricted to a single longitudinal treatment and therefore may not be directly applicable. We consider a joint marginal structural model to accommodate multiple longitudinal treatments in continuous time. To estimate causal parameters in the joint marginal structural model, we derive a novel set of continuous-time stabilized inverse probability weights by casting each treatment process as a counting process for recurrent events, allowing for discontinuous intervals of eligibility. In addition, we propose to use machine learning and smoothing techniques designed for censored survival data to estimate such complex weights. Through simulations, we demonstrate that our approach provides valid causal effect estimates and can considerably alleviate the consequence of unstable inverse probability weights under parametric formulations. We further undertake a detailed analysis of a large longitudinal registry data of clinical management and patient outcomes to investigate the comparative effectiveness of multiple COVID-19 treatments on patient survival.

## 2. Joint Marginal Structural Survival Model

### 2.1 Notation and set up

We consider a longitudinal observational study with multiple treatments and a right-censored survival outcome. Denote *t* as the time elapsed from study entry (e.g., hospital admission), *t*^*o*^ the maximum follow-up time, and J a collection of time points on the interval [0, *t*^*o*^]. Suppose each individual has a *p*-dimensional covariate process {L(t):t∈J}, some elements of which may be time-varying; by definition, the time-fixed elements of *L*(*t*) are constant over J. Let *T* denote time to an outcome event of interest such as death, with $\left\{N^{T}(t):t\in J\right\}$ as its associated zero-one counting process. We consider *W* different medication classes (treatments), whose separate and joint causal effects on patient survival are of interest. We use *A*_*w*_(*t*) to denote the assignment of treatment w∈W={1,…,W}, which can be characterised as the counting process, where we let *A*_*w*_(*t*) = 1 if an individual is treated with *w* at time *t* and *A*_*w*_(*t*) = 0 otherwise (Johnson and Tsiatis, 2005). Let *C* denote the time to censoring due to, for example, discharge or loss to follow up. We use the overbar notation to represent the history of a random variable, for example, ${\bar{A}}_{w}(t)=\left\{A_{w}(s):0⩽s⩽t\right\}$ corresponds to the history of treatment *A*_*w*_ from hospital admission up to time *t* and $\bar{L}(t)=\left\{L(s):0⩽s⩽t\right\}$ corresponds to the covariate history up to time *t*. Following the convention in the longitudinal causal inference literature (Robins et al., 2008), we assume the treatment decision is made only after observing the most recent covariate information just prior to the treatment; that is, for a given *t*, *A*_*w*_(*t*) occurs after *L*(*t*) for all *w*.

Let $T^{{\bar{a}}_{1}(t),\ldots ,{\bar{a}}_{W}(t)}$ represent the counterfactual failure time to event of interest had an individual been *assigned* treatment history $\left\{{\bar{a}}_{1}(t),{\bar{a}}_{2}(t),\ldots {\bar{a}}_{W}(t)\right\}$ rather than the *observed* treatment history $\left\{{\bar{A}}_{1}(t),{\bar{A}}_{2}(t),\ldots {\bar{A}}_{W}(t)\right\}$. Similarly, $T^{{\bar{A}}_{1}(t),\ldots ,{\bar{A}}_{W}(t)}$ represents the observed failure time to event for an individual given the observed treatment history. We similarly define $C^{{\bar{a}}_{1}(t),\ldots ,{\bar{a}}_{W}(t)}$ as the counterfactual censoring time under treatment $\left\{{\bar{a}}_{1}(t),{\bar{a}}_{2}(t),\ldots {\bar{a}}_{W}(t)\right\}$. The observed data available for drawing inferences about the distribution of potential outcomes are as follows: the observed time to outcome event is T* = *T* ∧ *C*, with the censoring indicator Δ^*T*^ = *I*(*T* ⩽ *C*). Note that both the treatment processes {*A*_*w*_(*t*), *w* = 1, …, *W*} and the covariate process $\bar{L}(t)$ are defined for all t∈J but are observed only at discrete and potentially irregularly spaced time points for each individual. For example, individual *i* may have covariates and treatment status observed at a set of discrete time points from study entry *t* = 0 to his or her last follow-up time $t_{iK_{i}}⩽t^{o}$. We denote the set of discrete time points with observed covariate and treatment information for individual *i* as $T_{i}=\left\{0,t_{i1},\ldots ,t_{iK_{i}}\right\}$, and therefore the observed covariate and treatment histories become ${\bar{L}}_{i}\left(T_{i}\right)=\left\{L(t):t\in T_{i}\right\}$ and ${\bar{A}}_{w,i}\left(T_{i}\right)=\left\{A_{w,i}(t):t\in T_{i}\right\}$.

### 2.2 Joint marginal structural model for survival outcomes

We consider a marginal structural model to estimate the joint causal effects of ${\bar{A}}_{1}(t),\ldots ,{\bar{A}}_{W}(t)$ on patient survival. The most popular model specification is a marginal structural Cox model, for its flexibility in handling baseline hazard and straightforward software implementation when used in conjunction with the stabilized inverse probability weights (Howe et al., 2012). When there is a strong concern that the proportional hazards assumption may not be satisfied across the marginal distribution of the counterfactual survival times, alternative strategies including the structural additive hazards model or accelerated failure time model can also be considered. For purposes of presenting our methodology, we focus on the marginal structural Cox model but extensions to alternative structural models are possible. For notational brevity but without loss of generality, we first consider *W* = 2 treatments. Expansion of the joint marginal structural model and weighting schema for *W* ⩾ 3 treatments is discussed in Section 3.4. Specifically, we assume $T^{{\bar{a}}_{1}(t),{\bar{a}}_{2}(t)}$ follows a marginal structural proportional hazards model of the form

(2.1) $$\lambda^{T^{{\bar{a}}_{1}(t),{\bar{a}}_{2}(t)}}(t)=\lambda_{0}(t)\text{ exp }\left\{\psi_{1}a_{1}(t)+\psi_{2}a_{2}(t)+\psi_{3}a_{1}(t)a_{2}(t)\right\},$$

where $\lambda^{T^{{\bar{a}}_{1}(t),{\bar{a}}_{2}(t)}}$ is the hazard function for $T^{{\bar{a}}_{1}(t),{\bar{a}}_{2}(t)}$ and *λ*_0_(*t*) is the unspecified baseline hazard function when treatment *A*_1_ and *A*_2_ are withheld during the study. The parameter *ψ*_1_ encodes the instantaneous effect of treatment *A*_1_ on $T^{{\bar{a}}_{1}(t),{\bar{a}}_{2}(t)}$ in terms of log hazard ratio while *A*_2_ is withheld during the study. Similarly, *ψ*_2_ corresponds to the instantaneous treatment effect for *A*_2_ in the absence of *A*_1_. The multiplicative interaction effect of *A*_1_ and *A*_2_ is captured by *ψ*_3_. In addition, the hazard function $\lambda^{T^{{\bar{a}}_{1}(t),{\bar{a}}_{2}(t)}}$ can depend on baseline covariates by elaborating model (2.1) or by using a stratified version of *λ*_0_(*t*). Model (2.1) implicitly assumes that the instantaneous treatment effect is constant in the course of follow-up. This model assumption is reasonable given that the COVID-related hospitalization is generally short and medications are prescribed for days in succession. Finally, model (2.1) is a continuous-time generalization of the discrete-time model considered by Howe et al. (2012) for estimating the joint survival effects of multiple time-varying treatments.

Model (2.1) offers two advantages for the estimation of treatment effects. First, the counterfactual survival function can be expressed as $S^{T^{{\bar{a}}_{1}(t),{\bar{a}}_{2}(t)}}\left(t\right)=\text{exp }\left\{-\int_{0}^{t}\lambda_{T^{{\bar{a}}_{1}(t),{\bar{a}}_{2}(t)}}(s)ds\right\}$. Therefore, causal contrasts can be performed based on any relevant summary measures of the counterfactual survival curves including median survival times and restricted mean survival times. Second, model (2.1) allows for the estimation of causal effects of interventions defined by varying treatment initiation timing and treatment duration. For example, an intervention may take the form of ${\bar{a}}_{1}\left(t^{†}\right)=\left\{a_{1}(s)=1,0⩽s⩽t^{†}\right\}$, representing prescribing treatment *A*_1_ until *t*^†^ (e.g., *t*^†^ = day 6). A more complex intervention strategy is $\left\{{\bar{a}}_{1}\left(t^{†}\right),{\bar{a}}_{2}\left(t^{†}\right)\right\}=\left\{a_{1}(s)=1\left(0⩽s⩽t^{†}/2\right),a_{2}(s)=1\left(t^{†}/2<s⩽t^{†}\right)\right\}$, which refers to assigning treatment *A*_1_ until *t*^†^*/*2 and then switching altogether to *A*_2_ until *t*^†^.

## 3. Estimating Structural Model Parameters in Continuous Time

To obtain a consistent estimator for *ψ* = {*ψ*_1_, *ψ*_2_, *ψ*_3_} in model (2.1) using longitudinal observational data with two treatments, we introduce the following causal assumptions and maintain them throughout the rest of the article:

(A1) *Consistency*. The observed failure times, $T=\sum_{A}T^{{\bar{a}}_{1}(t),{\bar{a}}_{2}(t)}1\left({\bar{A}}_{1}(t)={\bar{a}}_{1}(t),{\bar{A}}_{2}(t)={\bar{a}}_{2}(t)\right)$, where $A=\left\{{\bar{a}}_{1}(t),{\bar{a}}_{2}(t):a_{1}(t)\in \left\{0,1\right\},a_{2}(t)\in \left\{0,1\right\},t\in J\right\}$. Similarly for the observed censoring times, $C=\sum_{A}C^{{\bar{a}}_{1}(t),{\bar{a}}_{2}(t)}1\left({\bar{A}}_{1}(t)={\bar{a}}_{1}(t),{\bar{A}}_{2}(t)={\bar{a}}_{2}(t)\right)$. The consistency assumption implies that the observed outcome corresponds to the counterfactual outcome under a specific joint treatment trajectory $\left\{{\bar{a}}_{1}(t),{\bar{a}}_{2}(t)\right\}$ when an individual actually follows treatment $\left\{{\bar{a}}_{1}(t),{\bar{a}}_{2}(t)\right\}$. This is an extension of the consistency assumption developed with a single time-varying treatment (Robins, 1999) to two time-varying treatments.

(A2) *Conditional Exchangeability*. Alternatively referred to as *sequential randomization*, this assumption states that initiation of treatment at time *t* among those who are still alive and remain in the study is conditionally independent of the counterfactual survival time $T^{{\bar{a}}_{1}(t),{\bar{a}}_{2}(t)}$ conditional on observed treatment and covariate histories. Mathematically, let $\bar{O}\left(t^{-}\right)=\left\{\bar{L}\left(t^{-}\right),{\bar{A}}_{1}\left(t^{-}\right),{\bar{A}}_{2}\left(t^{-}\right)\right\}$ denote the observed history up to *t*^−^, then ∀ t∈J

(3.2) $$\lambda^{A_{1},A_{2}}\left(t|\bar{O}\left(t^{-}\right),T>t^{-},C>t^{-},T^{{\bar{a}}_{1}(t),{\bar{a}}_{2}(t)}\right)=\lambda^{A_{1},A_{2}}\left(t|\bar{O}\left(t^{-}\right),T>t^{-},C>t^{-}\right),$$

where $\lambda^{A_{1},A_{2}}(t)$ is the joint intensity process of the joint counting process *A*_1_(*t*) and *A*_2_(*t*). Similarly, let $\bar{O}(t)=\left\{\bar{L}(t),{\bar{A}}_{1}(t),{\bar{A}}_{2}(t)\right\}$ denote the observed history up to *t*, we assume conditional exchangeability for censoring such that ∀ t∈J,

(3.3) $$\lambda^{C}\left(t|\bar{O}(t),T>t,C>t,T^{{\bar{a}}_{1}(t),{\bar{a}}_{2}(t)}\right)=\lambda^{C}(t|\bar{O}(t),T>t,C>t),$$

where *λ*^*C*^(*t*) is the intensity process corresponding to the counting process of censoring. Our conditional exchangeability assumption is a continuous-time generalization of the usual sequential randomization assumption for the discrete-time marginal structural models (Robins, 1999; Howe et al., 2012).

(A3) *Positivity*. We assume that at any given time *t*, there is a positive probability of initiating a treatment plan, among those who are subject to initiating at least one treatment, for all configurations $\bar{O}\left(t^{-}\right):P\left\{\lambda^{A_{1},A_{2}}\left(t|\bar{O}\left(t^{-}\right),T>t^{-},C>t^{-}\right)>0\right\}=1$. For a pair of joint treatments (*A*_1_, *A*_2_), at a given time *t*, individuals with treatment status (0, 0), (0, 1) or (1, 0) are subject to “initating” at least one treatment. The treatment initiation patterns can be as follows: (0, 0) → {(0, 1), (1, 0), (1, 1)}, (0, 1) → {(1, 1),(1, 0)}, (1, 0) → {(1, 1), (0, 1)}. Treatment discontinuation (from 1 to 0), however, is not considered a stochastic process in our study, as COVID medication is typically prescribed with a specific treatment duration, e.g., treat with dexamethasone at a dose of 6 mg once daily for 10 days (RECOVERY Collaborative Group, 2021). Furthermore, because an individual cannot be at risk for receiving the same treatment once he or she is on the treatment, we only need to assume the positivity when the individual is *off* that specific treatment, i.e., at risk for initiating that treatment.

### 3.1 Framing repeated treatment initiation as recurrent events

As Figure 1 suggests, the observed treatment pattern is complex due to considerable variability in COVID treatment protocols and clinician preferences over time. Individuals may discontinue a treatment and restart the same treatment at a later time; or they may be switched altogether to another treatment. Meanwhile, patients can take more than one treatment for a period of time. Each treatment can therefore be viewed as the counting process of recurrent events, with discontinuous intervals of treatment eligibility (Andersen and Gill, 1982). Specifically, casting treatment initiation as a recurrent event process captures two distinguishing features of our observational data: (i) having received a treatment would prevent an individual from receiving the same treatment again for the time period while the individual is *on* the treatment; and (ii) after the individual was *off* the treatment, he or she would be eligible or *at risk* for re-initiating the treatment.

To formalize the treatment initiation process, we first consider a univariate treatment process $N^{A_{w}}$. We assume that the jumps of *A*_*w*_(*t*), i.e., *dA*_*w*_(*t*), is observed on certain subintervals of J only. Specifically for individual *i*, we observe the stochastic process *A*_*w*,*i*_(*t*) on a set of intervals $E_{w,i}=\cup_{j=1}^{J_{i}}\left(V_{w,ij},U_{w,ij}\right]$, where $0⩽V_{w,i1}⩽U_{w,i1}⩽\ldots ⩽V_{w,iJ_{i}}⩽U_{w,iJ_{i}}⩽t_{w,iK_{i}}$. This representation implies the following results. First, an individual can have at most *J*_*i*_ ⩾ 1 initiations of treatment *w*: if $U_{w,iJ_{i}}=t_{w,iK_{i}}$, then individual *i* has *J*_*i*_ − 1 treatment initiations; and if $U_{w,iJ_{i}}<t_{w,iK_{i}}$, then individual *i* has *J*_*i*_ treatment initiations. A special case where *J*_*i*_ = 1 and $U_{w,iJ_{i}}=t_{w,iK_{i}}$ corresponds to the situation where individual *i* is continuously eligible for treatment initiation and has not been treated with *w* during the follow-up. Second, once treatment is initiated, *A*_*w*,*i*_(*t*) is no longer stochastic until person *i* discontinues the treatment. This also suggests that the *j*th treatment initiation is observed at *U*_*w*,*ij*_. Third, *A*_*w*,*i*_(*t*) = 1 ∀ *t* ∈ (*U*_*w*,*ij*_, *V*_*w*,*i*(*j*+1)_], *j* = 1, …, *J*_*i*_ − 1. In words, treatment status is equal to one deterministically on the discontinuous intervals of ineligibility (i.e., *on* treatment period). Define a censoring or filtering process by $D_{i}^{A_{w}}(t)=I\left(t\in E_{w,i}\right)$, and the filtered counting process by $N_{iq}^{A_{w}}(t)=\int_{0}^{t}D_{i}^{A_{w}}(u)dN\;A_{w,iq}(u)$, where *q* indexes the *q*th treatment initiation.

Following Andersen et al. (1993), we assume conditional independence among occurrences of treatment initiation given all observed history, and that the set $E_{w,i}$ is defined such that $D_{i}^{A_{w}}(t)$ is predictable. The observed data with occurrences on the set $E_{w,i}$ can therefore be viewed as a *marked point process* generating the filtration $\left(F_{w,t}^{D}\right)$. Similarly, we denote the filtration generated by the counting process $\left\{A_{w}(t):t\in J\right\}$ corresponding to $E_{w,i}=J$ by $\left(F_{w,t}\right)$. We assume *A*_*w*,*iq*_(*t*) follows Aalen’s multiplicative intensity model (Aalen et al., 2008) *λ*_*w*,*iq*_(*t*, *θ*) = *α*_*iq*_(*θ*, *t*)*Y*_*w*,*iq*_(*t*), with respect to $\left(F_{t}\right)$, where *λ*_*w*,*iq*_(*t*, *θ*) is the intensity process of *A*_*w*,*iq*_(*t*), *α*_*iq*_(*θ*, *t*) is the hazard rate function parameterized by *θ*, and *Y*_*w*,*iq*_(*t*) is the at-risk function with *Y*_*w*,*iq*_(*t*) = 1 indicating person *i* is eligible just before time *t* for the *q*th initiation of treatment *w* in the interval [*t*, *t* + *dt*), and *Y*_*w*,*iq*_(*t*) = 0 indicating otherwise. It follows that the filtered counting process $N_{iq}^{A_{w}}(t)$ follows the multiplicative intensity model

(3.4) $$\lambda_{iq}^{A_{w}}(t,\theta )=\alpha_{iq}(\theta ,t)Y_{iq}^{A_{w}}(t)$$

with respect to $\left(F_{w,t}^{D}\right)$ (Andersen et al., 1993). Here, $Y_{iq}^{A_{w}}(t)=Y_{w,iq}(t)D_{i}^{A_{w}}(t)$. With two treatments, model (3.4) can be directly extended for the joint treatment initiation process as

(3.5) $$\lambda_{iq}^{A_{1},A_{2}}(t,\theta )=\alpha_{iq}(\theta ,t)Y_{iq}^{A_{1},A_{2}}(t),$$

where $Y_{iq}^{A_{1},A_{2}}(t)=Y_{(1,2),iq}(t)D_{i}^{A_{1},A_{2}}(t)$ is the at-risk process for the *q*th treatment initiation with the filtering process defined jointly by *A*_1_ and *A*_2_.

### 3.2 Derivation of the continuous-time weights

Under assumptions (A1)–(A3), a consistent estimator of *ψ* can be obtained by solving the weighted partial score equations (Hu et al., 2018),

(3.6) $$\overset{n}{\underset{i=1}{\sum }}\int_{0}^{\infty }\Omega^{A_{1},A_{2}}\left(t_{K_{i}}\right)\left\{Z\left(A_{1i},A_{2i},t\right)-{\bar{Z}}^{*}(t;\psi )\right\}dN_{i}^{T}(t)=0,$$

where $\Omega^{A_{1},A_{2}}\left(t_{K_{i}}\right)$ is the weight that corrects for potential time-varying confounding for time-varying treatments *A*_1_ and *A*_2_, *Z*(*A*_1*i*_, *A*_2*i*_, *t*)_(3×1)_ = [*A*_1*i*_(*t*), *A*_2*i*_(*t*), *A*_1*i*_(*t*)*A*_2*i*_(*t*)]^⊤^, and

(3.7) $${\bar{Z}}^{*}=\frac{\sum_{k\in R_{t}^{T}}Z\left(A_{k1},A_{k2},t\right)Y_{k}^{*T}(t)r\left(A_{k1},A_{k2},t;\psi \right)}{\sum_{k\in R_{t}^{T}}Y_{k}^{*T}(t)r\left(A_{k1},A_{k2},t;\psi \right)}$$

is a modified version of the weighted mean of *Z* over observations still at risk for the outcome event at time *t*. In equation (3.7), we define the weighted risk set indicator for outcome $Y_{i}^{*T}(t)=\Omega^{A_{1},A_{2}}\left(t_{K_{i}}\right)Y_{i}^{T}(t)$, where $Y_{i}^{T}(t)$ is the at-risk function for the outcome event, and *r*(*a*_1_, *a*_2_, *t*) = exp{*ψ*_1_*a*_1_(*t*) + *ψ*_2_(*t*)*a*_2_(*t*) + *ψ*_3_*a*_1_(*t*)*a*_2_(*t*)}.

In the discrete-time setting with non-recurrent treatment initiation, the stabilized inverse probability weights (we suppress subscript *i* for brevity) are given by Howe et al. (2012)

(3.8) $$\Omega^{A_{1},A_{2}}(t)=\left\{\prod_{\left\{k:t_{k}⩽t\right\}}\frac{P\left(A_{1}\left(t_{k}\right)=a_{1}\left(t_{k}\right)|{\bar{A}}_{1}\left(t_{k-1}\right),{\bar{A}}_{2}\left(t_{k-1}\right)\right)}{P\left(A_{1}\left(t_{k}\right)=a_{1}\left(t_{k}\right)|{\bar{A}}_{1}\left(t_{k-1}\right),{\bar{A}}_{2}\left(t_{k-1}\right),\bar{L}\left(t_{k-1}\right),T⩾t,C⩾t\right)}\right\}\times \left\{\prod_{\left\{k:t_{k}⩽t\right\}}\frac{P\left(A_{2}\left(t_{k}\right)=a_{2}\left(t_{k}\right)|{\bar{A}}_{1}\left(t_{k}\right),{\bar{A}}_{2}\left(t_{k-1}\right)\right)}{P\left(A_{2}\left(t_{k}\right)=a_{2}\left(t_{k}\right)|{\bar{A}}_{1}\left(t_{k}\right),{\bar{A}}_{2}\left(t_{k-1}\right),\bar{L}\left(t_{k-1}\right),T⩾t,C⩾t\right)}\right\},$$

where *t*_*k*_’s are a set of ordered discrete time points common to all individuals satisfying 0 = *t*_0_ < *t*_1_ < *t*_2_ < … ⩽ *t*. While $\Omega^{A_{1},A_{2}}(t)$ in (3.8) corrects for time-varying confounding by adjusting for $\bar{L}(t)$ in the weights, it requires that the time points are well aligned across all individuals. In addition, it does not accommodate the recurrent nature of complex intervention strategies as in our observational study.

We now generalize the weights developed for the discrete-time setting to a continuous-time process, which do not require the time points to be well aligned. Partition the time interval [0, *t*] into a number of small time intervals, and let *dA*_*w*_(*s*) be the increment of *A*_*w*_ over the small time interval [*s*, *s* + *ds*), ∀*s* ∈ [0, *t*]. Recall that treatment initiation, or the jumps of *A*_*w*_(*t*), *dA*_*w*_(*t*), is observed on a number of subintervals of J only. That is, conditional on history $\bar{L}(s)$, the occurrence of treatment initiation for an individual in [s,s+ds)I(s∈E) is a Bernoulli trial with outcomes *dA*_*w*_(*s*) = 1 and *dA*_*w*_(*s*) = 0. Then the *P* (*A*_*w*_(*t*_*k*_) = *a*_*w*_(*t*_*k*_)|•) in equation (3.8) can be represented by

$$D^{A_{w}}(s){\left\{P\left(dA_{w}(s)=1|•\right)\right\}}^{dA_{w}(s)}{\left\{P\left(dA_{w}(s)=0|•\right)\right\}}^{1-dA_{w}(s)},$$

which takes the form of the individual partial likelihood for the filtered counting process $\left\{D^{A_{w}}(s)A_{w}(s):0⩽s⩽t\right\}$. When the number of time intervals in [0, *t*] increases and *ds* approaches zero, the finite product over the number of time intervals of the individual partial likelihood will approach a product integral (Aalen et al., 2008), given by

(3.9) $$\overset{t}{\underset{0}{\prod }}{\left\{D^{A_{w}}(s)\lambda^{A_{w}}(s|•)ds\right\}}^{dA_{w}(s)}{\left\{D^{A_{w}}(s)\left(1-\lambda^{A_{w}}(s|•)ds\right)\right\}}^{1-dA_{w}(s)}\;=\left[\overset{t}{\underset{0}{\prod }}{\left\{D^{A_{w}}(s)\lambda^{A_{w}}(s|•)\right\}}^{\Delta A_{w}(s)}\right]\text{ exp }\left\{-\int_{0}^{t}D^{A_{w}}(s)\lambda^{A_{w}}(s|•)ds,\right\}$$

where Δ*A*_*w*_(*t*) = *A*_*w*_(*t*)−*A*_*w*_(*t*^−^). For individual *i*, both factors in (3.9) need to be evaluated with respect to the individual’s filtered counting process $\left\{N_{iq}^{A_{w}}(t):0⩽t⩽t_{K_{i}},q=1,\ldots ,Q_{w,i}\right\}$, with the first quantity being equal to the finite product over the jump times and the second quantity being the survival function for treatment initiation. As described in Section 3.1, the number of treatment initiations for individual *i*, *Q*_*w*,*i*_ can take three values: (i) *Q*_*w*,*i*_ = 0, (ii) *Q*_*w*,*i*_ = *J*_*i*_ − 1 or (iii) *Q*_*w*,*i*_ = *J*_*i*_. Corresponding to the three cases, the quantity in (3.9) can be rewritten as

$$\text{Quantity }(3.9)=\{\begin{array}{ll} S^{A_{w}}\left(t_{K_{i}}|•\right) & \text{if }Q_{w,i}=0\\ f^{A_{w}}\left(U_{i,J_{i}-1}|•\right)\left\{S^{A_{w}}\left(V_{iJ_{i}}|•\right)-S^{A_{w}}\left(t_{iK_{i}}|•\right)\right\} & \text{if }Q_{w,i}=J_{i}-1\\ f^{A_{w}}\left(U_{iJ_{i}}|•\right) & \text{if }Q_{w,i}=J_{i}, \end{array}$$

where $S^{A_{w}}$ and $f^{A_{w}}$ are the survival and density function of the filtered counting process for treatment *A*_*w*_. Here we assume that initiations of different treatments are ordered. For example, whether to initiate *A*_2_ at time *t* is decided upon observing the treatment status *A*_1_(*t*). This suggests that the hazard function $\lambda^{A_{2}}$ is estimable by conditioning on ${\bar{A}}_{1}(t)$ and ${\bar{A}}_{2}\left(t^{-}\right)$; and the hazard function $\lambda^{A_{1}}$ is estimable by conditioning on ${\bar{A}}_{1}\left(t^{-}\right)$ and ${\bar{A}}_{2}\left(t^{-}\right)$. For exposition brevity, let ${\bar{O}}_{1}(t)=\left\{{\bar{A}}_{1}\left(t^{-}\right),{\bar{A}}_{2}\left(t^{-}\right),\bar{L}\left(t^{-}\right),T⩾t,C⩾t\right\}$, ${\bar{O}}_{2}(t)=\left\{{\bar{A}}_{1}(t),{\bar{A}}_{2}\left(t^{-}\right),\bar{L}\left(t^{-}\right),T⩾t,C⩾t\right\}$, ${\bar{O}}^{A_{1}}(t)=\left\{{\bar{A}}_{1}\left(t^{-}\right),{\bar{A}}_{2}\left(t^{-}\right),T⩾t,C⩾t\right\}$ and ${\bar{O}}^{A_{2}}(t)=\left\{{\bar{A}}_{1}(t),{\bar{A}}_{2}\left(t^{-}\right),T⩾t,C⩾t\right\}$. Putting this all together, the individual continuous-time stabilized inverse probability weight that corrects for time-varying confounding by $\bar{L}$ is given by $\Omega^{A_{1},A_{2}}(t)=\Omega^{A_{1}}(t)\Omega^{A_{2}}(t)$ with $\Omega^{A_{w}}$ being:

(3.10) $$\Omega^{A_{w}}\left(t_{K_{i}}\right)=\{\begin{array}{ll} \frac{S^{A_{w}}\left(t_{K_{i}}|{\bar{O}}^{A_{w}}\left(t_{K_{i}}\right)\right)}{S^{A_{w}}\left(t_{K_{i}}|{\bar{O}}_{w}\left(t_{K_{i}}\right)\right)} & \text{if }Q_{w,i}=0\\ \frac{f^{A_{w}}\left(U_{i,J_{i}-1}|{\bar{O}}^{A_{w}}\left(U_{i,J_{i}-1}\right)\right)\left\{S^{A_{w}}\left(V_{iJ_{i}}|{\bar{O}}^{A_{w}}\left(V_{iJ_{i}}\right)\right)-S^{A_{w}}\left(t_{iK_{i}}|{\bar{O}}^{A_{w}}\left(t_{K_{i}}\right)\right)\right\}}{f^{A_{w}}\left(U_{i,J_{i}-1}|{\bar{O}}_{w}\left(U_{i,J_{i}-1}\right)\right)\left\{S^{A_{w}}\left(V_{iJ_{i}}|{\bar{O}}_{w}\left(V_{iJ_{i}}\right)\right)-S^{A_{w}}\left(t_{iK_{i}}|{\bar{O}}_{w}\left(t_{K_{i}}\right)\right)\right\}} & \text{if }Q_{w,i}=J_{i}-1\\ \frac{f^{A_{w}}\left(U_{iJ_{i}}|{\bar{O}}^{A_{w}}\left(U_{iJ_{i}}\right)\right)}{f^{A_{w}}\left(U_{iJ_{i}}|{\bar{O}}_{w}\left(U_{iJ_{i}}\right)\right)} & \text{if }Q_{w,i}=J_{i} \end{array}$$

Turning to censoring, under the conditional exchangeability assumption (A2), the censoring process is covariate- and treatment-dependent. To correct for selection bias due to censoring, we additionally define a weight function associated with censoring,

$$\Omega^{C}\left(G_{i}\right)=\frac{S^{C}\left(G_{i}|C_{i}⩾G_{i},T_{i}⩾G_{i}\right)}{S^{C}\left(G_{i}|{\bar{A}}_{1}\left(G_{i}\right),{\bar{A}}_{2}\left(G_{i}\right),\bar{L}\left(G_{i}\right),C_{i}⩾G_{i},T_{i}⩾G_{i}\right)},$$

where *S*^*C*^ is the survival function associated with the censoring process, and

$$G_{i}=1\left(\Delta_{i}^{T}=1\right)T_{i}+1\left(\Delta_{i}^{T}=0,C_{i}>t_{K_{i}}\right)t_{K_{i}}+1\left(\Delta_{i}^{T}=0,C_{i}⩽t_{K_{i}}\right)C_{i}.$$

This leads to a final modification of the estimating equation for *ψ*,

(3.11) $$\overset{n}{\underset{i=1}{\sum }}\int_{0}^{\infty }\Omega^{A_{1},A_{2}}\Omega^{C}\left(G_{i}\right)\left\{Z\left(A_{1i},A_{2i},t\right)-{\bar{Z}}^{**}(t;\psi )\right\}dN_{i}^{T}(t)=0,$$

where ${\bar{Z}}^{**}=\frac{\sum_{k\in R_{t}^{T}}Z\left(A_{k1},A_{k2},t\right)Y_{k}^{**T}(t)r\left(A_{k1},A_{k2},t;\psi \right)}{\sum_{k\in R_{t}^{T}}Y_{k}^{**T}(t)r\left(A_{k1},A_{k2},t;\psi \right)}$ and $Y_{i}^{**T}(t)=\Omega^{C}\left(G_{i}\right)\Omega^{A_{1},A_{2}}\left(t_{K_{i}}\right)Y_{i}^{T}(t)$.

### 3.3 Estimation of the causal survival effects

We consider four ways in which the continuous-time weights $\Omega^{A_{1},A_{2}}(t)$ can be estimated: (i) fitting a usual Cox regression model for the intensity process of the counting process of treatment initiation $\left\{A_{w}(t):t\in J\right\}$, estimating the density function $f^{A_{w}}$ and survival function $S^{A_{w}}$ from the fitted model with the Nelson-Aalen estimator for the baseline intensity function, and finally calculating the weights following equation (3.10); (ii) smoothing the Nelson-Aalen estimator and in turn $f^{A_{w}}$ and $S^{A_{w}}$ estimated from the fitted Cox regression model by means of kernel functions (Ramlau-Hansen, 1983), and calculating the weights using the smoothed version of $f^{A_{w}}$ and $S^{A_{w}}$; (iii) fitting a multiplicative intensity tree-based model (Yao et al., 2020) in which the functional form of the intensity ratio for treatment initiation is flexibly captured to estimate the $f^{A_{w}}$ and $S^{A_{w}}$ and calculate the weights; (iv) smoothing the Nelson-Aalen estimator of the baseline intensity from the tree-based model and calculating the weights using the smoothed version of $f^{A_{w}}$ and $S^{A_{w}}$. Among these approaches, (i) relies on the parametric assumptions about the intensity ratio relationships between the treatment initiation process and covariate process and may be subject to model misspecification and bias for estimating causal effects. Compared to the Nelson-Aalen estimator which includes discrete jumps at event occurrences, the kernel function estimator in (ii) may help alleviate the issue of extreme or spiky weights, and has also been shown to be a consistent and asymptotically normal baseline intensity estimator (Andersen et al., 1993). Approach (iii) leverages a recent random survival forests model (Yao et al., 2020) that can accommodate time-varying covariates to mitigate the parametric assumptions and attendant biases associated with the usual Cox regression. With baseline time-fixed treatment, prior work has used similar machine learning techniques to improve propensity score weighting estimators (Lee et al., 2010) as well as to provide more accurate causal effect estimates with censored survival data (Hu et al., 2021). Finally, approach (iv) smooths the baseline intensity estimated from the survival forests for estimating the stabilized inverse probability weights, and serves as an additional step to smooth over the potentially spiky weights. In Section 4, we compare the performances of these four strategies to estimating the continuous-time weights to generate practical recommendations. In addition, the censoring weight function Ω^*C*^(*G*_*i*_) can be estimated in a similar fashion via any one of these four approaches. Additional details of kernel function smoothing in approach (ii) and random survival forests in approach (iii) are presented in Web Appendix S1.

To accommodate the time-varying covariate process and account for the recurrent nature of treatment initiation, we fit a survival model to the counting process style of data input. Each individual is represented by several rows of data corresponding to nonoverlapping time intervals of the form (start, stop]. To allow for discontinuous intervals of eligibility, when defining multiple time intervals $E_{w,i}=\cup_{j=1}^{J_{i}}\left(V_{w,ij},U_{w,ij}\right]$ on J for individual *i*, the duration of a treatment is removed from J when the individual is currently being treated and therefore no longer eligible for initiating the treatment. Finally, since our estimators for *ψ* is a solution to the weighted partial score equation (3.11), we can use the robust sandwich variance estimator to construct confidence intervals for the structural parameters. In practice, the robust sandwich variance estimator is at most conservative under the discrete-time setting (Shu et al., 2021), and we will empirically assess the accuracy of this variance estimator with continuous-time weights via simulations.

### 3.4 Extensions to more than two time-varying treatments

Although we introduce our methods with two longitudinal treatments, our approach can be extended to more than two time-varying treatments in a straightforward fashion. In theory, a *fully interacted* version of model (2.1) can be formed to include all the main effects of $a_{w}(t)\forall w\in W$ and the interactions thereof. Clinical interests and data sparsity on combinations of treatments may also be used to guide the inclusion of interaction terms into the structural model. Suppose $B=\left\{b_{1}(t),\ldots ,b_{V}(t)\right\}$ is a collection of causal interaction effects of interest, e.g., *b*_1_(*t*) = *a*_1_(*t*)*a*_2_(*t*), the general joint marginal structural proportional hazards model is

(3.12) $$\lambda^{T^{{\bar{a}}_{1}(t),\ldots ,{\bar{a}}_{W}\left(t\right)}}(t)=\lambda_{0}(t)\text{ exp }\left\{\overset{W}{\underset{w=1}{\sum }}\psi_{1w}a_{w}(t)+\overset{V}{\underset{v=1}{\sum }}\psi_{2v}b_{v}(t)\right\},$$

where *ψ*_1*w*_’s and *ψ*_2*v*_’s capture the causal main and interaction effects on the counterfactual hazard function. A consistent estimator of *ψ* = {*ψ*_11_, …, *ψ*_*iW*_, *ψ*_21_, …, *ψ*_2*V*_} can be obtained by solving the general form of the estimating equation

(3.13) $$\overset{n}{\underset{i=1}{\sum }}\int_{0}^{\infty }\Omega^{A_{1},\ldots ,A_{W}}\Omega^{C}\left(G_{i}\right)\left\{Z\left(A_{1i},\ldots ,A_{Wi},t\right)-{\bar{Z}}^{**}(t;\psi )\right\}dN_{i}^{T}(t)=0,$$

where *Z*(*A*_1*i*_, …, *A*_*Wi*_, *t*) is a vector of length *W* + *J* representing the time-varying treatment status *A*_*w*_(*t*) and multiplicative terms of the treatment status $A_{v}(t)A_{v^{\prime }}(t),{\bar{Z}}^{**}$ is evaluated using weighted risk set indicators $Y_{i}^{**T}(t)=\Omega^{C}\left(G_{i}\right)\Omega^{A_{1},\ldots ,A_{W}}\left(t_{K_{i}}\right)Y_{i}^{T}(t)$. The joint treatment weights $\Omega^{A_{1},\ldots ,A_{W}}\left(t_{K_{i}}\right)$ can be estimated by assuming a specific order in which treatments are initiated and calculating the weights using appropriate history information ${\bar{O}}_{w}(t)$ and ${\bar{O}}^{A_{w}}(t)$, similar as described in Section 3.2. The estimation of the censoring weights Ω^*C*^(*G*_*i*_) also follows the same strategy outlined in Section 3.2 with two longitudinal treatments.

## 4. Simulation Study

### 4.1 Simulation design

We carry out simulations to investigate the finite-sample properties of the proposed weight estimators for the marginal structure Cox model parameters. We simulate data compatible with the marginal structural Cox model by generating and relating data adhering to the structural nested accelerated failure time (SNAFT) model (Young et al., 2008). A general representation of a SNAFT model for time-varying treatment *a* is (Hernán et al., 2005)

$$T^{\bar{0}}=\int_{0}^{T^{\bar{a}}}\text{exp }\left[\psi_{\text{aft}}a(t)\right],$$

where $T^{\bar{0}}$ is the counterfactual failure time under no treatment. Robins (1992) developed a simulation algorithm to generate data adhering to the SNAFT model under the discrete-time version of the identifying assumptions (A1)–(A3) in Section 3. Young et al. (2008) showed that, under the same identifying assumptions, data adhering to a marginal structural Cox model of the form

$$\lambda^{T^{\bar{a}}}(t)=\lambda_{0}(t)\text{ exp }\left[\psi_{\text{msm}}a(t)\right]$$

can be simulated from a SNAFT model with *ψ*_aft_ = *ψ*_msm_ by adding an additional quantity to the term exp[*ψ*_aft_*a*(*t*)]. In particular when $T^{\bar{0}}$ has an exponential distribution, the additional quantity is zero, hence the structural nested AFT model simulation algorithm (Robins, 1992) can be used to appropriately simulate data compatible with the marginal structural cox model under complex time-varying data structures. Building on these previous works, we extend the simulation algorithm described in Karim et al. (2017) to generate data from the joint marginal structural Cox model, while allowing for multiple time-varying treatments with discontinuous intervals of treatment eligibility and for both continuous and discrete time-varying confounders.

Throughout we simulate an observational study with *n* = 1000 patients and two time-varying treatments *A*_1_(*t*) and *A*_2_(*t*). We assume $\bar{L}(t)$ is appropriately summarized by a continuous time-varying confounding variable *L*_1_(*t*) and a binary time-varying confounding variable *L*_2_(*t*). The simulation algorithm includes two steps. In step (1), we consider nonlinear terms for the continuous variable *L*_1_(*t*_*k*_) and the interaction term *A*_1_(*t*_*k*−1_)×*L*_1_(*t*_*k*_), *A*_2_(*t*_*k*_)×*L*_1_(*t*_*k*_), *A*_1_(*t*_*k*−1_)×*L*_2_(*t*_*k*_) and *A*_2_(*t*_*k*_) × *L*_2_(*t*_*k*_) in the true treatment decision model. In particular, past treatment status {*A*_1_(*t*_*k*−1_), *A*_2_(*t*_*k*_)} is a predictor of *L*(*t*_*k*_), which then predicts future treatment exposure {*A*_1_(*t*_*k*_), *A*_2_(*t*_*k*+1_)} as well as future failure status *Y*(*t*_*k*+1_) via $1/\text{log}\left(T^{\bar{0}}\right)$ (Karim et al., 2017). Therefore, *L*(*t*_*k*_) is a time-dependent confounder affecting both the future treatment choices and counterfactual survival outcomes. The simulation of treatment initiation is placed in the recurrent events framework. Once treatment is initiated at time *t*_*k*_, treatment duration following initiation is simulated from a zero-truncated Poisson distribution. In step (1), we generate a longitudinal data set with 100 × 1000 observations (100 aligned measurement time points for each of *n* = 1000 individuals). In step (2), we randomly discard a proportion of follow-up observations for a randomly selected subset of individuals (Lin et al., 2004); and in the resulting data set, the individuals will have varying number of follow-up measurement time points, which are also irregularly spaced. Web Appendix S2 provides the full pseudo-code for simulating data under the marginal structural Cox model with two time-varying treatments.

Our simulation parameters are chosen so that the simulated data possess similar characteristics to those observed in the COVID-19 data set. The treatments *A*_1_ and *A*_2_ are simulated to resemble dexamethasone and remdesivir such that: (i) about 20% patients did not take any of the anti-viral and anti-inflammatory medications aimed at treating COVID-19; (ii) among those who were treated, 62% took dexamethasone only, 25% took remdesivir only and 13% took both (either concurrently or with treatment switching); (iii) the number of initiations for both treatments ranges from 0 to 4 with the average medication duration about 5 days. The values of treatment effect parameters *ψ*_1_ and *ψ*_2_ were set to yield a 6.7% mortality proportion among those who received dexamethasone and a 4.9% mortality proportion in those treated with remedesivir.

### 4.2 Comparison of methods

We conduct two sets of simulations to investigate the finite-sample performance of our proposed joint marginal structural survival model in continuous time (JMSSM-CT). First, we compare how accurate the four weight estimators described in Section 3.3 estimate the structural parameter *ψ*. Second, we use the best weight estimator, suggested by the first set of simulation, for JMSSM-CT, and compare it with the joint marginal structural model that requires aligned discrete time points (JMSM-DT). To ensure an objective comparison, we use the random forests (Yao et al., 2020) and adapt it into our proposed recurrent events framework to estimate the weights for JMSM-DT. In addition, we implement both JMSSM-CT and JMSM-DT on the “rectangular” simulation data with 100 aligned time points for each individual and on the “ragged” data with irregular observational time points. The performance on the rectangular data will be considered as the benchmark performance, based on which we will assess the relative accuracy of JMSSM-CT and JMSM-DT when estimating the structural parameters with the “ragged” data.

### 4.3 Performance characteristics

**Fig. 2.**
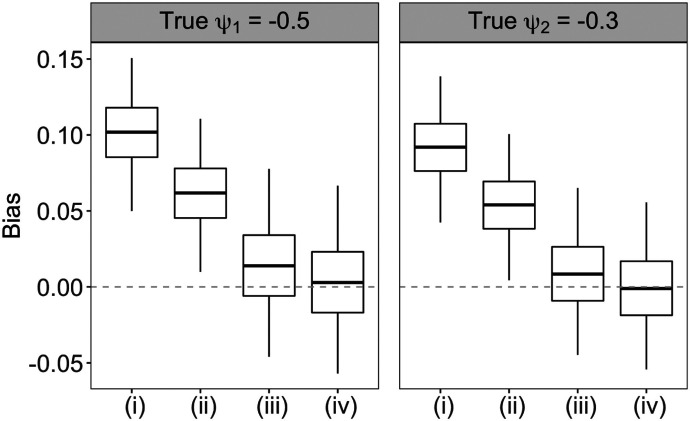
Biases in the estimates of *ψ*_1_ and *ψ*_2_ among 250 data replications using four approaches to estimate the weights as described in Section 3.4. Approach (i) uses main-effects Cox regression model and Nelson-Aalen estimator for baseline intensity. Approach (ii) uses kernel function smoothing of the Nelson-Aalen estimator in approach (i). Approach (iii) uses a survival forests model that accommodates time-varying covariates and Nelson-Aalen estimator for baseline intensity. Approach(iv) uses kernel function smoothing of the Nelson-Aalen estimator in approach (iii).

To assess the performance of each method, we simulate 250 observational data sets using the above approach, and evaluate the absolute bias, root mean squared error (RMSE) and covarage probability (CP) for estimating the *ψ*. The CP is evaluated on normality-based confidence intervals with the robust sandwich variance estimator. Figure 2 suggests that the weight estimator (iv) using both the flexible tree-based survival model and kernel function estimator of the treatment initiation intensity yielded the lowest biases in estimating both *ψ*_1_ and *ψ*_2_. By contrast, the weight estimator (i) via the usual main-effects Cox regression model along with the Nelson-Aalen baseline intensity estimator produced the largest estimation bias. Applying the kernel function smoothing to the Nelson-Aalen estimator led to bias reduction for both the Cox (approach (ii)) and tree-based survival model (approach (iv)) for the treatment process. Flexible modeling of the intensity ratio function has a larger effect in reducing the bias in structural parameter estimates than smoothing the nonparametric baseline intensity estimator. For example, compared to approach (ii), approach (iii) further reduced the mean absolute bias (MAB) in estimating ${\hat{\psi }}_{1}$ by approximately 67%. Supplementary Table 1 summarizes the MAB, RMSE and CP for the four weight estimators and similarly suggests that approach (iv) led to the smallest MAB and RSME, and provided close to nominal CP with the robust sandwich variance estimator.

The second set of simulation benchmarks the performance of JMSSM-CT versus JMSM-DT on the data with fully aligned follow-up time points and compare how much each method can recover the benchmark performance in situations where the longitudinal measurements are irregularly spaced. Table 2 displays the MAB, RMSE and CP for each of the two methods under both data settings, and Supplementary Figure 1 visualizes the distributions of biases across 250 data replications. In the rectangular data setting with fully aligned time points, compared to JMSM-DT, JMSSM-CT had similar CP but smaller MAB and RMSE. As the sparsity of longitudinal measurements increased and the time intervals became unevenly spaced, the proposed JMSSM-CT could still recover the benchmark performance; whereas the JMSM-DT had a deteriorating performance (larger MAB and RMSE and lower CP), with larger performance decline under coarser discretization of the follow-up time. Supplementary Table 2 summarizes the distribution of estimated individual time-varying weights from one random replication of the ragged data for JMSSM-CT and JMSM-DT. Overall, JMSSM-CT with the weight estimator (iv) provided the smallest maximum/minimum weight ratio (2.36/0.68) and no extreme or spiky weights.

## 5. Estimating Causal Effects of Multiple COVID-19 Treatments

We apply the proposed JMSSM-CT with the best weight estimation approach (iv) (Section 3.3) to analyze a comprehensive COVID-19 data set drawn from the Epic electronic medical records system at the Mount Sinai Medical Center, with the goal of studying the comparative effectiveness of multiple COVID-19 treatment strategies. The data set includes 11,286 de-identified unique adult patients (⩾18 years of age) who were diagnosed with COVID-19 and hospitalized within the Mount Sinai Health System between February 25, 2020 to February 26, 2021. A confirmed case of COVID-19 was defined as a positive test result from a real-time reverse-transcriptase PCR-based clinical test carried out on nasopharyngeal swab specimens collected from the patient (Wang et al., 2020). We focus on four treatment classes that are of most clinical interest: (i) remdesivir; (ii) dexamethasone; (iii) anti-inflammatory medications other than corticosteroids; and (iv) corticosteroids other than dexamethasone. Detailed definitions of the four treatment classes are provided in Supplementary Table 3. The observed treatment patterns are visualized in Figure 1; patients could be simultaneously prescribed two or more treatment strategies, or they could switch from one treatment class to another during their hospital stays.

We considered a composite outcome, ICU admission or in-hospital death, whichever occurs first. The outcome may be right censored by hospital discharge or administratively censored on *t*^*o*^= February 26, 2021, the date on which the database for the current analysis was locked. For modeling purposes, we assumed that $\bar{L}(t)$ was appropriately summarized by age, sex, race, ethnicity, D-dimer levels (the degradation product of crosslinked fibrin, reflecting ongoing activation of the hemostatic system), serum creatinine levels (a waste product that forms when creatine breaks down, indicating how well kidneys are working), whether the patient used tobacco at the time of admission, whether the patient was admitted to ICU, history of comorbidities represented by a set of binary variables: hypertension, coronary artery disease, cancer, diabetes, asthma and chronic obstructive pulmonary disease, hospital site, and patient oxygen levels (definition provided in Supplementary Table 4). The time-varying confounding variables were ICU admission, D-dimer levels, serum creatbinine level and patient oxygen levels. When fitting the joint marginal structural proportional hazards model (3.12), pairwise treatment interactions were included if there were sufficient data points supporting the joint use of the pair of treatments.

**Fig. 3.**
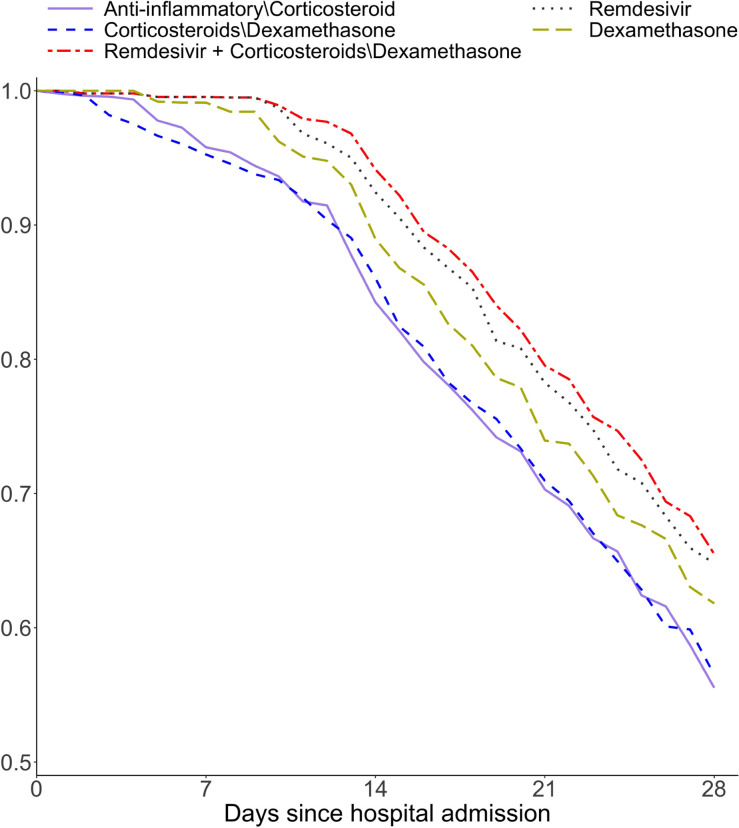
Counterfactual survival curves for each of five treatment strategies among the general COVID-19 patients. The composite outcome of ICU admission or death is used.

Using the stabilized inverse probability weights to correct for time-varying confounding and censoring, the structural model parameter estimates $\hat{\psi }$ (log hazard ratio) and the associated 95% confidence intervals are provided in Table 2. Using the parameter estimates, we further computed the counterfactual survival curves under each treatment regimen. Figure 3 presents the counterfactual survival curves for ICU admission or death, whichever occurs first, among patients with COVID-19 infection, under five treatment regimens given upon admission to hospital. Among the four separate treatment classes, remdesivir had significantly better treatment benefits followed by dexamethasone than two alternative treatment classes: anti-inflammatory medications other than corticosteroid and corticosteroids other than dexamethasone. Interestingly, remdesivir and corticosteroids other than dexamethasone had a significant treatment interaction effect suggesting additional survival benefit when they are used in combination. This is demonstrated by the highest counterfactual survival curve under the concomitant use of these two types of medications.

## 6. Discussion

Motivated by inconclusive real-world evidence for the comparative effectiveness of multiple treatment strategies for COVID-19, we have developed a joint marginal structural survival model and novel weighting schemes to address time-varying confounding and censoring in continuous time. There are three main advantages of our proposed method. First, this approach casts the complex time-varying treatment with irregular “start/stop” switches into the process of recurrent events where treatment initiation can be considered under the recurrent event framework with discontinuous intervals of eligibility. This innovative formulation enables us to address complex time-varying confounding by modeling the intensity processes of the filtered counting processes for complex time-varying treatments. Second, the proposed method is able to handle a complex longitudinal dataset on its own terms, without discretizing and artificially aligning measurement times, which would lead to less accurate and efficient treatment effect estimates, as demonstrated by our simulations. Third, modern machine learning techniques designed for censored survival data and smoothing techniques of the baseline intensity function can be used easily for with our weighting method to further improve the treatment effect estimator under conventional parametric formulations. We have also introduced a simulation algorithm that is compatible with the complex data structures of our proposed modeling framework, and demonstrated the accuracy of the proposed method for estimating causal parameters.

Our approach can be extended in the following two directions. First, we considered a joint marginal structural proportional hazards model and a tailored simulation algorithm to generate datasets of complex time-varying structures that are compatible with the proportional hazards model. It may be worthwhile to develop alternative joint marginal structural survival models such as the structural additive hazards model, and assess the robustness of different structural models for estimating counterfactual survival functions under different data generating processes. Second, we have maintained the conditional exchangeability assumption in our work, and therefore developing sensitivity analyses to capture the effects of time-varying unmeasured confounding for our model would be a worthwhile and important contribution.

## Supplementary Material

1

## Figures and Tables

**Table 1.** Comparing the proposed method JMSSM-CT in continuous time with JMSM-DT in discrete time in estimating the treatment effect *ψ* on the bases of mean absolute bias (MAB), root mean square error (RMSE) and coverage probability (CP) across 250 data replications. In the estimation of the weights, the weight esimator (iv) was used for JMSSM-CT and the random forests adapted into our recurrent events framework (Section 3.2) was used for JMSM-DT. Both methods were implemented on the “rectangular” simulation data with 100 aligned time points for each individual and on the “ragged” data with unaligned time points. With the ragged data, the follow-up time was discretized in the space of 0.5, 1 and 2 days for JMSM-DT.

Data format	Methods	*ψ*_1_			*ψ*_2_		
		MAB	RMSE	CP	MAB	RMSE	CP
Rectangular	JMSM-DT	.021	.026	.944	.019	.023	.948
	JMSSM-CT	.015	.020	.948	.014	.018	.948
Ragged	JMSM-DT (2d)	.040	.047	.660	.035	.041	.668
	JMSM-DT (1d)	.033	.041	.732	.029	.035	.738
	JMSM-DT (0.5d)	.027	.034	.801	.024	.030	.804
	JMSSM-CT	.016	.022	.952	.015	.019	.952

**Table 2.** The joint and interactive effect estimates $\hat{\psi }$ (log hazard ratio) of COVID-19 treatments and associated 95% confidence intervals (CI), using the COVID-19 dataset drawn from the Epic electronic medical records system at the Mount Sinai Medical Center. The composite outcome of in-hospital death or admission to ICU was used for the general COVID-19 patients. Time to in-hospital death was used for subpopulations of those who had never been admitted into ICU (pre-ICU) and of post-ICU patients. Confidence intervals were estimated via the robust sandwich variance estimators. “×” denotes treatment interaction and “—” indicates that the pairwise interaction was not included in the joint marginal structural survival model.

Treatment classes	$\hat{\psi }$ (95% Confidence Interval)		
	Overall	Pre-ICU	Post-ICU
Dexamethasone	−0.20(−0.35, −0.06)	−0.06(−0.71, 0.64)	−0.75(−1.42, −0.08)
Remdesivir	−0.53(−0.75, −0.31)	−0.62(−1.22, −0.02)	−0.54(−1.04, −0.04)
Corticosteroids other than dexamethasone	−0.08(−0.29, 0.19)	−0.13(−0.46, 0.21)	−0.19(−0.27, −0.03)
Anti-inflammatory medications other thancorticosteroids	−0.05(−0.56, 0.47)	−0.28(−1.02, 0.45)	−0.08(−0.89, 0.72)
Remdesivir × Corticosteroids other than dexamethasone	−0.74(−0.95, −0.52)	—	−0.71(−1.38, −0.04)
Dexamethasone × Corticosteroids other than dexamethasone	—	—	−1.13(−1.78, −0.46)
