# ESTIMATING THE CAUSAL EFFECTS OF MULTIPLE INTERMITTENT TREATMENTS WITH APPLICATION TO COVID-19

**Published:** 2021-09-27

**Authors:** Liangyuan Hu, Fan Li, Jiayi Ji, Himanshu Joshi, Erick Scott

**Affiliations:** 1 Department of Biostatistics and Epidemiology, Rutgers University, Piscataway, NJ 08854, U.S.A.; 2 Department of Biostatistics, Yale School of Public Health, New Haven, Connecticut 06510, U.S.A.; 3 Department of Population Health Science and Policy, Icahn School of Medicine at Mount Sinai, New York, New York 10029, U.S.A.; 4 Karius Inc., Redwood City, CA 94065, U.S.A

**Keywords:** Causal inference, Observational data, Time-varying treatments, Recurrent events, Stabilized inverse probability weights, Machine learning

## Abstract

To draw real-world evidence about the comparative effectiveness of multiple time-varying treatment regimens on patient survival, we develop a joint marginal structural proportional hazards model and novel weighting schemes in continuous time to account for time-varying confounding and censoring. Our methods formulate complex longitudinal treatments with multiple “start/stop” switches as the recurrent events with discontinuous intervals of treatment eligibility. We derive the weights in continuous time to handle a complex longitudinal dataset on its own terms, without the need to discretize or artificially align the measurement times. We further propose using machine learning models designed for censored survival data with time-varying covariates and the kernel function estimator of the baseline intensity to efficiently estimate the continuous-time weights. Our simulations demonstrate that the proposed methods provide better bias reduction and nominal coverage probability when analyzing observational longitudinal survival data with irregularly spaced time intervals, compared to conventional methods that require aligned measurement time points. We apply the proposed methods to a large-scale COVID-19 dataset to estimate the causal effects of several COVID-19 treatment strategies on in-hospital mortality or ICU admission, and provide new insights relative to findings from randomized trials.

## 1. Introduction.

The COVID-19 pandemic has been a rapidly evolving crisis challenging global health and economies. Public health experts believe that this pandemic has no true precedent in modern times. While multiple COVID-19 vaccines have been developed across the globe, no consensus has been reached on optimal clinical management of COVID-19 (Yousefi et al., 2020). The lack of evidence for effective treatment options warrants further investigation into the causal effects of multiple COVID-19 treatment strategies currently implemented in clinics. Although randomized controlled trials (RCTs) are considered as the gold standard for evaluating the efficacy of COVID-19 therapies, they are enormously expensive and time consuming, especially in a time of crisis. Stringent inclusion and exclusion criteria also limit the generalizability of RCTs to frailer populations at higher risk for severe morbidity and mortality. To overcome these challenges, we study the causal effects of COVID-19 treatment strategies on patient survival by leveraging the continuously growing observational data collected at the Mount Sinai Health System—New York City’s largest academic medical system. We focus on four commonly used medication classes that are of most clinical interest: (i) remdesivir; (ii) dexamethasone; (iii) anti-inflammatory medications other than corticosteroids; and (iv) corticosteroids other than dexamethasone.

**Fig 1.**
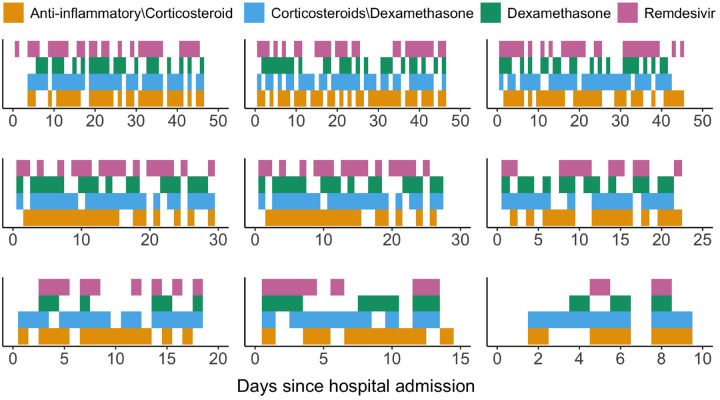
Treatment processes for nine randomly selected patients visualized by heat maps. Colors indicate remaining on treatment. Lack of color corresponds to being switched off treatment.

The complex nature of COVID-19 treatments, owing to differential physician preferences and variability of treatment choices attributable to evolving clinical guidelines, poses three major challenges for statistical analysis of observational data that cannot be easily addressed by existing longitudinal causal inference methods. First, treatment is not randomly allocated and the treatment status over time may depend upon the evolving patient- and disease-specific covariates. Second, the measurement time points during the follow-up are irregularly spaced. Third, there is more than one treatment under consideration. Patients can be simultaneously prescribed to various treatment combinations, or can be switched to a different treatment. Figure 1 illustrates the observed treatment trajectories for nine randomly selected patients during their hospital stays.

While previous work has shown that a continuous-time marginal structural model is effective in addressing time-varying confounding and provides consistent causal effect estimators (Johnson and Tsiatis, 2005; Saarela and Liu, 2016; Hu et al., 2018; Hu and Hogan, 2019; Ryalen et al., 2020), the development has been restricted to a single longitudinal treatment and therefore may not be directly applicable. We consider a joint marginal structural model to accommodate multiple longitudinal treatments in continuous time. To estimate causal parameters in the joint marginal structural model, we derive a novel set of continuous-time stabilized inverse probability weights by casting each treatment process as a counting process for recurrent events, allowing for discontinuous intervals of eligibility. In addition, we propose to use machine learning and smoothing techniques designed for censored survival data to estimate such complex weights. Through simulations, we demonstrate that our approach provides valid causal effect estimates and can considerably alleviate the consequence of unstable inverse probability weights under parametric formulations. We further undertake a detailed analysis of a large longitudinal registry data of clinical management and patient outcomes to investigate the comparative effectiveness of multiple COVID-19 treatments on patient survival.

## 2. Joint Marginal Structural Survival Model.

### 2.1. Notation and set up.

We consider a longitudinal observational study with multiple treatments and a right-censored survival outcome. Denote *t* as the time elapsed from study entry (e.g., hospital admission), *t*^*o*^ the maximum follow-up time, and 𝒥 a collection of time points on the interval [0, *t*^*o*^]. Suppose each individual has a *p*-dimensional covariate process {*L*(*t*) : *t* ∈ 𝒥}, some elements of which may be time-varying; by definition, the time-fixed elements of *L*(*t*) are constant over 𝒥. Let *T* denote time to an outcome event of interest such as death, with {*N*^*T*^ (*t*) : *t* ∈ 𝒥} as its associated zero-one counting process. We consider *W* different medication classes (treatments), whose separate and joint causal effects on patient survival are of interest. We use *A*_*w*_(*t*) to denote the assignment of treatment *w* ∈ 𝒲 = {1, …, *W*}, which can be characterized as the counting process; we let *A*_*w*_(*t*) = 1 if an individual is treated with *w* at time *t* and *A*_*w*_(*t*) = 0 otherwise (Johnson and Tsiatis, 2005).

Let *C* denote the time to censoring due to, for example, discharge or loss to follow up. We use the overbar notation to represent the history of a random variable, for example, ${\bar{A}}_{w}(t)=\left\{A_{w}(s):0\leq s\leq t\right\}$ corresponds to the history of treatment *A*_*w*_ from hospital admission up to time *t* and $\bar{L}(t)=\left\{L(s):0\leq s\leq t\right\}$ corresponds to the covariate history up to time *t*. Following the convention in the longitudinal causal inference literature (Robins, Orellana and Rotnitzky, 2008), we assume the treatment decision is made only after observing the most recent covariate information just prior to the treatment; that is, for a given *t*, *A*_*w*_(*t*) occurs after *L*(*t*) for all *w*.

Let $T^{{\bar{a}}_{1}(t),\ldots ,{\bar{a}}_{W}(t)}$ represent the counterfactual failure time to event of interest had an individual been *assigned* treatment history $\left\{{\bar{a}}_{1}(t),{\bar{a}}_{2}(t),\;\ldots \;{\bar{a}}_{W}(t)\right\}$ rather than the *observed* treatment history $\left\{{\bar{A}}_{1}(t),{\bar{A}}_{2}(t),\;\ldots \;{\bar{A}}_{W}(t)\right\}$. Similarly, $T^{{\bar{A}}_{1}(t),\ldots ,{\bar{A}}_{W}(t)}$ represents the observed failure time to event for an individual given the observed treatment history. We similarly define $C^{{\bar{a}}_{1}(t),\ldots ,{\bar{a}}_{W}(t)}$ as the counterfactual censoring time under treatment $\left\{{\bar{a}}_{1}(t),{\bar{a}}_{2}(t),\;\ldots \;{\bar{a}}_{W}(t)\right\}$. The observed data available for drawing inferences about the distribution of potential outcomes are as follows: the observed time to outcome event is T* = *T* ∧ *C*, with the censoring indicator Δ^*T*^ = *I*(*T* ≤ *C*). Note that both the treatment processes {*A*_*w*_(*t*),*w* = 1, …, *W*} and the covariate process $\bar{L}(t)$ are defined for all *t* ∈ 𝒥 but are observed only at discrete and potentially irregularly spaced time points for each individual. For example, individual *i* may have covariates and treatment status observed at a set of discrete time points from study entry *t* = 0 to his or her last follow-up time $t_{iK_{i}}\leq t^{o}$. We denote the set of discrete time points with observed covariate and treatment information for individual *i* as ^${𝒯}_{i}=\left\{0,t_{i1},\;\ldots \;,t_{iK_{i}}\right\}$^, and therefore the observed covariate and treatment histories become ${\bar{L}}_{i}\left({𝒯}_{i}\right)=\left\{L(t):t\in {𝒯}_{i}\right\}$ and ${\bar{A}}_{w,i}\left({𝒯}_{i}\right)=\left\{A_{w,i}(t):t\in {𝒯}_{i}\right\}$.

### 2.2. Joint marginal structural model for survival outcomes.

We consider a marginal structural model to estimate the joint causal effects of ${\bar{A}}_{1}(t),\;\ldots \;,{\bar{A}}_{W}(t)$ on patient survival. The most popular model specification is a marginal structural Cox model, for its flexibility in handling baseline hazard and straightforward software implementation when used in conjunction with the stabilized inverse probability weights (Howe et al., 2012). When there is a strong concern that the proportional hazards assumption may not be satisfied across the marginal distribution of the counterfactual survival times, alternative strategies including the structural additive hazards model or accelerated failure time model can also be considered. For purposes of presenting our methodology, we focus on the marginal structural Cox model but extensions to alternative structural models are possible with straightforward modifications of the weighted estimating equations. For notational brevity but without loss of generality, we first consider *W* = 2 treatments. Expansion of the joint marginal structural model and weighting schema for *W* ≥ 3 treatments is discussed in Section 3.4.

Specifically, we assume $T^{{\bar{a}}_{1}(t),{\bar{a}}_{2}(t)}$ follows a marginal structural proportional hazards model of the form

(1) $$\lambda^{T^{{\bar{a}}_{1}(t),{\bar{a}}_{2}(t)}}(t)=\lambda_{0}(t)\text{ exp }\left\{\psi_{1}a_{1}(t)+\psi_{2}a_{2}(t)+\psi_{3}a_{1}(t)a_{2}(t)\right\},$$

where $\lambda^{T^{{\bar{a}}_{1}(t),{\bar{a}}_{2}(t)}}$ is the hazard function for $T^{{\bar{a}}_{1}(t),{\bar{a}}_{2}(t)}$ and *λ*_0_(*t*) is the unspecified baseline hazard function when treatment *A*_1_ and *A*_2_ are withheld during the study. The parameter *ψ*_1_ encodes the instantaneous effect of treatment *A*_1_ on $T^{{\bar{a}}_{1}(t),{\bar{a}}_{2}(t)}$ in terms of log hazard ratio while *A*_2_ is withheld during the study. Similarly, *ψ*_2_ corresponds to the instantaneous treatment effect for *A*_2_ in the absence of *A*_1_. The multiplicative interaction effect of *A*_1_ and *A*_2_ is captured by *ψ*_3_. In addition, the hazard function $\lambda^{T^{{\bar{a}}_{1}(t),{\bar{a}}_{2}(t)}}$ can depend on baseline covariates by elaborating model (1) or by using a stratified version of *λ*_0_(*t*). Model (1) implicitly assumes that the instantaneous treatment effect is constant in the course of follow-up. This model assumption is reasonable given that the COVID-related hospitalization is generally short and medications are prescribed for days in succession. Finally, model (1) is a continuous-time generalization of the discrete-time model considered by Howe et al. (2012) for estimating the joint survival effects of multiple time-varying treatments.

Model (1) offers two advantages for the estimation of treatment effects. First, the counterfactual survival function can be expressed as

$$S^{T^{{\bar{a}}_{1}(t),{\bar{a}}_{2}(t)}}(t)=\text{exp}\left\{-\int_{0}^{t}\lambda_{T^{{\bar{a}}_{1}(t),{\bar{a}}_{2}(t)}}(s)\text{ds}\right\}.$$

Therefore, causal contrasts can be performed based on any relevant summary measures of the counterfactual survival curves including median survival times and restricted mean survival times. Second, model (1) allows for the estimation of causal effects of interventions defined by varying treatment initiation timing and treatment duration. For example, an intervention may take the form of ${\bar{a}}_{1}\left(t^{†}\right)=\left\{a_{1}(s)=1,0\leq s\leq t^{†}\right\}$, representing prescribing treatment *A*_1_ until *t*^†^ (e.g., *t*^†^ = day 6). A more complex intervention strategy is $\left\{{\bar{a}}_{1}\left(t^{†}\right),{\bar{a}}_{2}\left(t^{†}\right)\right\}=\left\{a_{1}(s)=1\left(0\leq s\leq t^{†}/2\right),a_{2}(s)=1\left(t^{†}/2<s\leq t^{†}\right)\right\}$, which refers to assigning treatment *A*_1_ until *t*^†^/2 and then switching altogether to *A*_2_ until *t*^†^.

It is worth noting that the comparative effectiveness research question under investigation in this work is fundamentally different from the problem of self-controlled case series (Farrington, 1995; Simpson et al., 2013). In a self-controlled case series, individuals act as their own controls; and the goal of such studies is to compare on-drug versus off-drug period within a single patient. Contrarily, we compare the population-level causal effects of time-varying treatment regimens, each of which is followed by and thus observed from a certain number of patients in the study population. These treatment regimens were not randomly assigned to patients. Thus, specialized causal inference techniques are needed to isolate cause-andeffect from other biasing factors, which can often be time-varying and themselves may be affected by past treatments. Because of this complexity, we motivate our methodology under the counterfactual outcomes framework rooted in the emerging literature on estimating the causal effects of time-varying treatments; also see, for example, Hernán, Brumback and Robins (2001); Robins, Orellana and Rotnitzky (2008) for such methods applied to discrete-time longitudinal observational studies, and Hu et al. (2018); Hu and Hogan (2019) for such methods applied to continuous-time longitudinal and survival data but with only one binary treatment.

## 3. Estimating Structural Model Parameters in Continuous Time.

To obtain a consistent estimator for *ψ* = {*ψ*_1_, *ψ*_2_, *ψ*_3_} in model (1) using longitudinal observational data with two treatments, we introduce the following causal assumptions and maintain them throughout the rest of the article:

(A1) *Consistency*. The observed failure times,

$$T=\sum_{𝒜}T^{{\bar{a}}_{1}(t),{\bar{a}}_{2}(t)}1\left({\bar{A}}_{1}(t)={\bar{a}}_{1}(t),{\bar{A}}_{2}(t)={\bar{a}}_{2}(t)\right),$$

where ^$𝒜=\left\{{\bar{a}}_{1}(t),{\bar{a}}_{2}(t):a_{1}(t)\in \{0,1\},a_{2}(t)\in \{0,1\},t\in 𝒯\right\}$^. Similarly for the observed censoring times,

$$C=\sum_{𝒜}C^{{\bar{a}}_{1}(t),{\bar{a}}_{2}(t)}1\left({\bar{A}}_{1}(t)={\bar{a}}_{1}(t),{\bar{A}}_{2}(t)={\bar{a}}_{2}(t)\right).$$

The consistency assumption implies that the observed outcome corresponds to the counterfactual outcome under a specific joint treatment trajectory $\left\{{\bar{a}}_{1}(t),{\bar{a}}_{2}(t)\right\}$ when an individual actually follows treatment $\left\{{\bar{a}}_{1}(t),{\bar{a}}_{2}(t)\right\}$. This is an extension of the consistency assumption developed with a single time-varying treatment (Robins, 1999) to two time-varying treatments.

(A2) *Conditional Exchangeability*. Alternatively referred to as *sequential randomization*, this assumption states that initiation of treatment at time *t* among those who are still alive and remain in the study is conditionally independent of the counterfactual survival time $T^{{\bar{a}}_{1}(t),{\bar{a}}_{2}(t)}$ conditional on observed treatment and covariate histories. Mathematically, let $\bar{𝒪}\left(t^{-}\right)=\left\{\bar{L}\left(t^{-}\right),{\bar{A}}_{1}\left(t^{-}\right),{\bar{A}}_{2}\left(t^{-}\right)\right\}$ denote the observed history up to *t*^−^, then ∀ *t* ∈ 𝒥

(2) $$\lambda^{A_{1},A_{2}}\left(t|\bar{𝒪}\left(t^{-}\right),T>t^{-},C>t^{-},T^{{\bar{a}}_{1}(t),{\bar{a}}_{2}(t)}\right)=\lambda^{A_{1},A_{2}}\left(t|\bar{𝒪}\left(t^{-}\right),T>t^{-},C>t^{-}\right),$$

where $\lambda^{A_{1},A_{2}}(t)$ is the joint intensity process of the joint counting process *A*_1_(*t*) and *A*_2_(*t*). Similarly, let $\bar{𝒪}(t)=\left\{\bar{L}(t),{\bar{A}}_{1}(t),{\bar{A}}_{2}(t)\right\}$ denote the observed history up to *t*, we assume conditional exchangeability for censoring such that ∀ *t* ∈ 𝒥,

(3) $$\lambda^{C}\left(t|\bar{𝒪}(t),T>t,C>t,T^{{\bar{a}}_{1}(t),{\bar{a}}_{2}(t)}\right)=\lambda^{C}(t|\bar{𝒪}(t),T>t,C>t),$$

where *λ*^*C*^(*t*) is the intensity process corresponding to the counting process of censoring. Our conditional exchangeability assumption is a continuous-time generalization of the usual sequential randomization assumption for the discrete-time marginal structural models (Robins, 1999; Howe et al., 2012).

(A3) *Positivity*. We assume that at any given time *t*, there is a positive probability of initiating a treatment plan, among those who are subject to initiating at least one treatment, for all configurations $\bar{𝒪}\left(t^{-}\right)$:

$$P\left\{\lambda^{A_{1},A_{2}}\left(t|\bar{𝒪}\left(t^{-}\right),T>t^{-},C>t^{-}\right)>0\right\}=1.$$

For a pair of joint treatments (*A*_1_, *A*_2_), at a given time *t*, individuals with treatment status (0, 0),(0, 1) or (1, 0) are subject to “initating” at least one treatment. The treatment initiation patterns can be as follows: (0, 0) → {(0, 1),(1, 0),(1, 1)}, (0, 1) → {(1, 1),(1, 0)}, (1, 0) → {(1, 1),(0, 1)}. Treatment discontinuation (from 1 to 0), however, is not considered a stochastic process in our study, as COVID medication is typically prescribed with a specific treatment duration, e.g., treat with dexamethasone at a dose of 6 mg once daily for 10 days (RECOVERY Collaborative Group, 2021). Furthermore, because an individual cannot be at risk for receiving the same treatment once he or she is on the treatment, we only need to assume the positivity when the individual is *off* that specific treatment, i.e., at risk for initiating that treatment.

### 3.1. Framing repeated treatment initiation as recurrent events.

As Figure 1 suggests, the observed treatment pattern is complex due to considerable variability in COVID treatment protocols and clinician preferences over time. Individuals may discontinue a treatment and restart the same treatment at a later time; or they may be switched altogether to another treatment. Meanwhile, patients can take more than one treatment for a period of time. Each treatment can therefore be viewed as the counting process of recurrent events, with discontinuous intervals of treatment eligibility (Andersen and Gill, 1982). Specifically, casting treatment initiation as a recurrent event process captures two distinguishing features of our observational data: (i) having received a treatment would prevent an individual from receiving the same treatment again for the time period while the individual is *on* the treatment; and (ii) after the individual was *off* the treatment, he or she would be eligible or *at risk* for re-initiating the treatment.

To formalize the treatment initiation process, we first consider a univariate treatment process $N^{A_{w}}$. We assume that the jumps of *A*_*w*_(*t*), i.e., *dA*_*w*_(*t*), is observed on certain subintervals of 𝒥 only. Specifically for individual *i*, we observe the stochastic process *A*_*w*,*i*_(*t*) on a set of intervals

$${ℰ}_{w,i}=\overset{J_{i}}{\underset{j=1}{\cup }}\left(V_{w,ij},U_{w,ij}\right],$$

where $0\leq V_{w,i1}\leq U_{w,i1}\leq \ldots \leq V_{w,iJ_{i}}\leq U_{w,iJ_{i}}\leq t_{w,iK_{i}}$. This representation implies the following results. First, an individual can have at most *J*_*i*_ ≥ 1 initiations of treatment *w*: if $U_{w,iJ_{i}}=t_{w,iK_{i}}$, then individual *i* has *J*_*i*_ − 1 treatment initiations; and if $U_{w,iJ_{i}}<t_{w,iK_{i}}$, then individual *i* has *J*_*i*_ treatment initiations. A special case where *J*_*i*_ = 1 and $U_{w,iJ_{i}}=t_{w,iK_{i}}$ corresponds to the situation where individual *i* is continuously eligible for treatment initiation and has not been treated with *w* during the follow-up. Second, once treatment is initiated, *A*_*w*,*i*_(*t*) is no longer stochastic until person *i* discontinues the treatment. This also suggests that the *j*th treatment initiation is observed at *U*_*w*,*ij*_. Third, we have

$$A_{w,i}(t)=1,\;\forall t\;\in \left(U_{w,ij},V_{w,i(j+1)}\right],j=1,\;\ldots \;,J_{i}-1.$$

In words, treatment status is equal to one deterministically on the discontinuous intervals of ineligibility (i.e., *on* treatment period). Define a censoring or filtering process by $D_{i}^{A_{w}}(t)=I\left(t\in {ℰ}_{w,i}\right)$, and the filtered counting process by $N_{\text{iq}}^{A_{w}}(t)=\int_{0}^{t}D_{i}^{A_{w}}(u)\text{dN}A_{w,\text{iq}}(u)$, where *q* indexes the *q*th treatment initiation.

Following Andersen et al. (1993), we assume conditional independence among occurrences of treatment initiation given all observed history, and that the set ℰ_*w*,*i*_ is defined such that $D_{i}^{A_{w}}\left(t\right)$ is predictable. The observed data with occurrences on the set ℰ_*w*,*i*_ can therefore be viewed as a *marked point process* generating the filtration $\left({ℱ}_{w,t}^{D}\right)$. Similarly, we denote the filtration generated by the counting process {*A*_*w*_(*t*) : *t* ∈ 𝒥} corresponding to ℰ_*w*,*i*_ = 𝒥 by (ℱ_*w*,*t*_). We assume *A*_*w*,*iq*_(*t*) follows Aalen’s multiplicative intensity model (Aalen, Borgan and Gjessing, 2008) *λ*_*w*,*iq*_(*t*, *θ*) = *α*_*iq*_(*θ*, *t*)*Y*_*w*,*iq*_(*t*), with respect to (ℱ_*t*_), where *λ*_*w*,*iq*_(*t*, *θ*) is the intensity process of *A*_*w*,*iq*_(*t*), *α*_*iq*_(*θ*, *t*) is the hazard rate function parameterized by *θ*, and *Y*_*w*,*iq*_(*t*) is the at-risk function with *Y*_*w*,*iq*_(*t*) = 1 indicating person *i* is eligible just before time *t* for the *q*th initiation of treatment *w* in the interval [*t*, *t* + *dt*), and *Y*_*w*,*iq*_(*t*) = 0 indicating otherwise. It follows that the filtered counting process $N_{\text{iq}}^{A_{w}}(t)$ follows the multiplicative intensity model

(4) $$\lambda_{\text{iq}}^{A_{w}}(t,\theta )=\alpha_{\text{iq}}(\theta ,t)Y_{\text{iq}}^{A_{w}}(t)$$

with respect to $\left({ℱ}_{w,t}^{D}\right)$ (Andersen et al., 1993). Here, $Y_{\text{iq}}^{A_{w}}(t)=Y_{w,\text{iq}}(t)D_{i}^{A_{w}}(t)$. With two treatments, model (4) can be directly extended for the joint treatment initiation process as

(5) $$\lambda_{\text{iq}}^{A_{1},A_{2}}(t,\theta )=\alpha_{\text{iq}}(\theta ,t)Y_{\text{iq}}^{A_{1},A_{2}}(t),$$

where $Y_{\text{iq}}^{A_{1},A_{2}}(t)=Y_{(1,2),\text{iq}}(t)D_{i}^{A_{1},A_{2}}(t)$ is the at-risk process for the *q*th treatment initiation with the filtering process defined jointly by *A*_1_ and *A*_2_.

### 3.2. Derivation of the continuous-time weights.

Under assumptions (A1)-(A3), a consistent estimator of *ψ* can be obtained by solving the weighted partial score equations (Hu et al., 2018),

(6) $$\sum_{i=1}^{n}\int_{0}^{\infty }\Omega^{A_{1},A_{2}}\left(t_{K_{i}}\right)\left\{Z\left(A_{1i},A_{2i},t\right)-{\bar{Z}}^{*}(t;\psi )\right\}dN_{i}^{T}(t)=0,$$

where $\Omega^{A_{1},A_{2}}\left(t_{K_{i}}\right)$ is the weight that corrects for potential time-varying confounding for time-varying treatments *A*_1_ and *A*_2_, *Z*(*A*_1*i*_, *A*_2*i*_, *t*)_(3×1)_ = [*A*_1*i*_(*t*), *A*_2*i*_(*t*), *A*_1*i*_(*t*)*A*_2*i*_(*t*)]^⊤^, and

(7) $${\bar{Z}}^{*}=\frac{\sum_{k\in {ℛ}_{t}^{T}}Z\left(A_{k1},A_{k2},t\right)Y_{k}^{*T}(t)r\left(A_{k1},A_{k2},t;\psi \right)}{\sum_{k\in {ℛ}_{t}^{T}}Y_{k}^{*T}(t)r\left(A_{k1},A_{k2},t;\psi \right)}$$

is a modified version of the weighted mean of *Z* over observations still at risk for the outcome event at time *t*. In equation (7), we define the weighted risk set indicator for outcome $Y_{i}^{*T}(t)=\Omega^{A_{1},A_{2}}\left(t_{K_{i}}\right)Y_{i}^{T}(t)$, where $Y_{i}^{T}(t)$ is the at-risk function for the outcome event, and *r*(*a*_1_, *a*_2_, *t*) = exp{*ψ*_1_*a*_1_(*t*) + *ψ*_2_(*t*)*a*_2_(*t*) + *ψ*_3_*a*_1_(*t*)*a*_2_(*t*)}.

In the discrete-time setting with non-recurrent treatment initiation, the stabilized inverse probability weights (we suppress subscript *i* for brevity) are given by Howe et al. (2012)

(8) $$\Omega^{A_{1},A_{2}}(t)=\left\{\prod_{\left\{k:t_{k}\leq t\right\}}\frac{P\left(A_{1}\left(t_{k}\right)=a_{1}\left(t_{k}\right)|{\bar{A}}_{1}\left(t_{k-1}\right),{\bar{A}}_{2}\left(t_{k-1}\right)\right)}{P\left(A_{1}\left(t_{k}\right)=a_{1}\left(t_{k}\right)|{\bar{A}}_{1}\left(t_{k-1}\right),{\bar{A}}_{2}\left(t_{k-1}\right),\bar{L}\left(t_{k-1}\right),T\geq t,C\geq t\right)}\right\}\times \;\left\{\prod_{\left\{k:t_{k}\leq t\right\}}\frac{P\left(A_{2}\left(t_{k}\right)=a_{2}\left(t_{k}\right)|{\bar{A}}_{1}\left(t_{k}\right),{\bar{A}}_{2}\left(t_{k-1}\right)\right)}{P\left(A_{2}\left(t_{k}\right)=a_{2}\left(t_{k}\right)|{\bar{A}}_{1}\left(t_{k}\right),{\bar{A}}_{2}\left(t_{k-1}\right),\bar{L}\left(t_{k-1}\right),T\geq t,C\geq t\right)}\right\},$$

where *t*_*k*_’s are a set of ordered discrete time points common to all individuals satisfying 0 = *t*_0_ < *t*_1_ < *t*_2_ < … ≤ *t*. While $\Omega^{A_{1},A_{2}}\left(t\right)$ in (8) corrects for time-varying confounding by adjusting for $\bar{L}(t)$ in the weights, it requires that the time points are well aligned across all individuals. In addition, it does not accommodate the recurrent nature of complex intervention strategies as in our observational study.

We now generalize the weights developed for the discrete-time setting to a continuous-time process, which do not require the time points to be well aligned. Partition the time interval [0, *t*] into a number of small time intervals, and let *dA*_*w*_(*s*) be the increment of *A*_*w*_ over the small time interval [*s*, *s* + *ds*),∀*s* ∈ [0, *t*]. Recall that treatment initiation, or the jumps of *A*_*w*_(*t*), *dA*_*w*_(*t*), is observed on a number of subintervals of 𝒥 only. That is, conditional on history $\bar{L}(s)$, the occurrence of treatment initiation for an individual in [*s*, *s* + *ds*)*I*(*s* ∈ ℰ) is a Bernoulli trial with outcomes *dA*_*w*_(*s*) = 1 and *dA*_*w*_(*s*) = 0. Then the *P* (*A*_*w*_(*t*_*k*_) = *a*_*w*_(*t*_*k*_)| •) in equation (8) can be represented by

$$D^{A_{w}}(s){\left\{P\left(dA_{w}(s)=1|•\right)\right\}}^{dA_{w}(s)}{\left\{P\left(dA_{w}(s)=0|•\right)\right\}}^{1-dA_{w}(s)},$$

which takes the form of the individual partial likelihood for the filtered counting process $\left\{D^{A_{w}}(s)A_{w}(s):0\leq s\leq t\right\}$. When the number of time intervals in [0, *t*] increases and *ds* approaches zero, the finite product over the number of time intervals of the individual partial likelihood will approach a product integral (Aalen, Borgan and Gjessing, 2008), given by

$$\prod_{0}^{t}{\left\{D^{A_{w}}(s)\lambda^{A_{w}}(s|•)ds\right\}}^{dA_{w}(s)}{\left\{D^{A_{w}}(s)\left(1-\lambda^{A_{w}}(s|•)ds\right)\right\}}^{1-dA_{w}(s)}$$

(9) $$=\left[\prod_{0}^{t}{\left\{D^{A_{w}}(s)\lambda^{A_{w}}(s|•)\right\}}^{\Delta A_{w}(s)}\right]\text{exp}\left\{-\overset{t}{\underset{0}{\int }}D^{A_{w}}(s)\lambda^{A_{w}}(s|•)\text{ds},\right\}$$

where Δ*A*_*w*_(*t*) = *A*_*w*_(*t*) − *A*_*w*_(*t*^−^). For individual *i*, both factors in (9) need to be evaluated with respect to the individual’s filtered counting process $\left\{N_{\text{iq}}^{A_{w}}(t):0\leq t\leq t_{K_{i}},q=1,\;\ldots \;,Q_{w,i}\right\}$, with the first quantity being equal to the finite product over the jump times and the second quantity being the survival function for treatment initiation. As described in Section 3.1, the number of treatment initiations for individual *i*, *Q*_*w*,*i*_ can take three values: (i) *Q*_*w*,*i*_ = 0, (ii) *Q*_*w*,*i*_ = *J*_*i*_ − 1 or (iii) *Q*_*w*,*i*_ = *J*_*i*_. Corresponding to the three cases, the quantity in (9) can be rewritten as

$$\text{Quantity }\left(9\right)=\{\begin{array}{ll} S^{A_{w}}\left(t_{K_{i}}|•\right) & \text{if }Q_{w,i}=0\\ f^{A_{w}}\left(U_{i,J_{i}-1}|•\right)\left\{S^{A_{w}}\left(V_{iJ_{i}}|•\right)-S^{A_{w}}\left(t_{iK_{i}}|•\right)\right\} & \text{if }Q_{w,i}=J_{i}-1\\ f^{A_{w}}\left(U_{iJ_{i}}|•\right) & \text{if }Q_{w,i}=J_{i}, \end{array}$$

where $S^{A_{w}}$ and $f^{A_{w}}$ are the survival and density function of the filtered counting process for treatment *A*_*w*_. Here we assume that initiations of different treatments are ordered. For example, whether to initiate *A*_2_ at time *t* is decided upon observing the treatment status *A*_1_(*t*). This suggests that the hazard function $\lambda^{A_{2}}$ is estimable by conditioning on ${\bar{A}}_{1}(t)$ and ${\bar{A}}_{2}\left(t^{-}\right)$; and the hazard function $\lambda^{A_{1}}$ is estimable by conditioning on ${\bar{A}}_{1}\left(t^{-}\right)$ and ${\bar{A}}_{2}\left(t^{-}\right)$. For exposition brevity, we define

$${\bar{𝒪}}_{1}(t)=\left\{{\bar{A}}_{1}\left(t^{-}\right),{\bar{A}}_{2}\left(t^{-}\right),\bar{L}\left(t^{-}\right),T\geq t,C\geq t\right\}\;{\bar{𝒪}}_{2}(t)=\left\{{\bar{A}}_{1}(t),{\bar{A}}_{2}\left(t^{-}\right),\bar{L}\left(t^{-}\right),T\geq t,C\geq t\right\}\;{\bar{𝒪}}^{A_{1}}(t)=\left\{{\bar{A}}_{1}\left(t^{-}\right),{\bar{A}}_{2}\left(t^{-}\right),T\geq t,C\geq t\right\}\;{\bar{𝒪}}^{A_{2}}(t)=\left\{{\bar{A}}_{1}(t),{\bar{A}}_{2}\left(t^{-}\right),T\geq t,C\geq t\right\}$$

Putting this all together, the individual continuous-time stabilized inverse probability weight that corrects for time-varying confounding by $\bar{L}$ is given by $\Omega^{A_{1},A_{2}}(t)=\Omega^{A_{1}}(t)\Omega^{A_{2}}(t)$ with $\Omega^{A_{w}}$ being:

(10) $$\Omega^{A_{w}}\left(t_{K_{i}}\right)\;=\{\begin{array}{ll} \frac{S^{A_{w}}\left(t_{K_{i}}|{\bar{𝒪}}^{A_{w}}\left(t_{K_{i}}\right)\right)}{S^{A_{w}}\left(t_{K_{i}}|{\bar{𝒪}}_{w}\left(t_{K_{i}}\right)\right)} & \text{if }Q_{w,i}=0\\ \frac{f^{A_{w}}\left(U_{i,J_{i}-1}|{\bar{𝒪}}^{A_{w}}\left(U_{i,J_{i}-1}\right)\right)\left\{S^{A_{w}}\left(V_{iJ_{i}}|{\bar{𝒪}}^{A_{w}}\left(V_{iJ_{i}}\right)\right)-S^{A_{w}}\left(t_{iK_{i}}|{\bar{𝒪}}^{A_{w}}\left(t_{K_{i}}\right)\right)\right\}}{f^{A_{w}}\left(U_{i,J_{i}-1}|{\bar{𝒪}}_{w}\left(U_{i,J_{i}-1}\right)\right)\left\{S^{A_{w}}\left(V_{iJ_{i}}|{\bar{𝒪}}_{w}\left(V_{iJ_{i}}\right)\right)-S^{A_{w}}\left(t_{iK_{i}}|{\bar{𝒪}}_{w}\left(t_{K_{i}}\right)\right)\right\}} & \text{if }Q_{w,i}=J_{i}-1\\ \frac{f^{A_{w}}\left(U_{iJ_{i}}|{\bar{𝒪}}^{A_{w}}\left(U_{iJ_{i}}\right)\right)}{f^{A_{w}}\left(U_{iJ_{i}}|{\bar{𝒪}}_{w}\left(U_{iJ_{i}}\right)\right)} & \text{if }Q_{w,i}=J_{i} \end{array}$$

Turning to censoring, under the conditional exchangeability assumption (A2), the censoring process is covariate- and treatment-dependent. To correct for selection bias due to censoring, we additionally define a weight function associated with censoring,

$$\Omega^{C}\left(G_{i}\right)=\frac{S^{C}\left(G_{i}|C_{i}\geq G_{i},T_{i}\geq G_{i}\right)}{S^{C}\left(G_{i}|{\bar{A}}_{1}\left(G_{i}\right),{\bar{A}}_{2}\left(G_{i}\right),\bar{L}\left(G_{i}\right),C_{i}\geq G_{i},T_{i}\geq G_{i}\right)},$$

where *S*^*C*^ is the survival function associated with the censoring process, and

$$G_{i}=1\left(\Delta_{i}^{T}=1\right)T_{i}+1\left(\Delta_{i}^{T}=0,C_{i}>t_{K_{i}}\right)t_{K_{i}}+1\left(\Delta_{i}^{T}=0,C_{i}\leq t_{K_{i}}\right)C_{i}.$$

This leads to a final modification of the estimating equation for *ψ*,

(11) $$\sum_{i=1}^{n}\int_{0}^{\infty }\Omega^{A_{1},A_{2}}\Omega^{C}\left(G_{i}\right)\left\{Z\left(A_{1i},A_{2i},t\right)-{\bar{Z}}^{**}(t;\psi )\right\}dN_{i}^{T}(t)=0,$$

where

$${\bar{Z}}^{**}=\frac{\sum_{k\in {ℛ}_{t}^{T}}Z\left(A_{k1},A_{k2},t\right)Y_{k}^{**T}(t)r\left(A_{k1},A_{k2},t;\psi \right)}{\sum_{k\in {ℛ}_{t}^{T}}Y_{k}^{**T}(t)r\left(A_{k1},A_{k2},t;\psi \right)}$$

and $Y_{i}^{**T}(t)=\Omega^{C}\left(G_{i}\right)\Omega^{A_{1},A_{2}}\left(t_{K_{i}}\right)Y_{i}^{T}(t)$.

### 3.3. Estimation of the causal survival effects.

We consider four ways in which the continuous-time weights $\Omega^{A_{1},A_{2}}(t)$ can be estimated: (i) fitting a usual Cox regression model for the intensity process of the counting process of treatment initiation {*A*_*w*_(*t*) : *t* ∈ 𝒥}, estimating the density function $f^{A_{w}}$ and survival function $S^{A_{w}}$ from the fitted model with the Nelson-Aalen estimator for the baseline intensity function, and finally calculating the weights following equation (10); (ii) smoothing the Nelson-Aalen estimator and in turn $f^{A_{w}}$ and $S^{A_{w}}$ estimated from the fitted Cox regression model by means of kernel functions (Ramlau-Hansen, 1983), and calculating the weights using the smoothed version of $f^{A_{w}}$ and $S^{A_{w}}$; (iii) fitting a multiplicative intensity tree-based model (Yao et al., 2020) in which the functional form of the intensity ratio for treatment initiation is flexibly captured to estimate the $f^{A_{w}}$ and $S^{A_{w}}$ and calculate the weights; (iv) smoothing the Nelson-Aalen estimator of the baseline intensity from the tree-based model and calculating the weights using the smoothed version of $f^{A_{w}}$ and $S^{A_{w}}$. Among these approaches, (i) relies on the parametric assumptions about the intensity ratio relationships between the treatment initiation process and covariate process and may be subject to model misspecification and bias for estimating causal effects. Compared to the Nelson-Aalen estimator which includes discrete jumps at event occurrences, the kernel function estimator in (ii) may help alleviate the issue of extreme or spiky weights, and has also been shown to be a consistent and asymptotically normal baseline intensity estimator (Andersen et al., 1993). Approach (iii) leverages a recent random survival forests model (Yao et al., 2020) that can accommodate time-varying covariates to mitigate the parametric assumptions and attendant biases associated with the usual Cox regression. With baseline time-fixed treatment, prior work has used similar machine learning techniques to improve propensity score weighting estimators (Lee, Lessler and Stuart, 2010) as well as to provide more accurate causal effect estimates with censored survival data (Hu, Ji and Li, 2021). Finally, approach (iv) smooths the baseline intensity estimated from the survival forests for estimating the stabilized inverse probability weights, and serves as an additional step to smooth over the potentially spiky weights. In Section 4, we compare the performances of these four strategies to estimating the continuous-time weights to generate practical recommendations. In addition, the censoring weight function Ω^*C*^(*G*_*i*_) can be estimated in a similar fashion via any one of these four approaches. Additional details of kernel function smoothing in approach (ii) and random survival forests in approach (iii) are presented in Web Appendix S1.

To accommodate the time-varying covariate process and account for the recurrent nature of treatment initiation, we fit a survival model to the counting process style of data input. Each individual is represented by several rows of data corresponding to nonoverlapping time intervals of the form (start, stop]. To allow for discontinuous intervals of eligibility, when defining multiple time intervals ${ℰ}_{w,i}=\cup_{j=1}^{J_{i}}\left(V_{w,ij},U_{w,ij}\right]$ on 𝒥 for individual *i*, the duration of a treatment is removed from 𝒥 when the individual is currently being treated and therefore no longer eligible for initiating the treatment. Finally, since our estimators for *ψ* is a solution to the weighted partial score equation (11), we can use the robust sandwich variance estimator to construct confidence intervals for the structural parameters; the details of the robust sandwich variance estimator is provided in Web Appendix S1. In practice, the robust sandwich variance estimator is at most conservative under the discrete-time setting (Shu et al., 2021), and we will empirically assess the accuracy of this variance estimator with continuous-time weights via simulations.

### 3.4. Extensions to more than two time-varying treatments.

Although we introduce our methods with two longitudinal treatments, our approach can be extended to more than two time-varying treatments in a straightforward fashion. In theory, a *fully interacted* version of model (1) can be formed to include all the main effects of *a*_*w*_(*t*) ∀*w* ∈ 𝒲 and the interactions thereof. Clinical interests and data sparsity on combinations of treatments may also be used to guide the inclusion of interaction terms into the structural model. Suppose ℬ = {*b*_1_(*t*), …, *b*_*V*_ (*t*)} is a collection of causal interaction effects of interest, e.g., *b*_1_(*t*) = *a*_1_(*t*)*a*_2_(*t*), the general joint marginal structural proportional hazards model is

(12) $$\lambda^{T^{{\bar{a}}_{1}(t),\ldots ,{\bar{a}}_{W}(t)}}(t)=\lambda_{0}(t)\text{ exp}\left\{\overset{W}{\underset{w=1}{\sum }}\psi_{1w}a_{w}(t)+\overset{V}{\underset{v=1}{\sum }}\psi_{2v}b_{v}(t)\right\},$$

where *ψ*_1*w*_’s and *ψ*_2*v*_’s capture the causal main and interaction effects on the counterfactual hazard function. A consistent estimator of *ψ* = {*ψ*_11_, …, *ψ*_*iW*_, *ψ*_21_, …, *ψ*_2*V*_} can be obtained by solving the general form of the estimating equation

(13) $$\sum_{i=1}^{n}\int_{0}^{\infty }\Omega^{A_{1},\ldots ,A_{W}}\Omega^{C}\left(G_{i}\right)\left\{Z\left(A_{1i},\;\ldots \;,A_{Wi},t\right)-{\bar{Z}}^{**}(t;\psi )\right\}dN_{i}^{T}(t)=0,$$

where *Z*(*A*_1*i*_, …, *A*_*Wi*_, *t*) is a vector of length *W* + *J* representing the time-varying treatment status *A*_*w*_(*t*) and multiplicative terms of the treatment status $A_{v}(t)A_{v^{\prime }}(t)$, ${\bar{Z}}^{**}$ is evaluated using weighted risk set indicators $Y_{i}^{**T}(t)=\Omega^{C}\left(G_{i}\right)\Omega^{A_{1},\ldots ,A_{W}}\left(t_{K_{i}}\right)Y_{i}^{T}(t)$. The joint treatment weights $\Omega^{A_{1},\ldots ,A_{W}}\left(t_{K_{i}}\right)$ can be estimated by assuming a specific order in which treatments are initiated and calculating the weights using appropriate history information ${\bar{𝒪}}_{w}(t)$ and ${\bar{𝒪}}^{A_{w}}(t)$, similar as described in Section 3.2. The estimation of the censoring weights Ω^*C*^(*G*_*i*_) also follows the same strategy outlined in Section 3.2 with two longitudinal treatments.

## 4. Simulation Study.

### 4.1. Simulation design.

We carry out simulations to investigate the finite-sample properties of the proposed weight estimators for the marginal structure Cox model parameters. We simulate data compatible with the marginal structural Cox model by generating and relating data adhering to the structural nested accelerated failure time (SNAFT) model (Young et al., 2008). A general representation of a SNAFT model for time-varying treatment *a* is (Hernán et al., 2005)

$$T^{\bar{0}}=\int_{0}^{T^{\bar{a}}}\text{exp}\left[\psi_{\text{aft}}a(t)\right],$$

where $T^{\bar{0}}$ is the counterfactual failure time under no treatment. Robins (1992) developed a simulation algorithm to generate data adhering to the SNAFT model under the discrete-time version of the identifying assumptions (A1)-(A3) in Section 3. Young et al. (2008) showed that, under the same identifying assumptions, data adhering to a marginal structural Cox model of the form

$$\lambda^{T^{\bar{a}}}(t)=\lambda_{0}(t)\text{ exp}\left[\psi_{\text{msm}}a(t)\right]$$

can be simulated from a SNAFT model with *ψ*_aft_ = *ψ*_msm_ by adding an additional quantity to the term exp[*ψ*_aft_*a*(*t*)]. In particular when $T^{\bar{0}}$ has an exponential distribution, the additional quantity is zero, hence the structural nested AFT model simulation algorithm (Robins, 1992) can be used to appropriately simulate data compatible with the marginal structural Cox model under complex time-varying data structures. Building on these previous works, we extend the simulation algorithm described in Karim, Platt and Group (2017) to generate data from the joint marginal structural Cox model, while allowing for multiple time-varying treatments with discontinuous intervals of treatment eligibility and for both continuous and discrete time-varying confounders.

Throughout we simulate an observational study with *n* = 1000 patients and two time-varying treatments *A*_1_(*t*) and *A*_2_(*t*). We assume $\bar{L}(t)$ is appropriately summarized by a continuous time-varying confounding variable *L*_1_(*t*) and a binary time-varying confounding variable *L*_2_(*t*). The simulation algorithm includes two steps. In step (1), we consider nonlinear terms for the continuous variable *L*_1_(*t*_*k*_) and the interaction term *A*_1_(*t*_*k*−1_) × *L*_1_(*t*_*k*_), *A*_2_(*t*_*k*_) × *L*_1_(*t*_*k*_), *A*_1_(*t*_*k*−1_) × *L*_2_(*t*_*k*_) and *A*_2_(*t*_*k*_) × *L*_2_(*t*_*k*_) in the true treatment decision model. In particular, past treatment status {*A*_1_(*t*_*k*−1_), *A*_2_(*t*_*k*_)} is a predictor of *L*(*t*_*k*_), which then predicts future treatment exposure {*A*_1_(*t*_*k*_), *A*_2_(*t*_*k*+1_)} as well as future failure status *Y* (*t*_*k*+1_) via $1/\text{log}\left(T^{\bar{0}}\right)$. Therefore, *L*(*t*_*k*_) is a time-dependent confounder affecting both the future treatment choices and counterfactual survival outcomes. The simulation of treatment initiation is placed in the recurrent events framework. Once treatment is initiated at time *t*_*k*_, treatment duration following initiation is simulated from a zero-truncated Poisson distribution. In step (1), we generate a longitudinal data set with 100 × 1000 observations (100 aligned measurement time points for each of *n* = 1000 individuals). In step (2), we randomly discard a proportion of follow-up observations for a randomly selected subset of individuals (Lin, Scharfstein and Rosenheck, 2004); and in the resulting data set, the individuals will have varying number of follow-up measurement time points, which are also irregularly spaced. Web Appendix S2 provides the full pseudo-code for simulating data under the marginal structural Cox model with two time-varying treatments.

Our simulation parameters are chosen so that the simulated data possess similar characteristics to those observed in the motivating COVID-19 data set. The treatments *A*_1_ and *A*_2_ are simulated to resemble dexamethasone and remdesivir such that: (i) about 20% patients did not take any of the anti-viral and anti-inflammatory medications aimed at treating COVID-19; (ii) among those who were treated, 62% took dexamethasone only, 25% took remdesivir only and 13% took both (either concurrently or with treatment switching); (iii) the number of initiations for both treatments ranges from 0 to 4 with the average medication duration about 5 days. The values of treatment effect parameters *ψ*_1_ and *ψ*_2_ were set to yield a 6.7% mortality proportion among those who received dexamethasone and a 4.9% mortality proportion in those treated with remedesivir.

### 4.2. Comparison of methods.

We conduct two sets of simulations to investigate the finite-sample performance of our proposed joint marginal structural survival model in continuous time (JMSSM-CT). First, we compare how accurate the four weight estimators described in Section 3.3 estimate the structural parameter *ψ*. Second, we use the best weight estimator, suggested by the first set of simulation, for JMSSM-CT, and compare it with the joint marginal structural model that requires aligned discrete time points (JMSM-DT). To ensure an objective comparison, we use the random forests (Yao et al., 2020) and adapt it into our proposed recurrent events framework to estimate the weights for JMSM-DT. In addition, we implement both JMSSM-CT and JMSM-DT on the “rectangular” simulation data with 100 aligned time points for each individual and on the “ragged” data with irregular observational time points. The performance on the rectangular data will be considered as the benchmark performance, based on which we will assess the relative accuracy of JMSSM-CT and JMSM-DT when estimating the structural parameters with the “ragged” data.

### 4.3. Performance characteristics.

**Fig 2.**
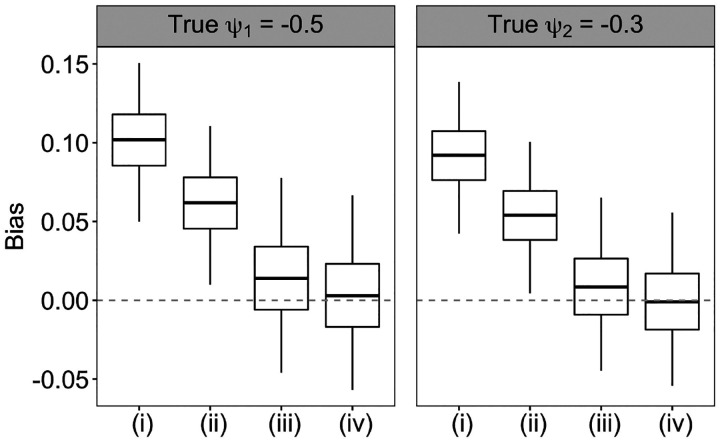
Biases in the estimates of ψ_1_ and ψ_2_ among 250 data replications using four approaches to estimate the weights as described in Section 3.3. Approach (i) uses main-effects Cox regression model and Nelson-Aalen estimator for baseline intensity. Approach (ii) uses kernel function smoothing of the Nelson-Aalen estimator in approach (i). Approach (iii) uses a survival forests model that accommodates time-varying covariates and Nelson-Aalen estimator for baseline intensity. Approach(iv) uses kernel function smoothing of the Nelson-Aalen estimator in approach (iii).

To assess the performance of each method, we simulate 250 observational data sets using the above approach, and evaluate the absolute bias, root mean squared error (RMSE) and covarage probability (CP) for estimating the *ψ*. The CP is evaluated on normality-based confidence intervals with the robust sandwich variance estimator. Figure 2 suggests that the weight estimator (iv) using both the flexible tree-based survival model and kernel function estimator of the treatment initiation intensity yielded the lowest biases in estimating both *ψ*_1_ and *ψ*_2_. By contrast, the weight estimator (i) via the usual main-effects Cox regression model along with the Nelson-Aalen baseline intensity estimator produced the largest estimation bias. Applying the kernel function smoothing to the Nelson-Aalen estimator led to bias reduction for both the Cox (approach (ii)) and tree-based survival model (approach (iv)) for the treatment process. Flexible modeling of the intensity ratio function has a larger effect in reducing the bias in structural parameter estimates than smoothing the nonparametric baseline intensity estimator. For example, compared to approach (ii), approach (iii) further reduced the mean absolute bias (MAB) in estimating ${\hat{\psi }}_{1}$ by approximately 67%. Supplementary Table 1 summarizes the MAB, RMSE and CP for the four weight estimators and similarly suggests that approach (iv) led to the smallest MAB and RSME, and provided close to nominal CP with the robust sandwich variance estimator.

The second set of simulation benchmarks the performance of JMSSM-CT versus JMSM-DT on the data with fully aligned follow-up time points and compare how much each method can recover the benchmark performance in situations where the longitudinal measurements are irregularly spaced. Table 1 displays the MAB, RMSE and CP for each of the two methods under both data settings, and Supplementary Figure 1 visualizes the distributions of biases across 250 data replications. In the rectangular data setting with fully aligned time points, compared to JMSM-DT, JMSSM-CT had similar CP but smaller MAB and RMSE. As the sparsity of longitudinal measurements increased and the time intervals became unevenly spaced, the proposed JMSSM-CT could still recover the benchmark performance; whereas the JMSM-DT had a deteriorating performance (larger MAB and RMSE and lower CP), with larger performance decline under coarser discretization of the follow-up time. Supplementary Table 2 summarizes the distribution of estimated individual time-varying weights from one random replication of the ragged data for JMSSM-CT and JMSM-DT. Overall, JMSSM-CT with the weight estimator (iv) provided the smallest maximum/minimum weight ratio (2.36/0.68) and no extreme or spiky weights.

## 5. Estimating Causal Effects of Multiple COVID-19 Treatments.

### 5.1. COVID-19 data.

We apply the proposed method JMSSM-CT to a comprehensive COVID-19 data set drawn from the Epic electronic medical records system of the Mount Sinai Medical Center, and draw causal inferences about the comparative effectiveness of multiple COVID-19 treatment strategies. The data set includes 11,286 de-identified unique adult patients (≥18 years of age) who were diagnosed with COVID-19 and hospitalized within the Mount Sinai Health System between February 25, 2020 to February 26, 2021. A confirmed case of COVID-19 was defined as a positive test result from a real-time reverse-transcriptase PCR-based clinical test carried out on nasopharyngeal swab specimens collected from the patient (Wang et al., 2020).

We focus on the comparative effectiveness of four treatment classes that are of most clinical interest: (i) remdesivir; (ii) dexamethasone; (iii) anti-inflammatory medications other than corticosteroids; and (iv) corticosteroids other than dexamethasone. We defined treatment classes by carefully reviewing the medications administered to patients. For example, the dexamethasone class includes both oral and intravenous dexamethasone; and the corticosteroids other than dexamethasone class includes oral and intravenous hydrocortisone, oral and intravenous methylprednisolone, intravenous prednisolone, and oral and intravenous prednisone. Detailed definitions of the four treatment classes are provided in Supplementary Table 3. The observed treatment patterns are visualized in Figure 1; patients could be simultaneously taking two or more treatment classes, or they could switch from one treatment class to another during their hospital stays.

Following suggestions by our clinician investigators, we assumed that the following time-fixed and time-varying confounders were sufficient to predict both treatment decision and outcome (i.e., assumption (A2) holds): age, sex, race, ethnicity, D-dimer levels (the degradation product of crosslinked fibrin, reflecting ongoing activation of the hemostatic system), serum creatinine levels (a waste product that forms when creatine breaks down, indicating how well kidneys are working), whether the patient used tobacco at the time of admission, history of comorbidity represented by a set of binary variables: hypertension, coronary artery disease, cancer, diabetes, asthma and chronic obstructive pulmonary disease, hospital site, and patient oxygen levels (definition provided in Supplementary Table 4). The time-varying confounding variables were D-dimer levels, serum creatinine level and patient oxygen levels.

**Fig 3.**
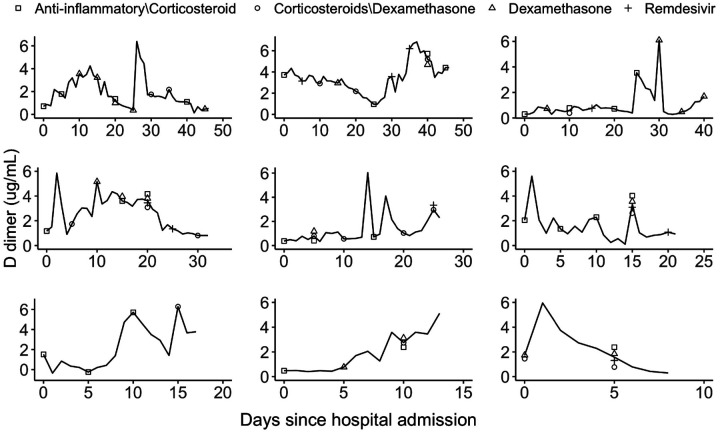
Trajectories of D-dimer levels over the course of hospital stay for 9 randomly chosen patients. Symbols represent the types of treatment classes received by a patient at a given time.

The average age of this sample population is 64.6 with a standard deviation of 18.1. About 54% of the patients were male and 46% female. The Hispanics accounted for about 26% of the patient population and the racial composition is 29% Whites, 25% Blacks, 6% Asians and 40% Other. Summary statistics for time-fixed confounders are presented in Supplementary Table 5. Time-varying confounders were measured repeatedly over the course of hospital stay. Figure 3 displays trajectories of D-dimer levels for 9 randomly chosen patients over the course of hospital stay. Supplementary Figure 2–3 show trajectories of the serum creatinine levels and patient oxygen levels. A considerable variability is observed across patients in both these time-varying measures and treatments.

We considered a composite outcome, ICU admission or in-hospital death, whichever occurs first. The outcome may be right censored by hospital discharge or administratively censored on *t*^*o*^= February 26, 2021, the date on which the database for the current analysis was locked.

### 5.2. Fitting the joint marginal structural survival models.

We implemented all four approaches discussed in Section 3.3 to estimate the time-varying weights for the JMSSM-CT model. Additionally, we discretized the time in the space of 1, 3 and 5 days, and applied the discrete time based method JMSM-DT to compare with our proposed JMSSM-CT method. The weight model included all time-fixed and time-varying confounders listed in Supplementary Table 5 and shown in Figure 3 and Supplementary Figure 2–3. No variable selection and no nonlinear transformations of the confounders were performed prior to model fitting. When fitting the joint marginal structural proportional hazards model (12), pairwise treatment interactions were included if there were sufficient data points supporting the joint use of the pair of treatments.

### 5.3. Results.

Using the stabilized inverse probability weights to correct for time-varying confounding and censoring, the structural model parameter estimates $\hat{\psi }$ (log hazard ratio) and the associated 95% confidence intervals are provided in Table 2. Echoing the findings from our simulation study (Section 4), the weight estimator (iv), using the random survival forests model and kernal function smoothing of the Nelson-Aalen estimator, produced the narrowest confidence intervals. By contrast, the weight estimator (i), using the main-effects Cox regression model and non-smoothed Nelson-Aalen estimator, led to the widest confidence intervals. As a result, using the weight estimator (iv), we observe a significant treatment benefit with dexamethasone (−.2(−.35, −.06)) and remedesivir (−.53(−.75, −.31)), and added treatment benefit if remedesivir is used in combination with corticosteroids other than dexamethasone. Using the weight estimator (i), none of the main or interactive treatment effects appeared to be statistically significant.

**Fig 4.**
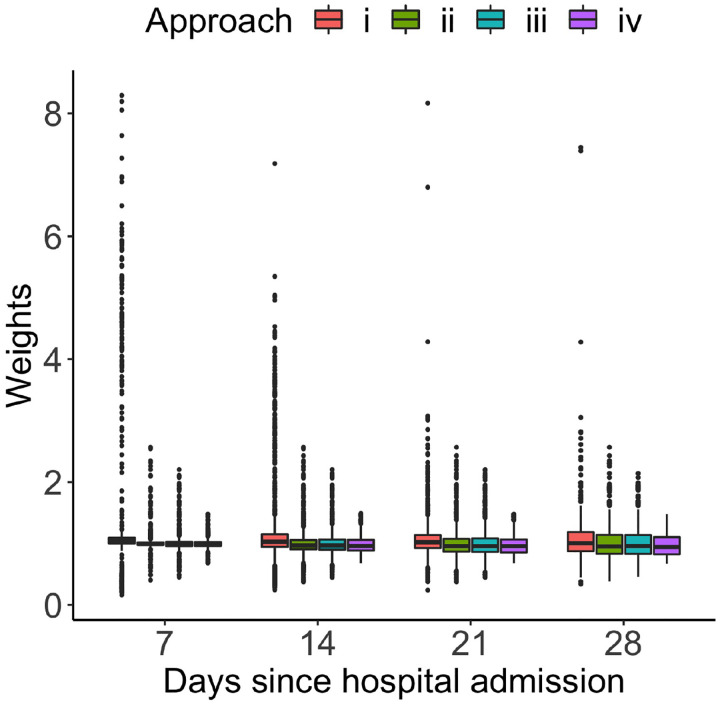
Side-by-side comparison of the time-varying weights estimated via approach (i)-(iv) described in Section 3.3.

To obtain further insights into the operating characteristics of each method, we summarize the distribution of the estimated individual weights in Table 3. As one can clearly see, the weight estimator (i) produced a substantial amount of extreme weights – the minimum of .0001 and maximum of 63. By comparison, the estimator (iv) generated no spiky weights, with the mean value of close to one. There is little difference in the weight distribution between estimator (ii) and estimator (iii), both of which mitigated the issue of extreme weights, but not to the same degree as the estimator (iv). Figure 4 shows the side-by-side comparison of the time-varying weights at 7, 14, 21 and 28 days since hospital admission estimated using the four weight estimators. The weight estimator (iv) produced no extreme weights at any of the time points. An increasing amount of extreme weights was generated when the modeling flexibility decreased or when the baseline intensity estimator was not smoothed.

Corroborating findings from our simulation study, discretizing the time can lead to the loss of information and efficiency, suggested by the width of confidence intervals of *ψ* for JMSSM-CT and JMSM-DT, shown in Table 4. The JMSSM-CT yielded the narrowest confidence intervals; whereas for JMSM-DT, the width of confidence intervals grows as the space of days for the discretization increases.

**Fig 5.**
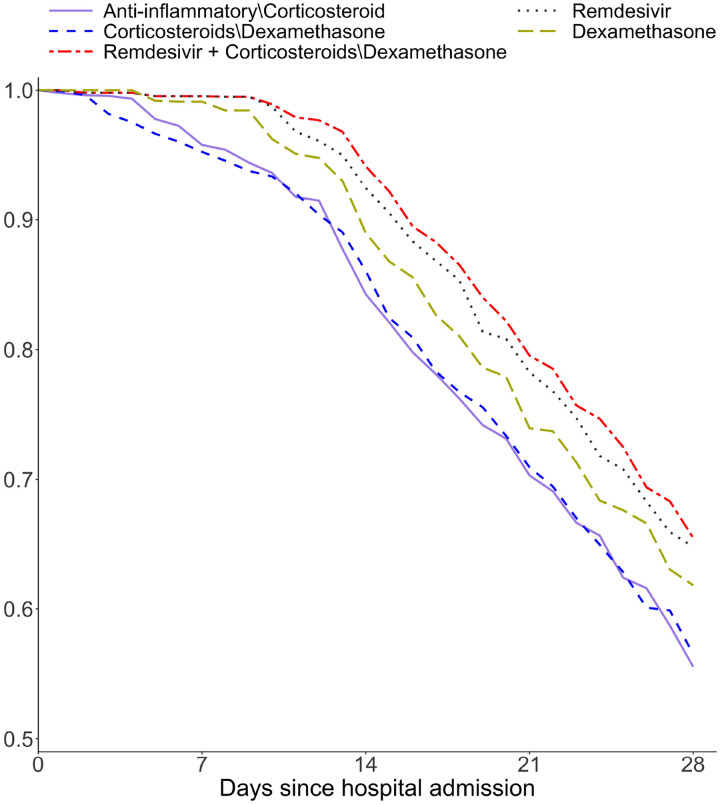
Counterfactual survival curves for each of five treatment strategies among the general COVID-19 patients. The composite outcome of ICU admission or death is used.

Using the parameter estimates $\hat{\psi }$, we further computed the counterfactual survival curves under each treatment regimen. Figure 5 presents the counterfactual survival curves, using $\hat{\psi }$ estimated by the most promising weight estimator (iv), for ICU admission or death, whichever occurs first, among patients with COVID-19 infection, under five treatment regimens given upon admission to hospital. Among the four main treatment classes, remdesivir had significantly better treatment benefits followed by dexamethasone than two alternative treatment classes: anti-inflammatory medications other than corticosteroid and corticosteroids other than dexamethasone. Interestingly, remdesivir and corticosteroids other than dexamethasone had a significant treatment interaction effect suggesting additional survival benefit when they are used in combination. This is demonstrated by the highest counterfactual survival curve under the concomitant use of these two types of medications.

### 5.4. Sensitivity analysis to assess the impact of treatment ordering.

When multiple time-varying treatments are under investigation, the analyst may face the question of the optimal treatment ordering by which the joint treatment weights $\Omega^{A_{1},\ldots ,A_{W}}\left(t_{K_{i}}\right)$ (Section 3.4) can be estimated. In our simulation and case study, we used the default decreasing order of “treatment sizes”. Note that the “treatment sizes” considered here do not refer to the sizes of mutually exclusive treatment groups, but simply the number of patients who have received the treatments at some point during the hospital stay. In our COVID-19 dataset, 7830 patients took dexamethasone (*A*_1_) at some points in time following hospital admission, 4943 took corticosteroids other than dexamethasone (*A*_2_), 4103 received remedesivir (*A*_3_), and 2844 were treated with anti-inflammatory other than corticosteroid (*A*_4_). We estimated the joint treatment weights by multiplying the sequence of conditionals,

$$\Omega^{A_{1},\ldots ,A_{4}}\left(t_{K_{i}}\right)=\Omega^{A_{1}}\left(t_{K_{i}}\right)\Omega^{A_{2}|A_{1}}\left(t_{K_{i}}\right)\Omega^{A_{3}|A_{1},A_{2}}\left(t_{K_{i}}\right)\Omega^{A_{4}|A_{1},A_{2},A_{3}}\left(t_{K_{i}}\right).$$

To evaluate whether treatment ordering can impact the causal inferences about treatment effects, we conducted a sensitivity analysis, in which we explored four choices of treatment order: decreasing order of treatment sizes, increasing order of treatment sizes and two random choices of treatment order. As demonstrated in Table 5, different treatment orders by which the joint treatment weights were estimated did not lead to appreciable or directional changes in the estimate of *ψ*. However, more efficiency was gained by using the decreasing order of treatment sizes (first conditioning on the treatment used by most patients), as suggested by the narrowest confidence intervals of *ψ*.

## 6. Discussion.

Motivated by inconclusive real-world evidence for the comparative effectiveness of multiple treatment strategies for COVID-19, we have developed a joint marginal structural survival model and novel weighting schemes to address time-varying confounding and censoring in continuous time. There are three main advantages of our proposed method. First, this approach casts the complex time-varying treatment with irregular “start/stop” switches into the process of recurrent events where treatment initiation can be considered under the recurrent event framework with discontinuous intervals of eligibility. This innovative formulation enables us to address complex time-varying confounding by modeling the intensity processes of the filtered counting processes for complex time-varying treatments. Second, the proposed method is able to handle a complex longitudinal dataset on its own terms, without discretizing and artificially aligning measurement times, which would lead to less accurate and efficient treatment effect estimates, as demonstrated by our simulations. Third, modern machine learning techniques designed for censored survival data and smoothing techniques of the baseline intensity function can be used easily for with our weighting method to further improve the treatment effect estimator under conventional parametric formulations. We have also introduced a simulation algorithm that is compatible with the complex data structures of our proposed modeling framework, and demonstrated the accuracy of the proposed method for estimating causal parameters.

Our approach can be extended in the following two directions. First, we considered a joint marginal structural proportional hazards model and a tailored simulation algorithm to generate datasets of complex time-varying structures that are compatible with the proportional hazards model. It may be worthwhile to develop alternative joint marginal structural survival models such as the structural additive hazards model, and assess the robustness of different structural models for estimating counterfactual survival functions under different data generating processes. Second, we have maintained the conditional exchangeability assumption in our work. This is a standard causal structural assumption in the literature on addressing time-varying confounding. Although untestable using the observed data, our clinician investigators supported the validity of the assumption in the COVID-19 dataset upon the review of time-varying confounders. For future research, expanding the methodology for addressing baseline unmeasured confounding (Hogan, Daniels and Hu, 2014; Hu et al., 2022) and developing sensitivity analysis approaches to capture the impact of time-varying unmeasured confounding in continuous time for our model would be a worthwhile and important contribution.

## 7. Software.

R codes to implement the proposed methods and replicate our simulation studies are provided in the GitHub page of the first author https://github.com/liangyuanhu/JMSSM-CT.

## Supplementary Material

1

## Figures and Tables

**Table 1:** Comparing the proposed method JMSSM-CT in continuous time with JMSM-DT in discrete time in estimating the treatment effect ψ on the bases of mean absolute bias (MAB), root mean square error (RMSE) and coverage probability (CP) across 250 data replications. In the estimation of the weights, the weight esimator (iv) was used for JMSSM-CT and the random forests adapted into our recurrent events framework (Section 3.2) was used for JMSM-DT. Both methods were implemented on the “rectangular” simulation data with 100 aligned time points for each individual and on the “ragged” data with unaligned time points. With the ragged data, the follow-up time was discretized in the space of 0.5, 1 and 2 days for JMSM-DT.

Data format	Methods	*ψ*_1_			*ψ*_2_		
		MAB	RMSE	CP	MAB	RMSE	CP
Rectangular	JMSM-DT	.021	.026	.944	.019	.023	.948
	JMSSM-CT	.015	.020	.948	.014	.018	.948
	JMSM-DT (2d)	.040	.047	.660	.035	.041	.668
Ragged	JMSM-DT (1d)	.033	.041	.732	.029	.035	.738
	JMSM-DT (0.5d)	.027	.034	.801	.024	.030	.804
	JMSSM-CT	.016	.022	.952	.015	.019	.952

**Table 2:** The joint and interactive effect estimates $\hat{\psi }$ (log hazard ratio) of COVID-19 treatments and associated 95% confidence intervals (CI), using the COVID-19 dataset drawn from the Epic electronic medical records system at the Mount Sinai Medical Center. The composite outcome of in-hospital death or admission to ICU was used. To estimate the weights, four approaches (i)-(iv) (Section 3.3) were used for JMSSM-CT. Confidence intervals were estimated via the robust sandwich variance estimators. “×” denotes treatment interaction.

Treatment classes	$\hat{\psi }$ (95% Confidence Interval)			
	(i)	(ii)	(iii)	(iv)
Dexamethasone	−0.02(−0.45, 0.41)	−0.15(−0.36, 0.06)	−0.19(−0.36, −0.02)	−0.20(−0.35, −0.06)
Remdesivir	−0.22(−0.61, 0.16)	−0.48(−0.76, −0.20)	−0.55(−0.78, −0.32)	−0.53(−0.75, −0.31)
Corticosteroids other than dexamethasone	0.14(−0.21, 0.49)	−0.02(−0.35, 0.31)	−0.06(−0.34, 0.24)	−0.08(−0.29, 0.19)
Anti-inflammatory medications other thancorticosteroids	0.15(−0.41, 0.72)	0.01(−0.52, 0.54)	−0.03(−0.62, 0.56)	−0.05(−0.56, 0.47)
Remdesivir × Corticosteroids other than dexamethasone	−0.22(−0.68, 0.24)	−0.67(−0.94, −0.40)	−0.69(−0.96, −0.42)	−0.74(−0.95, −0.52)

**Table 3:** The distribution of the individual time-varying weights estimated from the COVID-19 data. Four approahces (i)-(iv) described in Section 3.3 were used for the weight estimation.

Weight estimators	Distribution of estimated weights				
	Minimum	First quartile	Mean	Third quartile	Maximum
(i)	0.0001	0.172	4.443	3.367	63.112
(ii)	0.004	0.132	0.915	1.308	4.018
(iii)	0.004	0.154	1.096	1.511	5.260
(iv)	0.088	0.340	0.957	1.367	2.895

**Table 4:** Comparing the proposed JMSSM-CT with discrete-time based method JMSM-DT in estimating the joint and interactive effects $\hat{\psi }$ (log hazard ratio) of COVID-19 treatments and associated 95% confidence intervals (CI), using the COVID-19 dataset drawn from the Epic electronic medical records system if the Mount Sinai Medical Center. The composite outcome of in-hospital death or admission to ICU was used. Confidence intervals were estimated via the robust sandwich variance estimators. “×” denotes treatment interaction. The weight estimator (iv) was used for JMSSM-CT. For JMSM-DT, the follow up time was discretized in the space of 1, 3, and 5 days. The smallest unit of time in the COVID-19 data is 1 day.

Treatment orders	$\hat{\psi }$ (95% Confidence Interval)			
	JMSSM-CT	JMSM-DT (1d)	JMSM-DT (3d)	JMSM-DT (5d)
Dexamethasone	−.20(−.35,−.06)	−.22(−.39,−.05)	−.25(−.45,−.05)	−.27(−.55, .01)
Remdesivir	−.53(−.75,−.31)	−.50(−.76,−.25)	−.48(−.76,−.20)	−.42(−.74,−.10)
Corticosteroids other than dexamethasone	−.08(−.29, .19)	−.10(−.36, .21)	−.12(−.43, .22)	−.14(−.48, .24)
Anti-inflammatory medications other than corticosteroids	−.05(−.56, .47)	−.07(−.60, .48)	−.09(−.66, .49)	−.13(−.75, .51)
Remdesivir × Corticosteroids other than dexamethasone	−.74(−.95,−.52)	−.78(−1.01,−.55)	−.81(−1.07,−.56)	−.83(−1.14,−.52)

**Table 5:** The joint and interactive effect estimates $\hat{\psi }$ (log hazard ratio) of COVID-19 treatments and associated 95% confidence intervals (CI), using the COVID-19 dataset drawn from the Epic electronic medical records system of the Mount Sinai Medical Center. The composite outcome of in-hospital death or admission to ICU was used. The weight estimator (iv) (Section 3.3) was used. The joint treatment weights were estimated using four different choices of treatment order. Confidence intervals were estimated via the robust sandwich variance estimators. “×” denotes treatment interaction. A_1_ = dexamethasone. A_2_ = corticosteroids other than dexamethasone. A_3_ = remdesivir. A_4_ = anti-inflammatory medications other than corticosteroids. → denotes treatment order. For example, A_1_ → A_2_ → A_3_ → A_4_ indicates that the joint treatment weights $\Omega^{A_{1},A_{2},A_{3},A_{4}}$ are estimated by $\Omega^{A_{1},A_{2},A_{3},A_{4}}=\Omega^{A_{1}}\times \Omega^{A_{2}|A_{1}}\times \Omega^{A_{3}|A_{1},A_{2}}\times \Omega^{A_{4}|A_{1},A_{2},A_{3}}$.

Treatment orders	$\hat{\psi }$ (95% Confidence Interval)			
	*A*_1_ → *A*_2_ → *A*_3_ → *A*_4_	*A*_4_ → *A*_3_ → *A*_2_ → *A*_1_	*A*_1_ → *A*_3_ → *A*_4_ → *A*_2_	*A*_4_ → *A*_2_ → *A*_1_ → *A*_3_
Dexamethasone	−.20(−.35,−.06)	−.22(−.43,−.01)	−.23(−.38,−.08)	−.21(−.41,−.02)
Remdesivir	−.53(−.75,−.31)	−.48(−.74,−.22)	−.56(−.79,−.33)	−.50(−.74,−.26)
Corticosteroids other than dexamethasone	−.08(−.29, .19)	−.02(−.33, .29)	−.12(−.37, .13)	−.04(−.34, .26)
Anti-inflammatory medications other than corticosteroids	−.05(−.56, .47)	−.01(−.59, .57)	−.08(−.63, .47)	−.03(−.58, .51)
Remdesivir × Corticosteroids other than dexamethasone	−.74(−.95,−.52)	−.65(−.92,−.38)	−.80(−1.05,−.55)	−.68(−.94,−.44)
