# Supplementary material for: Estimating the causal effects of multiple intermittent treatments with application to COVID-19
Source: ArXiv. 2023 Aug 4:arXiv:2109.13368v4. Preprint. [Version 4] (PMC8722604)
Supplement: 1 [file NIHPP2109.13368V4-supplement-1.pdf]

arXiv:2109.13368v4 [stat.ME] 4 Aug 2023

Web-based Supplementary Materials for "Estimating the causal effects of multiple intermittent treatments with application to COVID-19" by Hu et al.

Liangyuan Hu   Jiayi Ji   Himanshu Joshi   Erick Scott   Fan Li

1 Additional technical details

1.1 The kernel function estimator

In Section 3.4, four approaches are described to estimate the stabilized time-varying inverse probability of treatment weights. The Nelson-Aalen estimator of the baseline intensity is smoothed by means of kernel functions in approach (ii) and approach (iv). For exposition brevity, consider a simple multiplicative intensity model for treatment initiation

$$\rho(t) = \rho_0(t)\text{IR}\left(\bar{L}(t), \theta\right) Y(t),$$

where $\rho_0(t)$ is the baseline intensity rate function, $\text{IR}\left(\bar{L}(t), \theta\right) > 0$ is the (time-dependent) intensity ratio function parameterized by θ (note that $\text{IR}\left(\bar{L}(t), \theta\right)$ can be nonparametrically estimated via machine learning techniques such as the random survival forests(**?**)), and $Y(t)$ is the at risk indicator. The cumulative baseline intensity is $\text{P}_0(t) = \int_0^t \rho_0(s)ds$. The kernel function estimator for $\rho_0(t)$ is derived by smoothing the increments of the Nelson-Aalen estimator of $\hat{\text{P}}_0$ as

$$\hat{\rho}_0(t) = b^{-1} \int_{\mathscr{T}} K\left(\frac{t-s}{b}\right) d\hat{\text{P}}_0(s),$$

where K is the kernel function, which is a bounded function on $[-1, 1]$ and has integral 1, and the bandwidth b is a positive parameter governing the amount of smoothness. Commonly used kernel functions include the standard normal density function $K(x) = \phi(x)$ and the Epanechnikov kernel function $K(x) = 0.75(1 - x^2),\ |x| \leq 1$. **?** shows that the kernel function estimator $\hat{\lambda}_0(t)$ is consistent and asymptotically normal provided that there exists a sequence

of positive constants $\{a_n\}$, increasing to infinity as $n \to \infty$, and that the bandwidth tends to zero more slowly than a_n^{-2} as $n \to \infty$.

Placed in the framework of recurrent event for a treatment A_w that can have multiple "start/stop" switches as described in Section 3.1, the intensity of the qth treatment initiation $\rho_q^{A_w}(t)$ is smoothed by means of kernel function estimator of the baseline intensity function $\rho_{0q}^{A_w}(t)$.

In our simulations (Section 5) and COVID-19 case study (Section 6), we used both the standard normal density $\phi(x)$ and Epanechnikov kernel functions and they yielded similar results. We presented results from the normal density kernel function. Following **?**, we chose the optimal bandwidth b as the value that minimizes the mean integrated squred error (MISE)

$$\text{MISE}(\hat{\rho}_0) = E \int_0^{t^o} \left[\hat{\rho}_0(t) - \rho(t)\right]^2 dt.$$

1.2 Random survival forests accommodating time-varying covariates

Approach (iii) uses a recent random survival forests model (**?**) that accommodates time-varying covariates to reduce the parametric assumptions about the form of the intensity ratio function required by the usual proportional intensity regression model. **?** proposed a forest method that estimates the survival function by three steps. In step (1), we reformat the data in the counting process structure, that is, for individual i, the time-varying covariate $L^{(i)}(t)$ will be represented as $L^{(i)}(t) = l_j^{(i)}$, $t \in \left[t_j^{(i)}, t_{j+1}^{(i)}\right)$, $j = 0, \ldots, J^{(i)} - 1$. Then split the individual i observation into $J^{(i)}$ pseudo subject observations: $\left(t_j^{(i)}, t_{j+1}^{(i)}, \delta_j^{(i)}, l_j^{(i)}\right)$ with left-truncated right-censored (LTRC) times $t_j^{(i)}, t_{j+1}^{(i)}$ and event indicator $\delta_j^{(i)}$ for the time interval $\left[t_j^{(i)}, t_{j+1}^{(i)}\right)$. Pool the counting process styled records from N subjects to create a list of pseudo-subjects,

$$\left\{t_l^{'}, t_{l+1}^{'}, \delta_l^{'}, l_l^{'}\right\}_{l=1}^{n}, \; n = \sum_i^N J^{(i)}. \tag{1}$$

The set of pseudo-subjects is treated as if they were independent. Step (2) is to apply the forest algorithms on the reformatted dataset given in (1), to fit a model. In step (3), given a particular stream of covariate values at the corresponding time values, a survival function estimate is constructed based on the outputs of the forest algorithms.

We now briefly describe the forest algorithms. **?** extended the relative risk forests, which combines the relative risk trees (**?**) with random forest methodology (**?**), for LTRC data by modifying the splitting criteria. The splitting criterion under the relative risk framework is to maximize the reduction in the one-step deviance between the log-likelihood of the saturated model and the maximized log-likelihood. Let $\mathcal{R}_h$ denote the set of observations that fall into the node h. Let P_0 index the baseline cumulative intensity function, φ_h represent the nonnegative relative risk of the node h, and t_l and δ_l be the time and event indicator of the lth observation $\forall l \in \mathcal{R}_h$. Given the right censored observations (t_l, δ_l), the full likelihood deviance residual for node h is defined as

$$d_h = \sum_{l \in \mathcal{R}_h} 2 \left\{ \delta_l \log \left(\frac{\delta_l}{\hat{\mathrm{P}}_0(t_l)\hat{\varphi}_h} \right) - \left(\delta_l - \hat{\mathrm{P}}_0(t_l)\hat{\varphi}_h \right) \right\}. \tag{2}$$

Two steps are needed to modify the splitting rule and obtain the deviance residual appropriate for LTRC data (**?**). First, compute the estimated cumulative intensity function $\hat{\mathrm{P}}_0(\cdot)$ based on all pseudo-subject observations. Second, replace $\hat{\mathrm{P}}_0(t_l)$ in (2) with $\hat{\mathrm{P}}_0(t'_{l+1}) - \hat{\mathrm{P}}_0(t'_l)$, and replace δ_l in (2) with δ'_l. We refer to **?** for more detailed description of the random survival forests model.

1.3 Variance estimation

Since our estimators for ψ (marginal structural proportional hazards model parameter) can be considered as solution to the weighted partial score equation, we consider the robust sandwich variance estimator as a convenience device to construct confidence intervals. The robust sand-

wich variance estimator has been considered, for example, in **?**, and **??**, and has been shown to be at most conservative under the discrete-time setting. We use this estimator for inference in conjunction with our continuous-time stabilized inverse probability weights, and formally evaluate its performance in our simulations. We briefly describe the robust sandwich variance estimator below. With the continuous-time weights, we focus on equation (11) in the main text with two treatments:

$$\sum_{i=1}^{n}\int_0^\infty \Omega^{A_1,A_2}\Omega^C(G_i)\left\{Z(A_{1i},A_{2i},t)-\frac{S^{(1)}(t;\boldsymbol{\psi})}{S^{(0)}(t;\boldsymbol{\psi})}\right\}dN_i^T(t)=0,$$

where $\Omega^{A_1,A_2}\Omega^C(G_i)$ is the weight for time-varying treatments A_1 and A_2 and censoring (in continuous time), $Z(A_{1i},A_{2i},t)_{(3\times1)}=[A_{1i}(t),A_{2i}(t),A_{1i}(t)A_{2i}(t)]^\top$, and

$$S^{(0)}(t;\boldsymbol{\psi})=\sum_{k\in\mathcal{R}_t^T}Y_k^{**T}(t)r(A_{k1},A_{k2},t;\boldsymbol{\psi})$$
$$S^{(1)}(t;\boldsymbol{\psi})=\sum_{k\in\mathcal{R}_t^T}Z(A_{k1},A_{k2},t)Y_k^{**T}(t)r(A_{k1},A_{k2},t;\boldsymbol{\psi}).$$

In the above definition, $\mathcal{R}_t^T$ refers to the collection of subjects still at risk for the outcome event at time t, $Y_k^{**T}(t)=\Omega^C(G_i)\Omega^{A_1,A_2}(t_{K_i})Y_k^T(t)$, where $Y_i^T(t)$ is the at-risk function for the outcome event, and $r(a_1,a_2,t)=\exp\{\psi_1a_1(t)+\psi_2(t)a_2(t)+\psi_3a_1(t)a_2(t)\}$. Now further define

$$S^{(2)}(t;\boldsymbol{\psi})=\sum_{k\in\mathcal{R}_t^T}Z(A_{k1},A_{k2},t)^{\otimes2}Y_k^{**T}(t)r(A_{k1},A_{k2},t;\boldsymbol{\psi}),$$

with $a^{\otimes2}=aa^\top$ for any vector a. Then the robust sandwich variance estimator takes the form $\boldsymbol{\Sigma}_0^{-1}\boldsymbol{\Sigma_1}\boldsymbol{\Sigma}_0^{-1}$ (**?**), where

$$\boldsymbol{\Sigma}_0=\sum_{i=1}^{n}\int_0^\infty \Omega^{A_1,A_2}\Omega^C(G_i)\left\{\frac{S^{(2)}(t;\boldsymbol{\psi})}{S^{(0)}(t;\boldsymbol{\psi})}-\frac{S^{(1)}(t;\boldsymbol{\psi})^{\otimes2}}{S^{(0)}(t;\boldsymbol{\psi})}\right\}dN_i^T(t),$$

and

$$\boldsymbol{\Sigma}_1=\sum_{i=1}^{n}\left[\int_0^\infty \Omega^{A_1,A_2}\Omega^C(G_i)\left\{Z(A_{1i},A_{2i},t)-\frac{S^{(1)}(t;\boldsymbol{\psi})}{S^{(0)}(t;\boldsymbol{\psi})}\right\}dM_i^T(t)\right]^{\otimes2},$$

and $M_i^T(t) = N_i^T(t) - \int_0^t Y_k^{**T}(u)\lambda_0(u)r(A_{k1}, A_{k2}, u; \boldsymbol{\psi})du$ is the martingale based on the counting process for outcome event. The sandwich variance estimator is obtained when both $\boldsymbol{\Sigma}_0$ and $\boldsymbol{\Sigma_1}$ are evaluated at the estimated weights and $\hat{\boldsymbol{\psi}}$.

Because the above robust variance estimator considers the weights $\Omega^{A_1,A_2}\Omega^C(G_i)$ as fixed known values (**?**), it could result in conservative (but still valid) inference. With time-invariant weights estimated by logistic regression in the cross-sectional treatment setting, the corrected robust sandwich variance estimator has been derived to achieve improved variance estimation for hazard ratio parameters (**?**). However, an extension to continuous-time weights is not trivial and currently unavailable. Alternatively, resampling method such as the bootstrap method (**??**) could be used to make more robust inference for $\boldsymbol{\psi}$ that accounts for the uncertainty of the weights.

2 Marginal Structural Cox Model Simulation Algorithm

Here we provide pseudocodes for the marginal structural cox model data simulation. We use the COVID-19 dataset as the foundation to set the values of the parameters in both the treatment assignment and marginal structural models. In our simulations, to reduce the impact of data sparsity, we set the maximum number of treatment initiations to be 4. To do this, the pseudocodes can be modified by setting treatment exposure status to one at all time points after the fourth treatment initiation throughout to the end of the follow-up period.

GET

$M \leftarrow 100$ (maximum follow-up); $\lambda_0 \leftarrow 0.005$; $n \leftarrow 1000$;

$\boldsymbol{\beta} \leftarrow (\beta_0, \beta_1, \beta_2, \beta_3) \leftarrow [\log(3/7), -0.5, -\log(1/2), \log(3/2)]$ (parameter vector for generating time-varying confounding variables L_2);

$\boldsymbol{\zeta} \leftarrow (\zeta_0, \zeta_1, \zeta_2, \zeta_3, \zeta_4) \leftarrow [\log(2/7), -\log(1/2), -0.5, \log(3/2), \log(2/3)]$ (parameter

vector for generating time-varying confounding variables L_1);

$\boldsymbol{\gamma} \leftarrow (\gamma_0, \gamma_1, \gamma_2, \gamma_3, \gamma_4, \gamma_5, \gamma_6, \gamma_7, \gamma_8, \gamma_9, \gamma_{10}, \gamma_{11})$

$\leftarrow [\log(2/7), 1/2, -1/2, -\log(3/5), 0.8, 0.5, 0.8, -0.5, 1/2, 1.2, -0.6, -0.3]$ (parameter vector for generating A_1);

$\boldsymbol{\eta} \leftarrow (\eta_0, \eta_1, \eta_2, \eta_3, \eta_4, \eta_5, \eta_6, \eta_7, \eta_8, \eta_9, \eta_{10}, \eta_{11})$

$\leftarrow [\log(3/7), 1/3, -1/3, -\log(2/5), 0.9, 0.6, 0.8, -0.5, 1/3, 0.9, -0.6, -0.4]$ (parameter vector for generating A_2);

$\psi_1 \leftarrow -0.5$ (true log-hazard value representing the effect of treatment A_1)

$\psi_2 \leftarrow -0.3$ (true log-hazard value representing the effect of treatment A_2)

COMPUTE

for ID $\leftarrow 1$ to n (for each individual) **do**

INIT: $L_1(-1) \leftarrow 0; L_2(-1) \leftarrow 0; A_1(-1) \leftarrow 0; A_2(-1) \leftarrow 0; Y(0) \leftarrow 0; H(m) \leftarrow 0;$

$n_{A_1} \leftarrow 0; n_{A_2} \leftarrow 0; \tau_{A_1} \leftarrow 0; \tau_{A_2} \leftarrow 0$

$T^{\bar{0}} \sim$ Exponential(λ_0)

for $m \leftarrow 0$ to M **do**

$L_1(m) \leftarrow E\left(L_1(m) = l_1(m) \,|\, L_1(m-1), A_1(m-1), A_2(m-1), Y(m) = 0; \boldsymbol{\zeta}\right)$

$\leftarrow \zeta_0 + \zeta_1(1/\log T^{\bar{0}}) + \zeta_2 A_1(m-1) + \zeta_3 L_1(m-1) + \zeta_4 A_2(m-1)$

logit$(p_{L_2}) \leftarrow$ logit$P\left(L_2(m) = 1 \,|\, L_2(m-1), A_1(m-1), A_2(m-1), Y(m) = 0; \boldsymbol{\beta}\right)$

$\leftarrow \beta_0 + \beta_1 A_1(m-1) + \beta_2 L_2(m-1) + \beta_3 A_2(m-1)$

$L_2(m) \sim$ Bernoulli(p_{L_2})

logit$(p_{A_1}) =$ logit$P(A_1(m) = 1 \,|\, L_1(m), L_1(m-1), L_2(m), L_2(m-1), A_1(m-1),$

$A_2(m-1), n_{A_1}, Y(m) = 0; \boldsymbol{\gamma})$

$= \gamma_0 + \gamma_1 A_1(m-1) + \gamma_2 (L_2(m-1))^2 + \gamma_3 (L_1(m-1))^2 + \gamma_4 (A_1(m-1) L_1(m))$

$+\gamma_6 (L_1(m) L_2(m)) + \gamma_7 (A_1(m-1) L_2(m)) + \gamma_8 A_2(m-1)$

$+\gamma_9(A_2(m-1)L_1(m)) + \gamma_{10}(A_2(m-1)L_2(m)) + \gamma_{11} n_{A_1}$

if $A_1(m-1) = 0$ or $m-1 = \tau_{A_1}$ **then**

$A_1(m) \sim$ Bernoulli(p_{A_1})

if $A_1(m) = 1$ **then**

$\delta_{A_1} \sim$ zero-truncated Poisson(10) (treatment duration after initiation)

$\tau_{A_1} \leftarrow m + \delta_{A_1}$

$A_1(m+1) : A_1(\max(\tau_{A_1}, M)) \leftarrow 1$

$n_{A_1} \leftarrow n_{A_1} + 1$

end if

end if

$$\begin{aligned}\text{logit}(p_{A_2}) = \text{logit}P(&A_2(m) = 1 \mid L_1(m), L_1(m-1), L_2(m), L_2(m-1), A_1(m),\\ &A_2(m-1), n_{A_2}, Y(m) = 0; \boldsymbol{\eta})\\ = \eta_0 &+ \eta_1 A_1(m-1) + \eta_2(L_2(m-1))^2 + \eta_3(L_1(m-1))^2 + \eta_4(A_1(m)L_1(m))\\ &+\eta_6(L_1(m)L_2(m)) + \eta_7(A_1(m)L_2(m)) + \eta_8 A_2(m-1)\\ &+\eta_9(A_2(m-1)L_1(m)) + \eta_{10}(A_2(m-1)L_2(m)) + \eta_{11} n_{A_2}\end{aligned}$$

if $A_2(m-1) = 0$ or $m-1 = \tau_{A_2}$ **then**

$A_2(m) \sim$ Bernoulli(p_{A_2})

if $A_2(m) = 1$ **then**

$\delta_{A_2} \sim$ zero-truncated Poisson(9) (treatment duration after initiation)

$\tau_{A_2} \leftarrow m + \delta_{A_2}$

$A_2(m+1) : A_2(\max(\tau_{A_2}, M)) \leftarrow 1$

$n_{A_2} \leftarrow n_{A_2} + 1$

end if

end if

$H_m \leftarrow H_m + \exp\{\psi_1 A_1(m) + \psi_2 A_2(m)\}$

if $T^{\bar{0}} \geq H_m$

$Y_{m+1} \leftarrow 0$

else

$Y_{m+1} \leftarrow 1$

$T \leftarrow m + (T^{\bar{0}} - H_m) \times \exp\{-\psi_1 A_1(m) - \psi_2 A_2(m)\}$

end if

end for m

end for ID

---

3 Additional details of recurrent events formulation

To formalize the treatment initiation process, we first consider a univariate treatment process N^{A_w}. We assume that the jumps of $A_w(t)$, i.e., $dA_w(t)$, is observed on certain subintervals of $[0, t^o]$ only. Specifically for individual i, we observe the stochastic process $A_{w,i}(t)$ on a set of intervals

$$\mathcal{E}_{w,i} = \bigcup_{j=1}^{J_i} (V_{w,ij}, U_{w,ij}],$$

where $0 \leq V_{w,i1} \leq U_{w,i1} \leq \ldots \leq V_{w,iJ_i} \leq U_{w,iJ_i} \leq t_{w,iK_i}$. This representation implies the following results. First, an individual can have at most $J_i \geq 1$ initiations of treatment w: if $U_{w,iJ_i} = t_{w,iK_i}$, then individual i has $J_i - 1$ treatment initiations; and if $U_{w,iJ_i} < t_{w,iK_i}$, then individual i has J_i treatment initiations. A special case where $J_i = 1$ and $U_{w,iJ_i} = t_{w,iK_i}$ corresponds to the situation where individual i is continuously eligible for treatment initiation and has not been treated with w during the follow-up. Second, once treatment is initiated, $A_{w,i}(t)$ is no longer stochastic until person i discontinues the treatment. This also suggests that the jth treatment initiation is observed at $U_{w,ij}$. This implication pertains to the "off" treatment period (referred to as the lack-of-color period in Figure 1), during which a patient becomes

eligible to receive the treatment. Third, we have

$$A_{w,i}(t) = 1, \quad \forall\, t \quad \in (U_{w,ij}, V_{w,i(j+1)}], j = 1, \ldots, J_i - 1.$$

In words, treatment status is equal to one deterministically on the discontinuous intervals of ineligibility (i.e., *on* treatment period). This implication involves the "on" treatment period (represented by the colored segment in Figure 1). Once the treatment commences at a specific time point, the patient continues with the treatment for a predetermined duration, throughout which the treatment status remains deterministically at one.

4 A justification for consistency using the Radon-Nikodym derivative

This section offers a heuristic rationale for the treatment weights employed in fitting our structural proportional hazards model. We employ arguments analogous to those presented by **?** and **?**, who utilized a Radon-Nikodym (R-N) derivative developed by **?** to construct and estimate weights that lead to consistent estimates of structural parameters in the context of censored survival outcomes and continuous outcomes, respectively. For brevity, we demonstrate the use of R-N assuming no censoring. For the scenario involving right censoring, the reasoning follows a similar approach.

Under randomization of treatment, the unbiased partial likelihood score equations can be written as $\sum_i^n D_i(\boldsymbol{\psi}) = 0$ (**??**), where

$$D_i(\boldsymbol{\psi}) = \int_0^\infty \left\{Z(A_{1i}, A_{2i}, t) - \bar{Z}(t; \boldsymbol{\psi})\right\} dN_i^T(t) = 0,$$

$Z(A_{1i}, A_{2i}, t)_{(3\times 1)} = [A_{1i}(t), A_{2i}(t), A_{1i}(t)A_{2i}(t)]^\top$, and

$$\bar{Z} = \frac{\sum_{k\in\mathcal{R}_t^T} Z(A_{k1}, A_{k2}, t) Y_k(t) r(A_{k1}, A_{k2}, t; \boldsymbol{\psi})}{\sum_{k\in\mathcal{R}_t^T} Y_k(t) r(A_{k1}, A_{k2}, t; \boldsymbol{\psi})}.$$

Now we consider the scenario in which treatment is nonrandomly allocated. Let $\mathbb{P}_R(\cdot)$ denote the data distribution under randomized treatment, and let $\mathbb{P}_O(\cdot)$ denote the same under non-random allocation of treatment. The observable data, with respect to treatment and outcome, for each individual, under either randomized or non-randomized allocation of treatment, is $\{\bar{A}_1(T^*), \bar{A}_2(T^*), T^*, \Delta^T\}$. Following **?**, under the conditional exchangeability assumption (A2) and some regularity conditions, including the Positivity assumption (A3), the distribution of $\{\bar{A}_1(T^*), \bar{A}_2(T^*), T^*, \Delta^T\}$ under $\mathbb{P}_R(\cdot)$ is absolutely continuous with respect to the distribution of $\{\bar{A}_1(T^*), \bar{A}_2(T^*), T^*, \Delta^T\}$ under $\mathbb{P}_O(\cdot)$, and a version of the R-N derivative is

$$E_o\left\{\frac{f^{A_1,A_2}\left(T^*\right)}{f^{A_1,A_2}\left(T^* \mid \bar{L}\left(T^*\right)\right)} \mid T^*, \Delta^T, \bar{L}(T^*)\right\}, \tag{3}$$

where $f^{A_1,A_2}(\cdot)$ is the joint probability density according to which treatments A_1 and A_2 are randomly allocated, and $f^{A_1,A_2}(\cdot \mid \cdot)$ is the conditional joint probability density.

An estimating equation that is a function of observed data and is unbiased under the distribution of $\mathbb{P}_R(\cdot)$ can be re-weighted by the R–N derivative to obtain an unbiased estimating equation using the same observed data, but now under the distribution $\mathbb{P}_O(\cdot)$ (**?**). We apply the R-N derivative in (3) to construct an unbiased estimating equation using the observed data under the distribution $\mathbb{P}_O(\cdot)$, by noticing that the partial likelihood estimating equation $\sum_i^n D_i(\boldsymbol{\psi})$ can be represented as three averages. We first rewrite $D_i(\boldsymbol{\psi})$ as

$$D_i(\boldsymbol{\psi}) = \int_0^\infty \left\{Z(A_{1i}, A_{2i}, t) - \frac{n^{-1}\sum_{k=1}^n Z(A_{k1}, A_{k2}, t)Y_k(t)r(A_{k1}, A_{k2}, t; \boldsymbol{\psi})}{n^{-1}\sum_{k=1}^n Y_k(t)r(A_{k1}, A_{k2}, t; \boldsymbol{\psi})}\right\} dN_i^T(t) = 0.$$

Under randomized assignment,

$$\begin{aligned} n^{-1}\sum_{k=1}^n Z(A_{k1}, A_{k2}, t)Y_k(t)r(A_{k1}, A_{k2}, t; \boldsymbol{\psi}) \;&\xrightarrow{P}\; E_R\left\{Z(A_{k1}, A_{k2}, t)Y_k(t)r(A_{k1}, A_{k2}, t)\right\} \\ &= M_1(t; \boldsymbol{\psi}), \end{aligned} \tag{4}$$

$$
\begin{aligned}
n^{-1}\sum_{k=1}^{n} Y_k(t) r(A_{k1}, A_{k2}, t; \boldsymbol{\psi}) &\stackrel{P}{\to} E_R\left\{Y_k(t) r(A_{k1}, A_{k2}, t)\right\} \\
&= M_0(t; \boldsymbol{\psi}),
\end{aligned} \tag{5}
$$

and

$$
\begin{aligned}
& n^{-1}\sum_{i=1}^{n} \int_0^{\infty} \left\{ Z(A_{1i}, A_{2i}, t) - \frac{M_1(t; \boldsymbol{\psi})}{M_1(t; \boldsymbol{\psi})} \right\} dN_i^T(t) \\
&\stackrel{P}{\to} E\left\{ \int_0^{\infty} \left\{ Z(A_{1i}, A_{2i}, t) - \frac{M_1(t; \boldsymbol{\psi})}{M_1(t; \boldsymbol{\psi})} \right\} dN_i^T(t) \right\}.
\end{aligned} \tag{6}
$$

Each of the three limits in (4), (5) and (6) is an expected value, taken over the distribution of treatment, applied to observable data under randomization. We can apply the R-N derivative in (3) to (4), (5) and (6), and obtain the weights for both the risk set and the score contribution. The weighted weighted partial score equation is

$$
\sum_{i=1}^{n} \int_0^{\infty} \Omega^{A_1,A_2}(t_{K_i}) \left\{ Z(A_{1i}, A_{2i}, t) - \bar{Z}^*(t; \boldsymbol{\psi}) \right\} dN_i^T(t) = 0,
$$

where the general form of the weight is $\Omega^{A_1,A_2}(t_{K_i}) = \dfrac{f^{A_1,A_2}\left(T^*\right)}{f^{A_1,A_2}\left(T^* \mid \bar{L}\left(T^*\right)\right)}$.

The continuous-time is a generalization of the discrete-time weight. For example, in the discrete-time setting with (two) nonrecurrent treatments, the stabilized inverse probability weights (we suppress subscript i for brevity) are given in the prior literature (**??**):

$$
\begin{aligned}
\Omega^{A_1,A_2}(t) = & \left\{ \prod_{\{k:t_k \le t\}} \frac{P\left(A_1(t_k) = a_1(t_k) \mid \bar{A}_1(t_{k-1}), \bar{A}_2(t_{k-1})\right)}{P\left(A_1(t_k) = a_1(t_k) \mid \bar{A}_1(t_{k-1}), \bar{A}_2(t_{k-1}), \bar{L}(t_{k-1}), T \ge t, C \ge t\right)} \right\} \times \\
& \left\{ \prod_{\{k:t_k \le t\}} \frac{P\left(A_2(t_k) = a_2(t_k) \mid \bar{A}_1(t_k), \bar{A}_2(t_{k-1})\right)}{P\left(A_2(t_k) = a_2(t_k) \mid \bar{A}_1(t_k), \bar{A}_2(t_{k-1}), \bar{L}(t_{k-1}), T \ge t, C \ge t\right)} \right\},
\end{aligned} \tag{7}
$$

where t_k's are a set of ordered discrete time points common to all individuals satisfying $0 = t_0 < t_1 < t_2 < \ldots \leq t$. In Section 3.2, we generalize these weights to the continuous-time setting, and further take into account the recurrent nature of the treatments.

5 Precise formulation of individual weights

When the number of time intervals in $[0, t]$ increases and ds approaches zero, the finite product over the number of time intervals of the individual partial likelihood will approach a product integral (**?**). Therefore, as we discuss in the main manuscript, each product term in the numerator and denominator of (7) can be generalized to the continuous-time setting as

$$
\begin{aligned}
&\prod_0^t \left\{D^{A_w}(s)\lambda^{A_w}(s \mid \bullet)ds\right\}^{dA_w(s)} \left\{D^{A_w}(s)\left(1 - \lambda^{A_w}(s \mid \bullet)ds\right)\right\}^{1-dA_w(s)} \\
= \; &\left[\prod_0^t \left\{D^{A_w}(s)\lambda^{A_w}(s \mid \bullet)\right\}^{\Delta A_w(s)}\right] \exp\left\{-\int_0^t D^{A_w}(s)\lambda^{A_w}(s \mid \bullet)ds\right\}, \qquad (8)
\end{aligned}
$$

where $\Delta A_w(t) = A_w(t) - A_w(t^-)$. For individual i, both factors in (8) need to be evaluated with respect to the individual's filtered counting process $\{N_{iq}^{A_w}(t) : 0 \leq t \leq t_{K_i}, q = 1, \ldots, Q_{w,i}\}$, where $Q_{w,i}$ is the number of initiations of treatment w for individual i.

As described in Supplementary Section 3, the number of treatment initiations for individual i, $Q_{w,i}$ can take three values: (i) $Q_{w,i} = 0$, (ii) $Q_{w,i} = J_i - 1$ or (iii) $Q_{w,i} = J_i$. Corresponding to the three cases, the quantity in (8) can be rewritten in explicit forms as

$$
\text{Quantity (8)} = \begin{cases} S^{A_w}\left(t_{K_i} \mid \bullet\right) & \text{if } Q_{w,i} = 0 \\ f^{A_w}\left(U_{i,J_i-1} \mid \bullet\right)\left\{S^{A_w}\left(V_{iJ_i} \mid \bullet\right) - S^{A_w}\left(t_{iK_i} \mid \bullet\right)\right\} & \text{if } Q_{w,i} = J_i - 1 \\ f^{A_w}\left(U_{iJ_i} \mid \bullet\right) & \text{if } Q_{w,i} = J_i, \end{cases}
$$

where S^{A_w} and f^{A_w} are the survival and density function of the filtered counting process for treatment A_w. In alignment with conventions established in prior literature (**??**), the exposure

weight for joint treatments A_1 and A_2 assumes a treatment order. By positing that treatment A_1 is administered infinitesimally earlier than treatment A_2, the intensity of initiating treatment A_2 at time t can be dependent on the status of treatment A_1 at time t, $A_1(t)$, and the status of treatment A_2 at time t^-, $A_2(t^-)$. Furthermore, the intensity of initiating treatment A_1 at time t can rely on $A_1(t^-)$ and $A_2(t^-)$. This assumption of ordered treatment administration is plausible in clinical contexts.

For exposition brevity, we define

$$\begin{aligned}
\bar{\mathcal{O}}_1(t) &= \{\bar{A}_1(t^-), \bar{A}_2(t^-), \bar{L}(t^-), T \geq t, C \geq t\} \\
\bar{\mathcal{O}}_2(t) &= \{\bar{A}_1(t), \bar{A}_2(t^-), \bar{L}(t^-), T \geq t, C \geq t\} \\
\bar{\mathcal{O}}^{A_1}(t) &= \{\bar{A}_1(t^-), \bar{A}_2(t^-), T \geq t, C \geq t\} \\
\bar{\mathcal{O}}^{A_2}(t) &= \{\bar{A}_1(t), \bar{A}_2(t^-), T \geq t, C \geq t\}
\end{aligned}$$

Putting this all together, the individual continuous-time stabilized inverse probability weight that corrects for time-varying confounding is given by $\Omega^{A_1,A_2}(t) = \Omega^{A_1}(t)\Omega^{A_2}(t)$ with Ω^{A_w} being:

$$\begin{aligned}
&\Omega^{A_w}(t_{K_i}) \\
&= \begin{cases}
\dfrac{S^{A_w}\left(t_{K_i} \mid \bar{\mathcal{O}}^{A_w}(t_{K_i})\right)}{S^{A_w}\left(t_{K_i} \mid \bar{\mathcal{O}}_w(t_{K_i})\right)} & \text{if } Q_{w,i} = 0 \\
\dfrac{f^{A_w}\left(U_{i,J_i-1} \mid \bar{\mathcal{O}}^{A_w}(U_{i,J_i-1})\right)\left\{S^{A_w}\left(V_{iJ_i} \mid \bar{\mathcal{O}}^{A_w}(V_{iJ_i})\right) - S^{A_w}\left(t_{iK_i} \mid \bar{\mathcal{O}}^{A_w}(t_{K_i})\right)\right\}}{f^{A_w}\left(U_{i,J_i-1} \mid \bar{\mathcal{O}}_w(U_{i,J_i-1})\right)\left\{S^{A_w}\left(V_{iJ_i} \mid \bar{\mathcal{O}}_w(V_{iJ_i})\right) - S^{A_w}\left(t_{iK_i} \mid \bar{\mathcal{O}}_w(t_{K_i})\right)\right\}} & \text{if } Q_{w,i} = J_i - 1 \\
\dfrac{f^{A_w}\left(U_{iJ_i} \mid \bar{\mathcal{O}}^{A_w}(U_{iJ_i})\right)}{f^{A_w}\left(U_{iJ_i} \mid \bar{\mathcal{O}}_w(U_{iJ_i})\right)} & \text{if } Q_{w,i} = J_i
\end{cases}
\end{aligned} \tag{9}$$

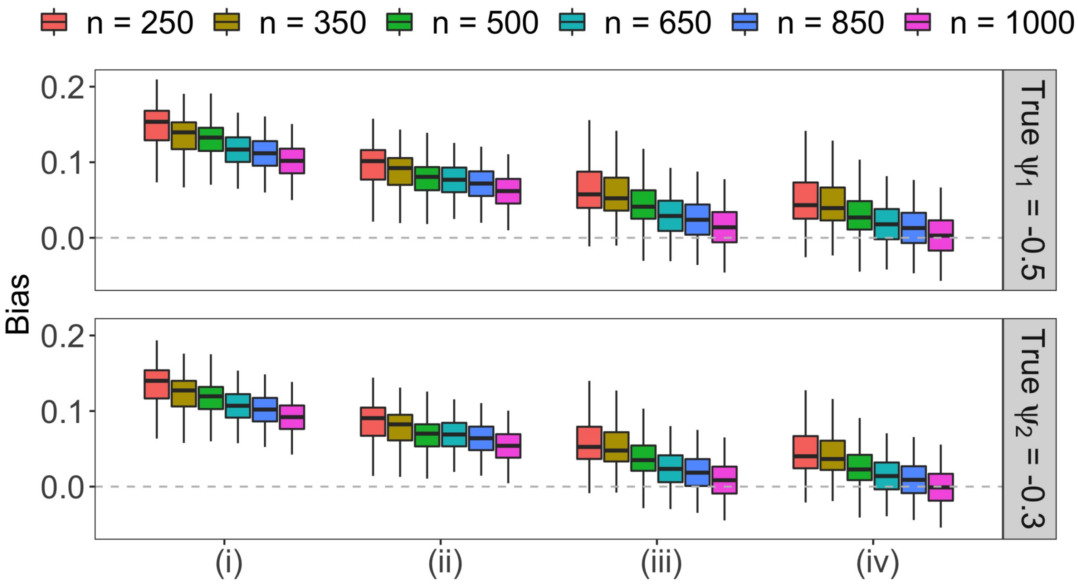

Figure 1: Biases in estimates of ψ_1 and ψ_2 for sample sizes $n=250$, 350, 500, 650, 850, and 1000 across 250 data replications, using four weight estimation approaches detailed in Section 3.4. Approach (i) uses main-effects Cox regression model and Nelson-Aalen estimator for baseline intensity. Approach (ii) uses kernel function smoothing of the Nelson-Aalen estimator in approach (i). Approach (iii) uses a survival forests model that accommodates time-varying covariates and Nelson-Aalen estimator for baseline intensity. Approach (iv) uses kernel function smoothing of the Nelson-Aalen estimator in approach (iii).

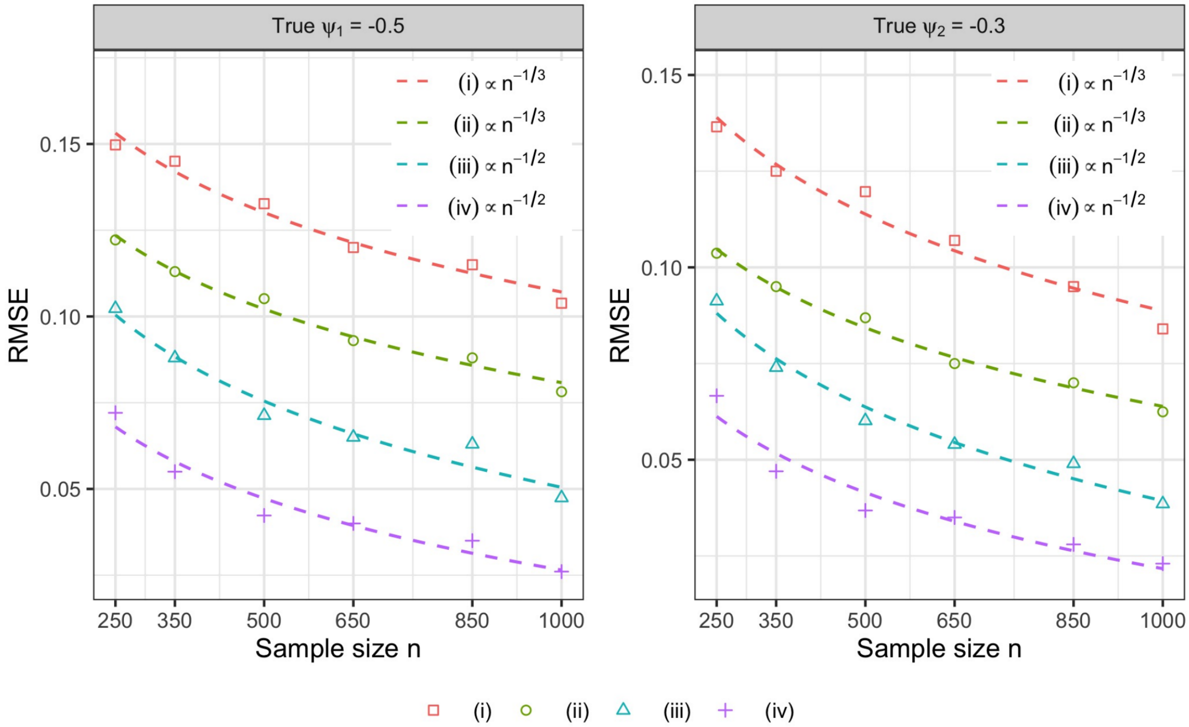

Figure 2: Approximate convergence rates of root-mean-square errors for estimating structural parameters ψ_1 and ψ_2 utilizing the four weighting estimators detailed in Section 3.4.

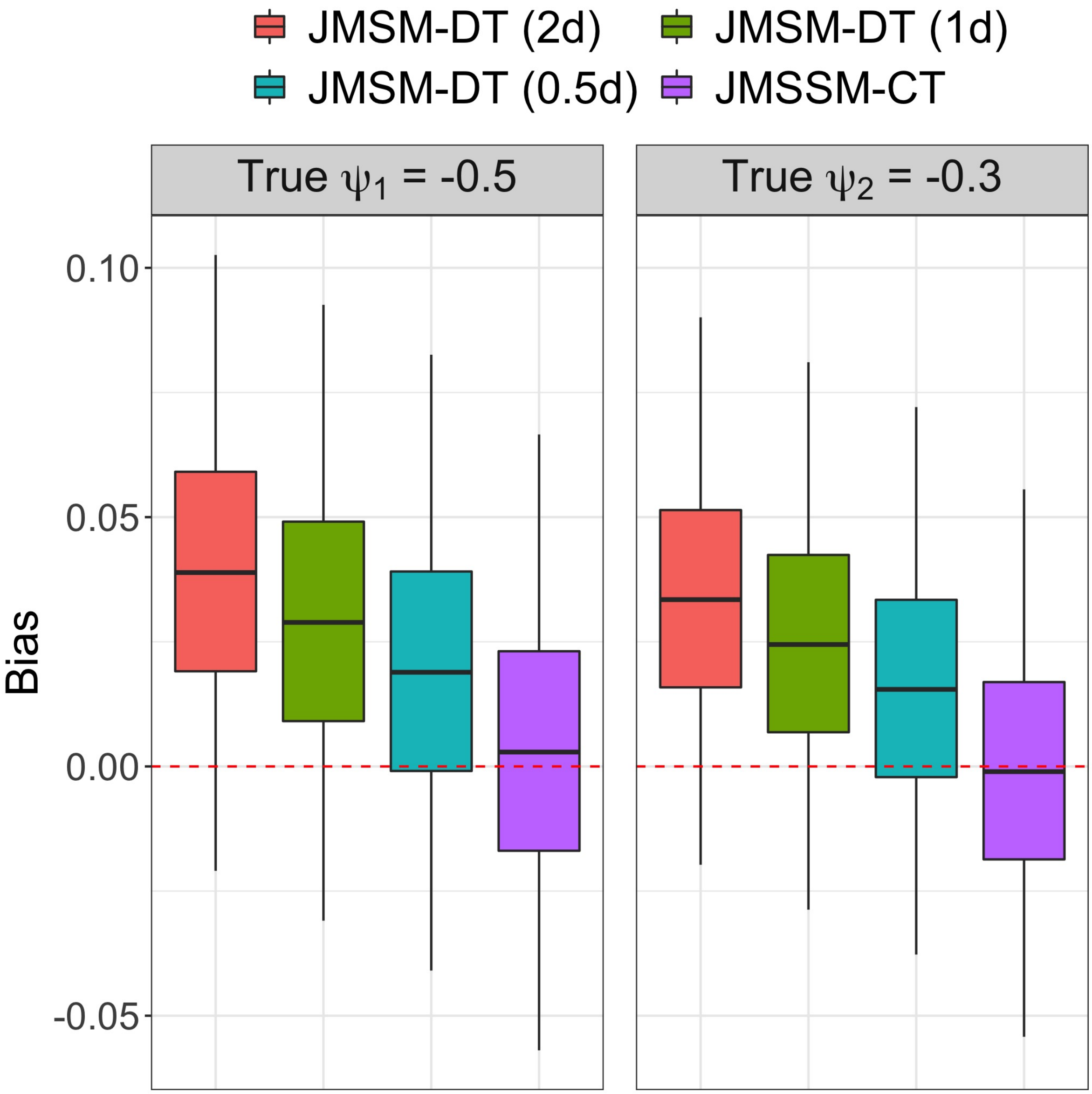

Figure 3: The distributions of biases across 250 simulation replications of the ragged longitudinal data with unaligned time points, in estimating the parameters ψ_1 and ψ_2 using the proposed JMSSM-CT method and the comparison method JMSM-DT. When implementing JMSM-DT, the follow-up time was respectively discretized into time intervals of length 0.5, 1 and 2 days, as the method requires aligned measurement time points.

6 Supplementary figures and tables

6.1 Additional simulation results

Table 1: Mean absolute bias (MAB), root mean square error (RMSE) and coverage probability (CP) for the estimates of ψ across 250 data replications with unaligned follow-up time points, using four weighting estimators (i)-(iv) described in Section 3.4.

weighting estimators	ψ_1			ψ_2		
	MAB	RMSE	CP	MAB	RMSE	CP
(i)	.102	.104	.048	.092	.094	.060
(ii)	.063	.067	.104	.055	.059	.112
(iii)	.023	.029	.936	.020	.025	.940
(iv)	.016	.022	.952	.015	.019	.952

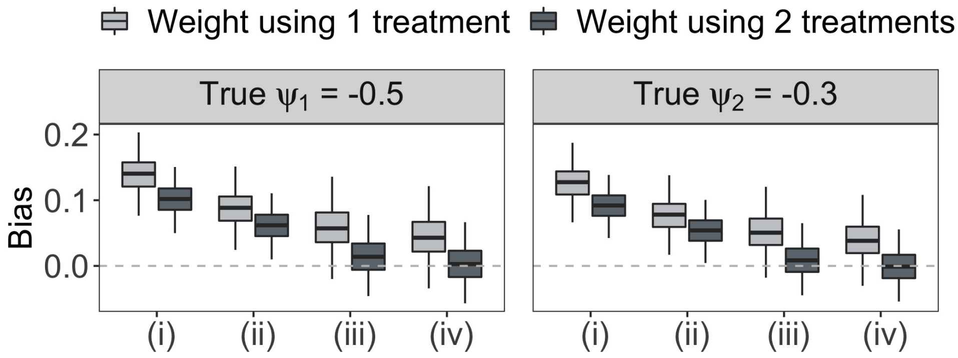

Figure 4: Biases in the estimate of ψ using two types of weighting estimators: one focusing on estimating a single treatment effect and one utilizing joint treatment weights.

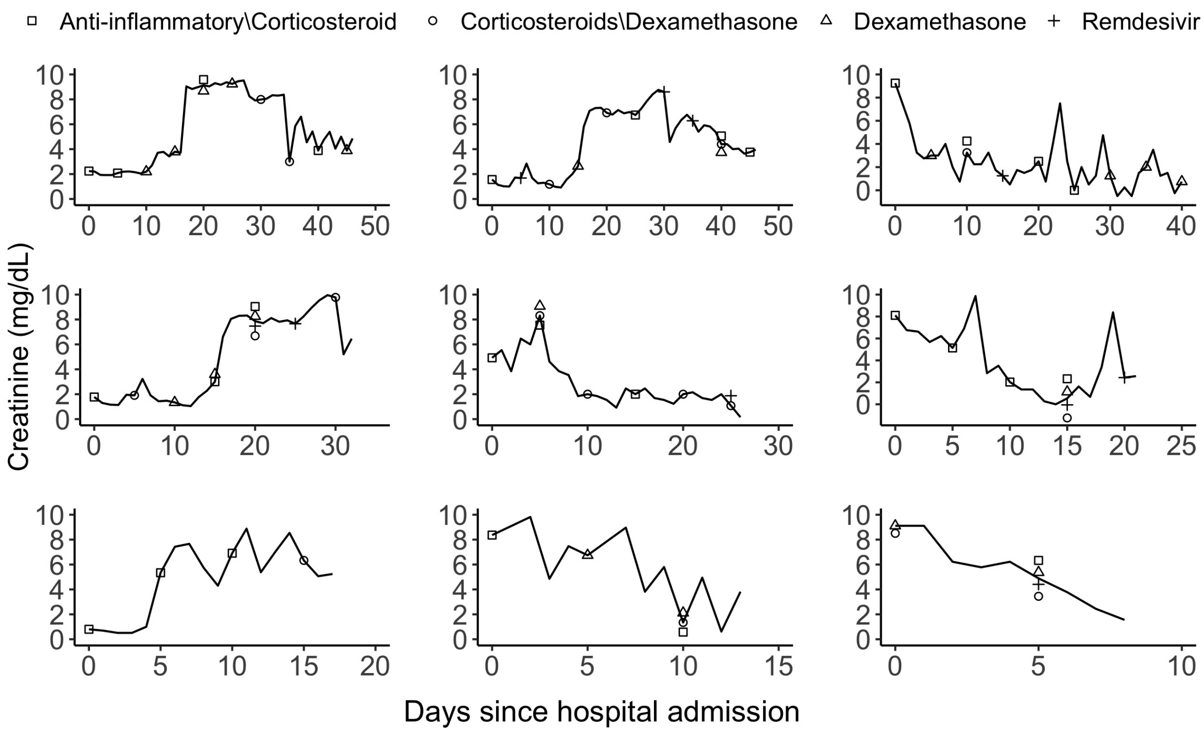

Figure 5: Trajectories of serum creatinine levels over the course of hospital stay for 9 randomly chosen patients. Symbols represent the types of treatment classes received by a patient at a given time.

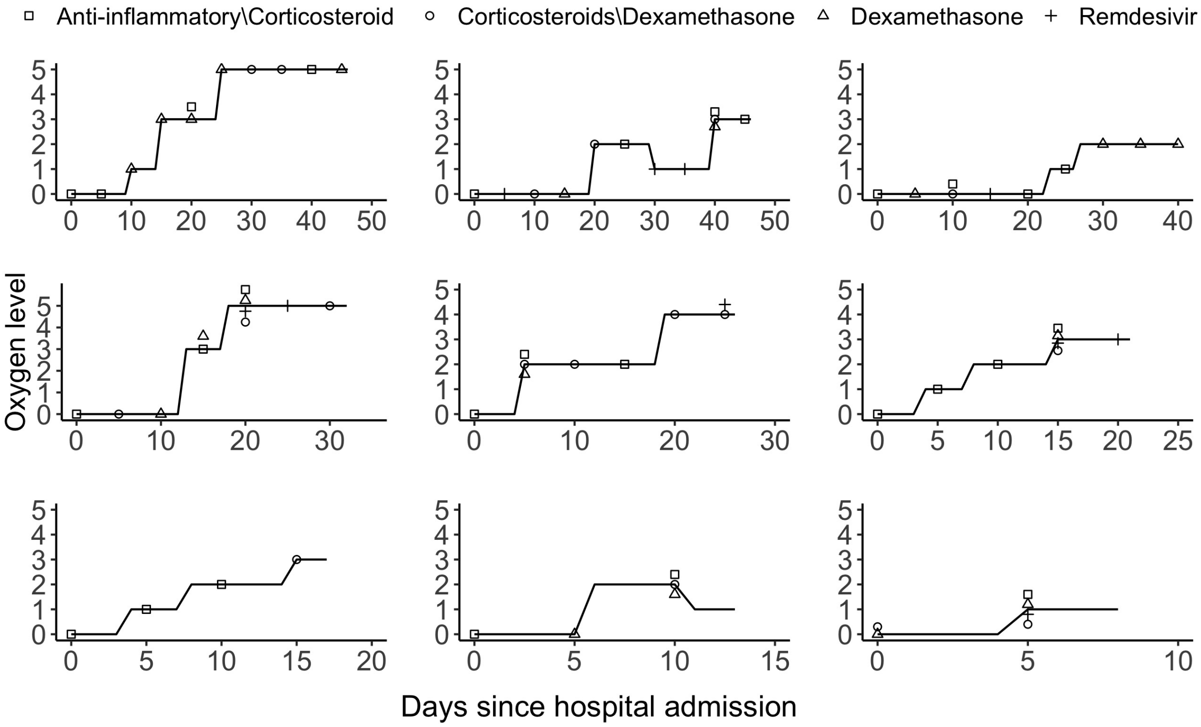

Figure 6: Trajectories of patient oxygen levels over the course of hospital stay for 9 randomly chosen patients. Symbols represent the types of treatment classes received by a patient at a given time.

Table 2: The distribution of the estimated individual time-varying weights from one random replication of the “ragged” longitudinal data with unaligned time points, for the proposed JMSSM-CT versus JMSM-DT. To estimate the weights, the random forests was used for JMSM-DT and four approaches (i)-(iv) (Section 3.4) were used for JMSSM-CT.

Methods	weighting estimators	Distribution of estimated weights				
		Minimum	First quartile	Mean	Third quartile	Maximum
JMSSM-CT	(i)	0.23	0.89	1.05	1.23	5.34
	(ii)	0.40	0.98	1.01	1.07	4.28
	(iii)	0.52	0.88	1.00	1.09	2.99
	(iv)	0.68	0.95	1.00	1.05	2.36
JMSM-DT	Random forests	0.43	0.90	1.03	1.11	3.65

6.2 Treatment classes for COVID-19

In Table 3, we provide detailed definitions of the four treatment classes for COVID-19 whose comparative effectiveness on in-hospital death was investigated in Section 6.

Table 3: Definitions of four treatment classes for COVID-19. iv:intravenous; po: by mouth.

Treatment classes	Medication (route)
Dexamethasone	Dexamethasone (iv), Dexamethasone (po)
Remdesivir	Remdesivir (iv)
Corticosteroids other than dexamethasone	Hydrocortisone (po), Hydrocortisone (iv), Methylprednisolone (po), Methylprednisolone (iv), Prednisolone (iv), Prednisone (po), Prednisone (iv)
Anti-inflammatory medications other than corticosteroids	Alpha-1-Proteinase Inhibitor (iv), Anakinra (iv), Azathioprine (po), Belatacept (iv), Eculizumab (iv), Envarsus (iv), Sarilumab (iv), Gengraf (po), Gengraf (iv), Hydrocortisone (po), Hydrocortisone (iv), Ibrutinib (po), Immune Globulin (iv), Infliximab (iv), Methylprednisolone (po), Methylprednisolone (iv), Montelukast (po), Prednisolone (iv), Prednisone (po), Prednisone (iv), Ruxolitinib (po), Tocilizumab (iv), Apremilast (po), Celecoxib (po), Dasatinib (po), Everolimus (iv), Gemtuzumab (iv), Ibuprofen (iv), Ibuprofen (po), Ifosfamide (iv), Leflunomide (po), Mesalamine (iv), Methotrexate (iv), Methylphenidate (iv), Mycophenolate (iv), Naproxen (po), Rituximab (iv), Sulfasalazine (po), Tacrolimus (iv), Pacritinib (po), Risankizumab (iv), Daratumumab (iv), Talquetamab (iv)

6.3 Patient oxygen levels

We describe how patient oxygen levels are categorized based on the use of ventilator in Table 4.

Table 4: Definitions of patient oxygen levels based on the use of ventilator

Patient oxygen level	Ventilator status
0	Room air
1	Cannula
2	Mask, Blow-by, Face tent, Oxyhood, Non-rebreather, RAM cannula
3	Continuous positive airway pressure machine, High flow nasal cannula, Hudson prongs
4	Bilevel positive airway pressure machine, Tracheostomy mask
5	Tracheotomy, Transtracheal oxygen therapy, Ventilator, Endotracheal tube, T-shaped tubing connected to an endotracheal tube, Nasal synchronized intermittent mandatory ventilation

6.4 Additional COVID data analysis results

The inclusion criteria for the COVID-19 dataset are as follows: individuals aged 18 years or older, confirmed COVID-19 diagnosis via a positive reverse-transcriptase PCR test conducted on a nasopharyngeal swab, and hospital admission at one of the five hospitals within the Mount Sinai Health System between February 25, 2020, and February 26, 2021. No exclusion criteria were applied.

Table 5: Baseline characteristics of patients from the COVID-19 data. Summary statistics are represented as mean (standard deviation [SD]) for continuous variables and No. (%) for discrete variables.

Characteristics	$N = 11286$
Age (years), mean (SD)	64.59 (18.16)
Gender N (%)	
Male	6137 (54.4)
Female	5149 (45.6)
Race, N (%)	
White	3241 (28.7)
Black	2829 (25.1)
Asian	647 (5.7)
Others	4569 (40.5)
Ethnicity N (%)	
Hispanic	2972 (26.3)
non-Hispanic	8314 (73.7)
Asthma N (%)	
Yes	1082 (9.6)
No	10204 (90.4)
COPD N (%)	
Yes	734 (6.5)
No	10552 (93.5)
Hypertension N (%)	
Yes	5593 (49.6)
No	5693 (50.4)
Cancer N (%)	
Yes	1161 (10.3)
No	10125 (89.7)
Coronary artery disease N (%)	
Yes	2227 (19.7)
No	9059 (80.3)
Diabetes N (%)	
Yes	2809 (24.9)
No	8477 (74.1)
Smoking status N (%)	
Current	598 (5.3)
Former	2597 (23.0)
Never	6350 (56.3)
Unknown	1741 (15.4)
Hospital site N (%)	
Mount Sinai Brooklyn	1671 (14.8)
Mount Sinai Petrie	771 (6.8)
Mount Sinai Queens	1565 (13.9)
Mount Sinai St. Luke's	1828 (16.2)
Mount Sinai West	1304 (11.6)
Mount Sinai Main Hospital	4147 (36.7)

Abbreviations: SD = standard deviation;

Table 6: Comparing the proposed JMSSM-CT with discrete-time based method JMSM-DT in estimating the joint and interactive effects $\hat{\psi}$ (log hazard ratio) of COVID-19 treatments and associated 95% confidence intervals (CI), using the COVID-19 dataset drawn from the Epic electronic medical records system if the Mount Sinai Medical Center. The composite outcome of in-hospital death or admission to ICU was used. Confidence intervals were estimated via the robust sandwich variance estimators. "×" denotes treatment interaction. The weighting estimator (iv) was used for JMSSM-CT. For JMSM-DT, the follow up time was discretized in the space of 1, 3, and 5 days. The smallest unit of time in the COVID-19 data is 1 day.

Treatment orders	$\hat{\psi}$ (95% Confidence Interval)			
	JMSSM-CT	JMSM-DT (1d)	JMSM-DT (3d)	JMSM-DT (5d)
Dexamethasone	$-.20(-.35, -.06)$	$-.22(-.39, -.05)$	$-.25(-.45, -.05)$	$-.27(-.55, .01)$
Remdesivir	$-.53(-.75, -.31)$	$-.50(-.76, -.25)$	$-.48(-.76, -.20)$	$-.42(-.74, -.10)$
Corticosteroids other than dexamethasone	$-.08(-.29, .19)$	$-.10(-.36, .21)$	$-.12(-.43, .22)$	$-.14(-.48, .24)$
Anti-inflammatory medications other than corticosteroids	$-.05(-.56, .47)$	$-.07(-.60, .48)$	$-.09(-.66, .49)$	$-.13(-.75, .51)$
Remdesivir × Corticosteroids other than dexamethasone	$-.74(-.95, -.52)$	$-.78(-1.01, -.55)$	$-.81(-1.07, -.56)$	$-.83(-1.14, -.52)$

Table 7: Causal inferences about the treatment effects of COVID-19 medications on the bases of 14-day counterfactual survival probability and 14-day restricted mean survival time (RMST), using proposed JMSSM-CT with weighting estimator (iv) with additive hazard outcome model. The composite outcome of in-hospital death or admission to ICU was used. The 95% Confidence intervals were estimated using nonparametric bootstrap with 100 repetitions.

Treatment	14-day counterfactual survival probability	14-day counterfactual RMST
Dexamethasone	0.860 (0.835, 0.882)	13.11 (12.84, 13.49)
Remdesivir	0.892 (0.870, 0.902)	13.12 (12.85, 13.48)
Corticosteroids other than dexamethasone	0.832 (0.812, 0.849)	12.63 (12.38, 12.97)
Anti-inflammatory medications other than corticosteroids	0.814 (0.783, 0.833)	12.74 (12.32, 13.07)
Remdesivir + Corticosteroids other than dexamethasone	0.912 (0.881, 0.939)	13.21 (12.98, 13.52)

Table 8: The joint and interactive effect estimates $\hat{\psi}$ (log hazard ratio) of COVID-19 treatments and associated 95% confidence intervals (CI), using the COVID-19 dataset drawn from the Epic electronic medical records system of the Mount Sinai Medical Center. The composite outcome of in-hospital death or admission to ICU was used. The weighting estimator (iv) (Section 3.3) was used. The joint treatment weights were estimated using four different choices of treatment order. Confidence intervals were estimated via the robust sandwich variance estimators. "$\times$" denotes treatment interaction. A_1 = dexamethasone. A_2 = corticosteroids other than dexamethasone. A_3 = remdesivir. A_4 = anti-inflammatory medications other than corticosteroids. $\rightarrow$ denotes treatment order. For example, $A_1 \rightarrow A_2 \rightarrow A_3 \rightarrow A_4$ indicates that the joint treatment weights Ω^{A_1,A_2,A_3,A_4} are estimated by $\Omega^{A_1,A_2,A_3,A_4} = \Omega^{A_1} \times \Omega^{A_2 \mid A_1} \times \Omega^{A_3 \mid A_1,A_2} \times \Omega^{A_4 \mid A_1,A_2,A_3}$.

Treatment orders	$\hat{\psi}$ (95% Confidence Interval)			
	$A_1 \rightarrow A_2 \rightarrow A_3 \rightarrow A_4$	$A_4 \rightarrow A_3 \rightarrow A_2 \rightarrow A_1$	$A_1 \rightarrow A_3 \rightarrow A_4 \rightarrow A_2$	$A_4 \rightarrow A_2 \rightarrow A_1 \rightarrow A_3$
Dexamethasone	$-.20(-.35, -.06)$	$-.22(-.43, -.01)$	$-.23(-.38, -.08)$	$-.21(-.41, -.02)$
Remdesivir	$-.53(-.75, -.31)$	$-.48(-.74, -.22)$	$-.56(-.79, -.33)$	$-.50(-.74, -.26)$
Corticosteroids other than dexamethasone	$-.08(-.29, .19)$	$-.02(-.33, .29)$	$-.12(-.37, .13)$	$-.04(-.34, .26)$
Anti-inflammatory medications other than corticosteroids	$-.05(-.56, .47)$	$-.01(-.59, .57)$	$-.08(-.63, .47)$	$-.03(-.58, .51)$
Remdesivir $\times$ Corticosteroids other than dexamethasone	$-.74(-.95, -.52)$	$-.65(-.92, -.38)$	$-.80(-1.05, -.55)$	$-.68(-.94, -.44)$
