# Supplementary material for: ESTIMATING THE CAUSAL EFFECTS OF MULTIPLE INTERMITTENT TREATMENTS WITH APPLICATION TO COVID-19
Source: ArXiv. 2021 Sep 27:arXiv:2109.13368v3. Preprint. [Version 3] (PMC8722604)
Supplement: Supplementary file 1 [file NIHPP2109.13368V3-supplement-1.pdf]

WEB-BASED SUPPLEMENTARY MATERIALS FOR "ESTIMATING THE CAUSAL EFFECTS OF MULTIPLE INTERMITTENT TREATMENTS WITH APPLICATION TO COVID-19" BY HU ET AL.

arXiv:2109.13368v3 [stat.ME] 6 May 2022

S1. Additional technical details.

S1.1. *The kernel function estimator.* In Section 3.4, four approaches are described to estimate the stabilized time-varying inverse probability of treatment weights. The Nelson-Aalen estimator of the baseline intensity is smoothed by means of kernel functions in approach (ii) and approach (iv). For exposition brevity, consider a simple multiplicative intensity model for treatment initiation

$$\lambda(t) = \lambda_0(t) r\left(\bar{L}(t), \beta\right) Y(t),$$

where $\lambda_0(t)$ is the baseline intensity rate function, $r\left(\bar{L}(t), \beta\right) > 0$ is the (time-dependent) intensity ratio function parameterized by β, and $Y(t)$ is the at risk indicator. The cumulative baseline hazard is $\Lambda_0(t) = \int_0^t \lambda_0(s)ds$. The kernel function estimator for $\lambda_0(t)$ is derived by smoothing the increments of the Nelson-Aalen estimator of $\hat{\Lambda}_0$ as

$$\hat{\lambda}_0(t) = b^{-1} \int_{\mathscr{T}} K\left(\frac{t-s}{b}\right) d\hat{\Lambda}_0(s),$$

where K is the kernel function, which is a bounded function on [-1,1] and has integral 1, and the bandwidth b is a positive parameter governing the amount of smoothness. Commonly used kernel functions include the standard normal density function $K(x) = \phi(x)$ and the Epanechnikov kernel function $K(x) = 0.75(1-x^2)$, $|x| \leq 1$. Andersen et al. (1993) shows that the kernel function estimator $\hat{\lambda}_0(t)$ is consistent and asymptotically normal provided that there exists a sequence of positive constants $\{a_n\}$, increasing to infinity as $n \to \infty$, and that the bandwidth tends to zero more slowly than a_n^{-2} as $n \to \infty$.

Placed in the framework of recurrent events for a treatment A_w that can have multiple "start/stop" switches as described in Section 3.1, the intensity of the qth treatment initiation $\lambda_q^{A_w}(t)$ is smoothed by means of kernel function estimator of the baseline intensity function $\lambda_{0q}^{A_w}(t)$.

In our simulations (Section 5) and COVID-19 case study (Section 6), we used both the standard normal density $\phi(x)$ and Epanechnikov kernel functions and they yielded similar

results. We presented results from the normal density kernel function. Following Andersen et al. (1993), we chose the optimal bandwidth b as the value that minimizes the mean integrated squred error (MISE)

$$\text{MISE}(\hat{\lambda}_0) = E\int_0^{t^o} \left[\hat{\lambda}_0(t) - \lambda(t)\right]^2 dt.$$

S1.2. *Random survival forests accommodating time-varying covariates.* Approach (iii) uses a recent random survival forests model (Yao et al., 2020) that accommodates time-varying covariates to reduce the parametric assumptions about the form of the intensity ratio function required by the usual proportional intensity regression model. Yao et al. (2020) proposed a forest method that estimates the survival function by three steps. In step (1), reformat the data in the counting process structure, that is, for individual i, the time-varying covariate $L^{(i)}(t)$ will be represented as $L^{(i)}(t) = l_j^{(i)}$, $t \in \left[t_j^{(i)}, t_{j+1}^{(i)}\right)$, $j = 0, \ldots, J^{(i)} - 1$. Then split the individual i observation into $J^{(i)}$ pseudo subject observations: $\left(t_j^{(i)}, t_{j+1}^{(i)}, \delta_j^{(i)}, l_j^{(i)}\right)$ with left-truncated right-censored (LTRC) times $t_j^{(i)}, t_{j+1}^{(i)}$ and event indicator $\delta_j^{(i)}$ for the time interval $\left[t_j^{(i)}, t_{j+1}^{(i)}\right)$. Pool the counting process styled records from N subjects to create a list of pseudo-subjects,

$$\left\{t_l', t_{l+1}', \delta_l', l_l'\right\}_{l=1}^{n}, \; n = \sum_{i}^{N} J^{(i)}. \tag{1}$$

The set of pseudo-subjects is treated as if they were independent. Step (2) is to apply the forest algorithms on the reformatted dataset given in (1), to fit a model. In step (3), given a particular stream of covariate values at the corresponding time values, a survival function estimate is constructed based on the outputs of the forest algorithms.

We now briefly describe the forest algorithms. Yao et al. (2020) extended the relative risk forests, which combines the relative risk trees (LeBlanc and Crowley, 1992) with random forest methodology (Breiman, 2001), for LTRC data by modifying the splitting criteria. The splitting criterion under the relative risk framework is to maximize the reduction in the one-step deviance between the log-likelihood of the saturated model and the maximized log-likelihood. Let $\mathcal{R}_h$ denote the set of observations that fall into the node h. Let Λ_0 index the baseline cumulative hazard function, φ_h represent the nonnegative relative risk of the node h, and t_l and δ_l be the time and event indicator of the lth observation $\forall l \in \mathcal{R}_h$. Given the right censored observations (t_l, δ_l), the full likelihood deviance residual for node h is defined as

$$d_h = \sum_{l\in\mathcal{R}_h} 2\left\{\delta_l \log\left(\frac{\delta_l}{\hat{\Lambda}_0(t_l)\hat{\varphi}_h}\right) - \left(\delta_l - \hat{\Lambda}_0(t_l)\hat{\varphi}_h\right)\right\}. \tag{2}$$

Two steps are needed to modify the splitting rule and obtain the deviance residual appropriate for LTRC data (Fu and Simonoff, 2017). First, compute the estimated cumulative hazard function $\hat{\Lambda}_0(\cdot)$ based on all pseudo-subject observations. Second, replace $\hat{\Lambda}_0(t_l)$ in (2) with $\hat{\Lambda}_0(t'_{l+1}) - \hat{\Lambda}_0(t'_l)$, and replace δ_l in (2) with δ'_l. We refer to Yao et al. (2020) for more detailed description of the random survival forests model.

S1.3. *Variance estimation.* Since our estimators for $\boldsymbol{\psi}$ (marginal structural proportional hazards model parameter) can be considered as solution to the weighted partial score equation, we consider the robust sandwich variance estimator as a convenience device to construct confidence intervals. The robust sandwich variance estimator has been considered, for example, in Xiao, Abrahamowicz and Moodie (2010), and Karim et al. (2017, 2018), and has been shown to be at most conservative under the discrete-time setting. We use this estimator for inference in conjunction with our continuous-time stabilized inverse probability weights, and formally evaluate its performance in our simulations. We briefly describe the robust sandwich variance estimator below. With the continuous-time weights, we focus on equation (11) in the main text with two treatments:

$$\sum_{i=1}^{n} \int_0^\infty \Omega^{A_1,A_2}\Omega^C(G_i) \left\{ Z(A_{1i}, A_{2i}, t) - \frac{S^{(1)}(t;\boldsymbol{\psi})}{S^{(0)}(t;\boldsymbol{\psi})} \right\} dN_i^T(t) = 0,$$

where $\Omega^{A_1,A_2}\Omega^C(G_i)$ is the weight for time-varying treatments A_1 and A_2 and censoring (in continuous time), $Z(A_{1i}, A_{2i}, t)_{(3\times 1)} = [A_{1i}(t), A_{2i}(t), A_{1i}(t)A_{2i}(t)]^\top$, and

$$S^{(0)}(t;\boldsymbol{\psi}) = \sum_{k\in\mathcal{R}_t^T} Y_k^{**T}(t) r(A_{k1}, A_{k2}, t; \boldsymbol{\psi})$$

$$S^{(1)}(t;\boldsymbol{\psi}) = \sum_{k\in\mathcal{R}_t^T} Z(A_{k1}, A_{k2}, t) Y_k^{**T}(t) r(A_{k1}, A_{k2}, t; \boldsymbol{\psi}).$$

In the above definition, $\mathcal{R}_t^T$ refers to the collection of subjects still at risk for the outcome event at time t, $Y_k^{**T}(t) = \Omega^C(G_i)\Omega^{A_1,A_2}(t_{K_i})Y_k^T(t)$, where $Y_i^T(t)$ is the at-risk function for the outcome event, and $r(a_1, a_2, t) = \exp\{\psi_1 a_1(t) + \psi_2(t)a_2(t) + \psi_3 a_1(t)a_2(t)\}$. Now further define

$$S^{(2)}(t;\boldsymbol{\psi}) = \sum_{k\in\mathcal{R}_t^T} Z(A_{k1}, A_{k2}, t)^{\otimes 2} Y_k^{**T}(t) r(A_{k1}, A_{k2}, t; \boldsymbol{\psi}),$$

with $a^{\otimes 2} = aa^\top$ for any vector a. Then the robust sandwich variance estimator takes the form $\boldsymbol{\Sigma}_0^{-1}\boldsymbol{\Sigma}_1\boldsymbol{\Sigma}_0^{-1}$ (Binder, 1992), where

$$\boldsymbol{\Sigma}_0 = \sum_{i=1}^{n} \int_0^\infty \Omega^{A_1,A_2}\Omega^C(G_i) \left\{ \frac{S^{(2)}(t;\boldsymbol{\psi})}{S^{(0)}(t;\boldsymbol{\psi})} - \frac{S^{(1)}(t;\boldsymbol{\psi})^{\otimes 2}}{S^{(0)}(t;\boldsymbol{\psi})} \right\} dN_i^T(t),$$

and

$$\boldsymbol{\Sigma}_1 = \sum_{i=1}^{n} \left[\int_0^\infty \Omega^{A_1,A_2}\Omega^C(G_i) \left\{ Z(A_{1i}, A_{2i}, t) - \frac{S^{(1)}(t;\boldsymbol{\psi})}{S^{(0)}(t;\boldsymbol{\psi})} \right\} dM_i^T(t) \right]^{\otimes 2},$$

and $M_i^T(t) = N_i^T(t) - \int_0^t Y_k^{**T}(u)\lambda_0(u)r(A_{k1}, A_{k2}, u; \boldsymbol{\psi})du$ is the martingale based on the counting process for outcome event. The sandwich variance estimator is obtained when both $\boldsymbol{\Sigma}_0$ and $\boldsymbol{\Sigma_1}$ are evaluated at the estimated weights and $\hat{\boldsymbol{\psi}}$.

Because the above robust variance estimator considers the weights $\Omega^{A_1,A_2}\Omega^C(G_i)$ as fixed known values (Binder, 1992), it could result in conservative (but still valid) inference. With time-invariant weights estimated by logistic regression in the cross-sectional treatment setting, the corrected robust sandwich variance estimator has been derived to achieve improved variance estimation for hazard ratio parameters (Shu et al., 2021). However, an extension to continuous-time weights is not trivial and currently unavailable. Alternatively, resampling method such as the bootstrap method (Brumback et al., 2004; Cole and Hernán, 2008) could be used to make more robust inference for $\boldsymbol{\psi}$ that accounts for the uncertainty of the weights. Although theoretical valid, this resampling approach is not pursued in our current work due to the substantially more intensive computations associated with repeated estimation of complex weights under the recurrent event framework.

S2. Marginal Structural Cox Model Simulation Algorithm. Here we provide pseudocodes for the marginal structural cox model data simulation. We use the COVID-19 dataset as the foundation to set the values of the parameters in both the treatment assignment and marginal structural models. In our simulations, to reduce the impact of data sparsity, we set the maximum number of treatment initiations to be 4. To do this, the pseudocodes can be modified by setting treatment exposure status to one at all time points after the fourth treatment initiation throughout to the end of the follow-up period.

GET

$M \leftarrow 100$ (maximum follow-up); $\lambda_0 \leftarrow 0.005$; $n \leftarrow 1000$;

$\boldsymbol{\beta} \leftarrow (\beta_0, \beta_1, \beta_2, \beta_3) \leftarrow [\log(3/7), -0.5, -\log(1/2), \log(3/2)]$ (parameter vector for generating time-varying confounding variables L_2);

$\boldsymbol{\zeta} \leftarrow (\zeta_0, \zeta_1, \zeta_2, \zeta_3, \zeta_4) \leftarrow [\log(2/7), -\log(1/2), -0.5, \log(3/2), \log(2/3)]$ (parameter vector for generating time-varying confounding variables L_1);

$\boldsymbol{\gamma} \leftarrow (\gamma_0, \gamma_1, \gamma_2, \gamma_3, \gamma_4, \gamma_5, \gamma_6, \gamma_7, \gamma_8, \gamma_9, \gamma_{10}, \gamma_{11})$

$\leftarrow [\log(2/7), 1/2, -1/2, -\log(3/5), 0.8, 0.5, 0.8, -0.5, 1/2, 1.2, -0.6, -0.3]$ (parameter vector for generating A_1);

$\boldsymbol{\eta} \leftarrow (\eta_0, \eta_1, \eta_2, \eta_3, \eta_4, \eta_5, \eta_6, \eta_7, \eta_8, \eta_9, \eta_{10}, \eta_{11})$

$\leftarrow [\log(3/7), 1/3, -1/3, -\log(2/5), 0.9, 0.6, 0.8, -0.5, 1/3, 0.9, -0.6, -0.4]$ (parameter vector for generating A_2);

$\psi_1 \leftarrow -0.5$ (true log-hazard value representing the effect of treatment A_1)

$\psi_2 \leftarrow -0.3$ (true log-hazard value representing the effect of treatment A_2)

COMPUTE

for ID $\leftarrow 1$ to n (for each individual) **do**

INIT: $L_1(-1) \leftarrow 0; L_2(-1) \leftarrow 0; A_1(-1) \leftarrow 0; A_2(-1) \leftarrow 0; Y(0) \leftarrow 0; H(m) \leftarrow 0;$

$n_{A_1} \leftarrow 0; n_{A_2} \leftarrow 0; \tau_{A_1} \leftarrow 0; \tau_{A_2} \leftarrow 0$

$T^{\bar{0}} \sim$ Exponential(λ_0)

for $m \leftarrow 0$ to M **do**

$L_1(m) \leftarrow E\,(L_1(m) = l_1(m) \,|\, L_1(m-1), A_1(m-1), A_2(m-1), Y(m) = 0; \boldsymbol{\zeta})$

$\leftarrow \zeta_0 + \zeta_1(1/\log T^{\bar{0}}) + \zeta_2 A_1(m-1) + \zeta_3 L_1(m-1) + \zeta_4 A_2(m-1)$

logit$(p_{L_2}) \leftarrow$ logit$P\,(L_2(m) = 1 \,|\, L_2(m-1), A_1(m-1), A_2(m-1), Y(m) = 0; \boldsymbol{\beta})$

$\leftarrow \beta_0 + \beta_1 A_1(m-1) + \beta_2 L_2(m-1) + \beta_3 A_2(m-1)$

$L_2(m) \sim$ Bernoulli(p_{L_2})

logit$(p_{A_1}) =$ logit$P(A_1(m) = 1 \,|\, L_1(m), L_1(m-1), L_2(m), L_2(m-1), A_1(m-1),$

$A_2(m-1), n_{A_1}, Y(m) = 0; \boldsymbol{\gamma})$

$= \gamma_0 + \gamma_1 A_1(m-1) + \gamma_2(L_2(m-1))^2 + \gamma_3(L_1(m-1))^2 + \gamma_4(A_1(m-$

$1)L_1(m))$

$+\gamma_6(L_1(m)L_2(m)) + \gamma_7(A_1(m-1)L_2(m)) + \gamma_8 A_2(m-1)$

$+\gamma_9(A_2(m-1)L_1(m)) + \gamma_{10}(A_2(m-1)L_2(m)) + \gamma_{11} n_{A_1}$

if $A_1(m-1) = 0$ or $m - 1 = \tau_{A_1}$ **then**

$A_1(m) \sim$ Bernoulli(p_{A_1})

if $A_1(m) = 1$ **then**

$\delta_{A_1} \sim$ zero-truncated Poisson(10) (treatment duration after initiation)

$\tau_{A_1} \leftarrow m + \delta_{A_1}$

$A_1(m+1) : A_1(\max(\tau_{A_1}, M)) \leftarrow 1$

$n_{A_1} \leftarrow n_{A_1} + 1$

end if

end if

logit$(p_{A_2}) =$ logit$P(A_2(m) = 1 \,|\, L_1(m), L_1(m-1), L_2(m), L_2(m-1), A_1(m),$

$A_2(m-1), n_{A_2}, Y(m) = 0; \boldsymbol{\eta})$

$= \eta_0 + \eta_1 A_1(m-1) + \eta_2 (L_2(m-1))^2 + \eta_3 (L_1(m-1))^2 + \eta_4 (A_1(m) L_1(m))$

$+\eta_6 (L_1(m) L_2(m)) + \eta_7 (A_1(m) L_2(m)) + \eta_8 A_2(m-1)$

$+\eta_9 (A_2(m-1) L_1(m)) + \eta_{10} (A_2(m-1) L_2(m)) + \eta_{11} n_{A_2}$

if $A_2(m-1) = 0$ or $m-1 = \tau_{A_2}$ **then**

$A_2(m) \sim$ Bernoulli(p_{A_2})

if $A_2(m) = 1$ **then**

$\delta_{A_2} \sim$ zero-truncated Poisson(9) (treatment duration after initiation)

$\tau_{A_2} \leftarrow m + \delta_{A_2}$

$A_2(m+1) : A_2(\max(\tau_{A_2}, M)) \leftarrow 1$

$n_{A_2} \leftarrow n_{A_2} + 1$

end if

end if

$H_m \leftarrow H_m + \exp\{\psi_1 A_1(m) + \psi_2 A_2(m)\}$

if $T^{\bar{0}} \geq H_m$

$Y_{m+1} \leftarrow 0$

else

$Y_{m+1} \leftarrow 1$

$T \leftarrow m + (T^{\bar{0}} - H_m) \times \exp\{-\psi_1 A_1(m) - \psi_2 A_2(m)\}$

end if

end for m

end for ID

S3. Supplementary figures and tables.

TABLE 1

Mean absolute bias (MAB), root mean square error (RMSE) and coverage probability (CP) for the estimates of ψ across 250 data replications with unaligned follow-up time points, using four weight estimators (i)-(iv) described in Section 3.4.

Weight estimators	ψ_1			ψ_2		
	MAB	RMSE	CP	MAB	RMSE	CP
(i)	.102	.104	.048	.092	.094	.060
(ii)	.063	.067	.104	.055	.059	.112
(iii)	.023	.029	.936	.020	.025	.940
(iv)	.016	.022	.952	.015	.019	.952

TABLE 2

The distribution of the estimated individual time-varying weights from one random replication of the "ragged" longitudinal data with unaligned time points, for the proposed JMSSM-CT versus JMSM-DT. To estimate the weights, the random forests was used for JMSM-DT and four approaches (i)-(iv) (Section 3.4) were used for JMSSM-CT.

Methods	Weight estimators	Distribution of estimated weights				
		Minimum	First quartile	Mean	Third quartile	Maximum
JMSSM-CT	(i)	0.23	0.89	1.05	1.23	5.34
	(ii)	0.40	0.98	1.01	1.07	4.28
	(iii)	0.52	0.88	1.00	1.09	2.99
	(iv)	0.68	0.95	1.00	1.05	2.36
JMSM-DT	Random forests	0.43	0.90	1.03	1.11	3.65

S3.1. *Additional simulation results.*

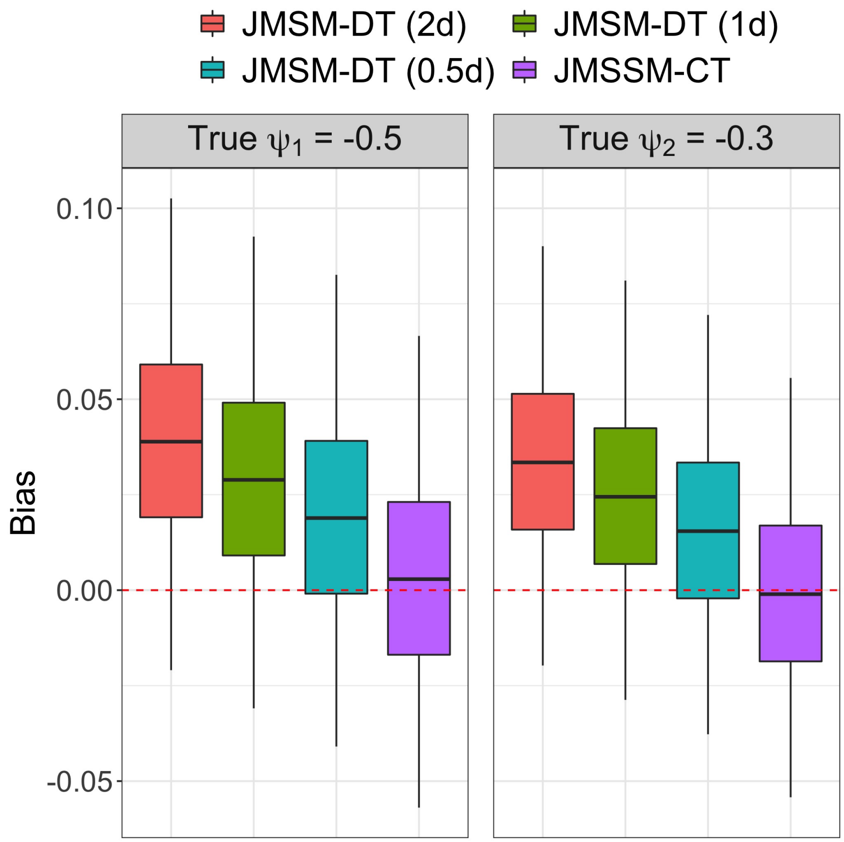

Figure 1: The distributions of biases across 250 simulation replications of the ragged longitudinal data with unaligned time points, in estimating the parameters ψ_1 and ψ_2 using the proposed JMSSM-CT method and the comparison method JMSM-DT. When implementing JMSM-DT, the follow-up time was respectively discretized into time intervals of length 0.5, 1 and 2 days, as the method requires aligned measurement time points.

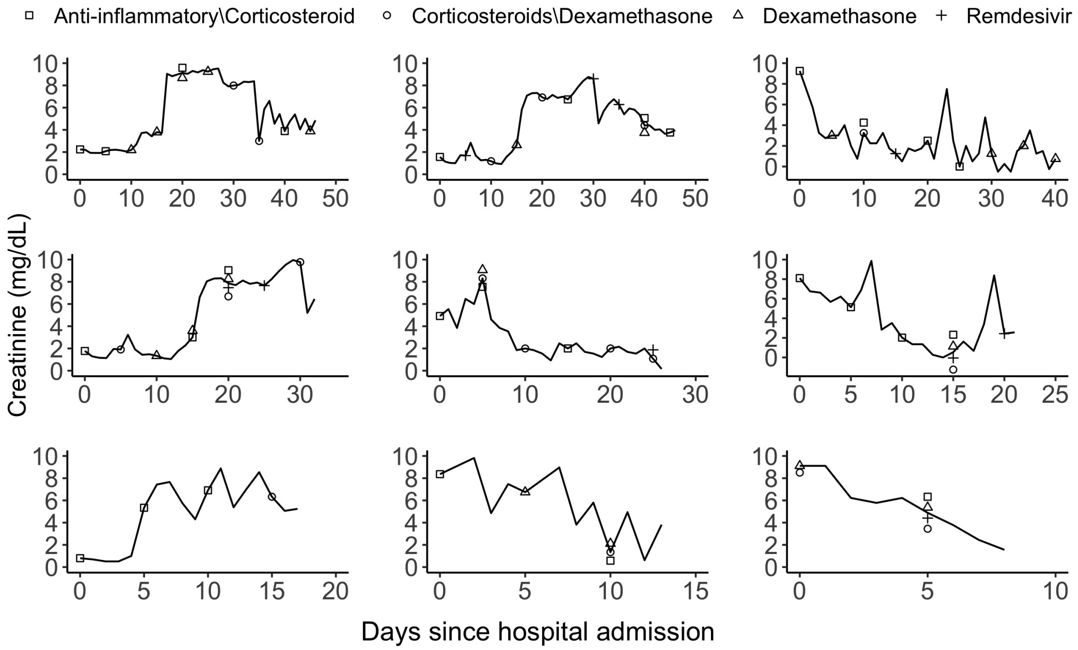

Figure 2: Trajectories of serum creatinine levels over the course of hospital stay for 9 randomly chosen patients. Symbols represent the types of treatment classes received by a patient at a given time.

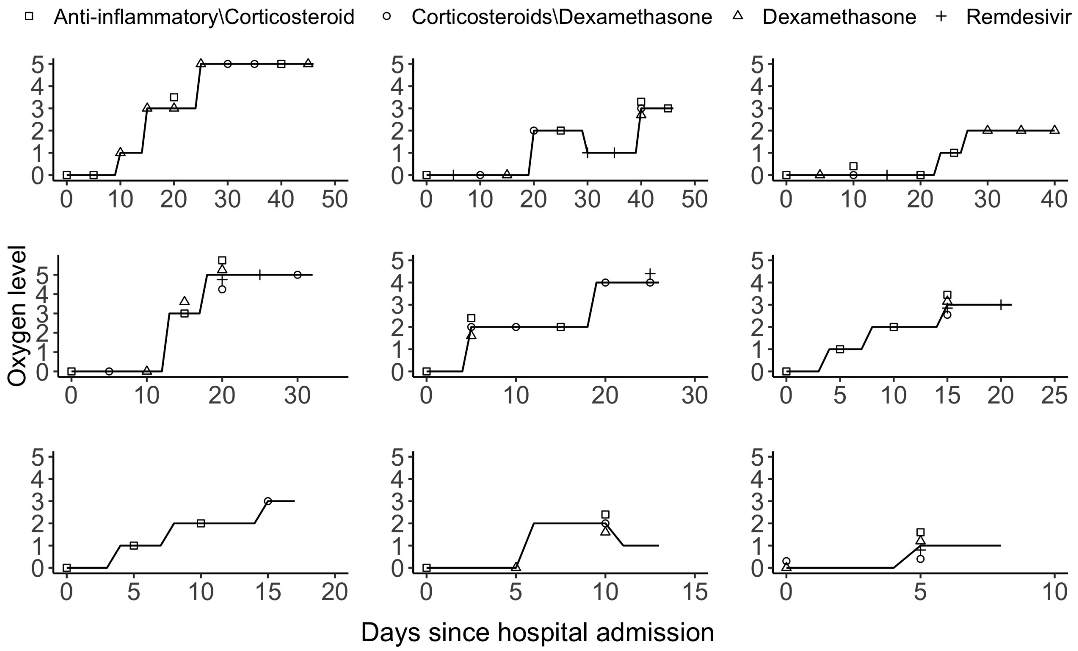

Figure 3: Trajectories of patient oxygen levels over the course of hospital stay for 9 randomly chosen patients. Symbols represent the types of treatment classes received by a patient at a given time.

S3.2. *Treatment classes for COVID-19.* In Table 3, we provide detailed definitions of the four treatment classes for COVID-19 whose comparative effectiveness on in-hospital death was investigated in Section 6.

TABLE 3
Definitions of four treatment classes for COVID-19. iv:intravenous; po: by mouth.

Treatment classes	Medication (route)
Dexamethasone	Dexamethasone (iv), Dexamethasone (po)
Remdesivir	Remdesivir (iv)
Corticosteroids other than dexamethasone	Hydrocortisone (po), Hydrocortisone (iv), Methylprednisolone (po), Methylprednisolone (iv), Prednisolone (iv), Prednisone (po), Prednisone (iv)
Anti-inflammatory medications other than corticosteroids	Alpha-1-Proteinase Inhibitor (iv), Anakinra (iv), Azathioprine (po), Belatacept (iv), Dexamethasone (iv), Dexamethasone (po), Eculizumab (iv), Envarsus (iv), Sarilumab (iv), Gengraf (po), Gengraf (iv), Hydrocortisone (po), Hydrocortisone (iv), Ibrutinib (po), Immune Globulin (iv), Infliximab (iv), Methylprednisolone (po), Methylprednisolone (iv), Montelukast (po), Prednisolone (iv), Prednisone (po), Prednisone (iv), Ruxolitinib (po), Tocilizumab (iv), Apremilast (po), Celecoxib (po), Dasatinib (po), Everolimus (iv), Gemtuzumab (iv), Ibuprofen (iv), Ibuprofen (po), Ifosfamide (iv), Leflunomide (po), Mesalamine (iv), Methotrexate (iv), Methylphenidate (iv), Mycophenolate (iv), Naproxen (po), Rituximab (iv), Sulfasalazine (po), Tacrolimus (iv), Pacritinib (po), Risankizumab (iv), Daratumumab (iv), Talquetamab (iv)

S3.3. *Patient oxygen levels.* We describe how patient oxygen levels are categorized based on the use of ventilator in Table 4.

TABLE 4
Definitions of patient oxygen levels based on the use of ventilator

Patient oxygen level	Ventilator status
0	Room air
1	Cannula
2	Mask, Blow-by, Face tent, Oxyhood, Non-rebreather, RAM cannula
3	Continuous positive airway pressure machine, High flow nasal cannula, Hudson prongs
4	Bilevel positive airway pressure machine, Tracheostomy mask
5	Tracheotomy, Transtracheal oxygen therapy, Ventilator, Endotracheal tube, T-shaped tubing connected to an endotracheal tube, Nasal synchronized intermittent mandatory ventilation

TABLE 5
Baseline characteristics of patients from the COVID-19 data. Summary statistics are represented as mean (standard deviation [SD]) for continuous variables and No. (%) for discrete variables.

Characteristics	$N = 11286$
Age (years), mean (SD)	64.59 (18.16)
Gender N (%)	
Male	6137 (54.4)
Female	5149 (45.6)
Race, N (%)	
White	3241 (28.7)
Black	2829 (25.1)
Asian	647 (5.7)
Others	4569 (40.5)
Ethnicity N (%)	
Hispanic	2972 (26.3)
non-Hispanic	8314 (73.7)
Asthma N (%)	
Yes	1082 (9.6)
No	10204 (90.4)
COPD N (%)	
Yes	734 (6.5)
No	10552 (93.5)
Hypertension N (%)	
Yes	5593 (49.6)
No	5693 (50.4)
Cancer N (%)	
Yes	1161 (10.3)
No	10125 (89.7)
Coronary artery disease N (%)	
Yes	2227 (19.7)
No	9059 (80.3)
Diabetes N (%)	
Yes	2809 (24.9)
No	8477 (74.1)
Smoking status N (%)	
Current	598 (5.3)
Former	2597 (23.0)
Never	6350 (56.3)
Unknown	1741 (15.4)
Hospital site N (%)	
Mount Sinai Brooklyn	1671 (14.8)
Mount Sinai Petrie	771 (6.8)
Mount Sinai Queens	1565 (13.9)
Mount Sinai St. Luke's	1828 (16.2)
Mount Sinai West	1304 (11.6)
Mount Sinai Main Hospital	4147 (36.7)

Abbreviations: SD = standard deviation;

REFERENCES

ANDERSEN, P. K., BORGAN, O., GILL, R. D. and KEIDING, N. (1993). *Statistical models based on counting processes*. New York: Springer-Verlag.

BINDER, D. A. (1992). Fitting Cox's proportional hazards models from survey data. *Biometrika* **79** 139–147.

BREIMAN, L. (2001). Random forests. *Machine Learning* **45** 5–32.

BRUMBACK, B. A., HERNÁN, M. A., HANEUSE, S. J. and ROBINS, J. M. (2004). Sensitivity analyses for unmeasured confounding assuming a marginal structural model for repeated measures. *Statistics in Medicine* **23** 749–767.

COLE, S. R. and HERNÁN, M. A. (2008). Constructing inverse probability weights for marginal structural models. *American Journal of Epidemiology* **168** 656–664.

FU, W. and SIMONOFF, J. S. (2017). Survival trees for left-truncated and right-censored data, with application to time-varying covariate data. *Biostatistics* **18** 352–369.

KARIM, M. E., PETKAU, J., GUSTAFSON, P., TREMLETT, H. and GROUP, T. B. S. (2017). On the application of statistical learning approaches to construct inverse probability weights in marginal structural cox models: hedging against weight-model misspecification. *Communications in Statistics-Simulation and Computation* **46** 7668–7697.

KARIM, M. E., PETKAU, J., GUSTAFSON, P., PLATT, R. W., TREMLETT, H. and GROUP, B. S. (2018). Comparison of statistical approaches dealing with time-dependent confounding in drug effectiveness studies. *Statistical Methods in Medical Research* **27** 1709–1722.

LEBLANC, M. and CROWLEY, J. (1992). Relative risk trees for censored survival data. *Biometrics* **48** 411–425.

SHU, D., YOUNG, J. G., TOH, S. and WANG, R. (2021). Variance estimation in inverse probability weighted Cox models. *Biometrics* **77** 1101–1117.

XIAO, Y., ABRAHAMOWICZ, M. and MOODIE, E. E. (2010). Accuracy of conventional and marginal structural Cox model estimators: a simulation study. *The International Journal of Biostatistics* **6**.

YAO, W., FRYDMAN, H., LAROCQUE, D. and SIMONOFF, J. S. (2020). Ensemble Methods for Survival Data with Time-Varying Covariates. *arXiv preprint arXiv:2006.00567*.
